# Supplementary material for: Model of multidisciplinary teamwork in hip fracture care: a qualitative interview study
Source: BMJ Open. 2024 Feb 27;14(2):e070050. doi: 10.1136/bmjopen-2022-070050 (PMC10900388; doi:10.1136/bmjopen-2022-070050)
Supplement: Supplementary data [file bmjopen-2022-070050supp001.pdf]

Supplementary material: Interview topic guide

Aims and objectives

- To investigate how hip fracture services are provided in different hospital settings and factors that help/ hinder ability to deliver care
- This study will provide us with information that can be used to improve the delivery of hip fracture services and reduce variation in care

Topics covered will be:

- Find out a bit about you
- Organisation of the care pathway
- Barriers and facilitators to service delivery
- Close

Part 1: About you

- Job title
- Part time/ full time/ %FTE
- Professional background (years since qualifying and progression)
- Years since qualifying for this role
- Years spent working in this service
- Age
- Date of birth
- Gender identify with
- Describe own role in delivering hip fracture care (before Covid)
    - Face to face duties
    - Administrative / organisational duties
    - Management duties

Part 2: Care pathway before Covid-19

- Things that worked well/ not so well/ improvements.

  Examples of components of care

  - Ambulance hand over
  - Emergency department factors [NB: Fast-tracking to ward]
  - Imaging services
  - Orthogeriatric services
  - Pre-operative care
  - Theatre and anaesthetic services
  - Orthopaedic & Trauma Services
  - Inpatient physiotherapy services and rehabilitation
  - Training/ confidence in the manual handling of patients
  - Links to dementia care, mental health services, diabetes care, dietetics, palliative care
  - Discharge systems and links to intermediate care
  - Temporary rehabilitation / care home provision
  - Post-discharge queries from patients
  - Re-admission processes for potential complications
  - Fracture Liaison Services/ secondary fracture prevention pathways
  - Falls services
  - Patient information and education
  - Community physiotherapy services and rehabilitation
  - Relationships with care homes – infection control protocols for new placements and return of patients to their original care homes

Part 3: Barriers and facilitators to service delivery before COVID-19

Things that worked well/ not so well/ improvements:

Communication

- How well did everyone communicate within service?
    - Within team
    - Other colleagues in MDT
    - Links with other services, e.g. dementia care, mental health services, diabetes care, palliative care
    - Community services and rehabilitation

  [Probes: MDT/ morbidity and mortality meetings, trauma rounds, multi-disciplinary paperwork, shared protocols, shared governance meetings, central coordinator]

  - What worked well/ not so well/ improvements

- Department management/ senior management

  - What worked well/ not so well/ improvements

- External organisations
    - Primary care
    - Community services and rehabilitation
    - Social care services

Relationships

- What does a 'good' working relationship mean to you?
- How well did everyone work together
    - Within team
    - Other colleagues in MDT
    - Department management/ senior management
- What sorts of things contributed to good working relationship with colleagues/

made it more difficult?

- Improvements

Commitment to delivering care

- To what extent did your colleagues do what was expected of them?

  [Probes: Extent to which the 36 hour target for hip fracture operation was pushed, extent individuals adhered to protocols]

  - Why do you think this was?

- How engaged/ enthusiastic do you think your colleagues were?

  - What helped/ made it more difficult?

Access to resources

- To what extent did you have access to all the resources needed to carry out work?

  [Probes: staff vacancies, theatre capacity, surgeon capacity, workload and material resources, access to medical and social care data],

  - If not, why not?
  - What did you do to address this challenge?

- To what extent did you/ team have access to the training and information needed to carry out work?

  [Probes: training, evidence base, understanding what is required of them]

- Training/ mentorship programmes in place

- What worked well/ not so well/ improvements

- How much of a priority do you feel that hip fracture care was for the?

  - Hospital Trust
  - Department
  - Boss

  - Why/ why not priority?

- Strategies to engage senior management

Service delivery over time

- Example quality improvement project
  - How did change occur?
- Example when you tried to initiate change but couldn't
  - Why?
- What monitoring and evaluation processes are in place?

[Probes: national initiatives, internal audit and governance processes, review of cases in morbidity and mortality meetings]

- What is the impact of these monitoring and evaluation processes on service delivery?

[Probes: Are these fed back to staff, do they effect change]

- Example of local clinical audit – why initiated?
- What clinical governance activities are in place for hip fracture care?

[Probes: risk management, transparency, quality improvement, mortality reviews, strategies to review clinical incidents/ complaints]

How useful are they?

Part 4: Close

1. If you could change one thing about your (pre-Covid) service, what would it be?
2. Are there any other things or insights that you would like to tell me about today?
3. Do you have any questions for me about the project?
4. Thank them
5. Reaffirm confidentiality
