# Supplementary material for: Model of multidisciplinary teamwork in hip fracture care: a qualitative interview study
Source: BMJ Open. 2024 Feb 27;14(2):e070050. doi: 10.1136/bmjopen-2022-070050 (PMC10900388; doi:10.1136/bmjopen-2022-070050)
Supplement: Supplementary data [file bmjopen-2022-070050supp002.pdf]

Supplementary material: A 'how to' guide for effective multi-disciplinary team working in hip fracture care

Delivering hip fracture care requires multi-disciplinary teamwork, including effective communication and coordination across a range of specialties and professional roles including emergency medicine, orthopaedic surgery, anaesthetics, geriatric medicine, rehabilitation and nurses. The range of the multidisciplinary team involved reflects the complex needs of patients with hip fracture. Clinical guidelines recommend timely hip fracture care, with when needed, rapid transfers of care between professionals. Streamlining multi-disciplinary care is crucial to the delivery of safe and effective patient care.

There are four components needed for coordinated multi-disciplinary team (MDT) working. These are all underpinned by one central concept: shared responsibility. The responsibility for delivering these components lies with *all members* of the MDT.

Action 1: Clearly define roles and responsibilities of each member of the MDT

Roles and responsibilities are more than what is simply listed in a job description. Each member of the MDT needs to understand the other MDT member's roles and responsibilities. Hence, roles and responsibilities need to be appreciated and explained during induction processes.

Roles and responsibilities need to be co-agreed between an individuals' line manager, within the MDT and outlined in personalised job plans. These job plans should be reviewed at annual appraisals with modifications made according to MDT developments/ progress.

The clinical lead needs to have oversight of all roles and responsibilities in order to identify gaps in the pathway of hip fracture care, so as to manage the skillset within the MDT to ensure those gaps are filled.

Action 2: Set up systems to ensure clear, timely MDT communication

Hip fracture patients have complex medical and surgical needs and are often acutely unstable. Therefore, MDT communication must be dynamic and efficient with the capacity for immediate response from senior team members when necessary. Systems are enhanced by the presence of trauma coordinators and discharge coordinators with defined roles and responsibilities.

Face-to-face communication events (verbal)
Systems need to be established for effective communication events with those attending pre-agreed. Communication events should begin promptly, using the same standardised

format each time. Job plans need to permit attendance. Examples of communication events include daily trauma meetings, early morning board rounds ('huddles') and pre-arranged discharge planning meetings. Short meetings are more efficiently conducted whilst standing, where ability permits.

Six principles of effective MDT communication events include:

1. Ensure representation from the key disciplines
2. Establish patient centred goals
3. Bring opinions together openly
4. Agree an overall aim, next steps with time frames
5. Allocate responsibilities
6. Communicate/ document outputs

Written MDT communication

Written MDT documentation should be appropriately co-designed to capture all relevant information. All patient admissions should be clerked using a standardised proforma co-agreed and designed by the MDT with sections addressing trauma at other skeletal sites (besides the hip), nerve block use, osteoporosis management and orthogeriatric and orthopaedic senior reviews. Documents need to be available to the wider MDT.
Examples of documents/ proformas that can assist the delivery of care include:

- Structured admission clerking proforma
- Structured post-operative junior doctor review (for patients returning to the ward from theatre)
- Structured falls and bone health assessments

Examples of documents are available on the National Hip Fracture Database (NHFD):
https://www.rcplondon.ac.uk/projects/outputs/national-hip-fracture-database-nhfd-improvement-repository

Electronic MDT communication

Smart Phones have made radio pagers redundant. All staff should have access to a Smart Phone. Information can be managed securely through e.g. Siilo for patient specific information. WhatsApp can be used for team communication, but not patient specific information. Mobile phone numbers should be routinely shared within the MDT. Appropriate groups should be set-up.

Hospitals need to invest in electronic communication systems e.g. (i) to enable trauma wards to have live monitoring of theatre activity, to plan prompt transfers to and from theatre, and (ii) routine access to primary care records. Templated discharge summaries, e.g. with use of standardised phrases, can improve efficiency and increase communication at discharge.

Hip fracture patients are best managed on a hip fracture ward where all members of the MDT can be co-located and thus easily found for spontaneous communication, with nearby office space. Timetabling of consultant ward rounds and daily ward activities should be shared amongst the whole team and adhered to.

Action 3: Establish inter-disciplinary principles of working

Healthcare teams can be 'multidisciplinary ' or 'interdisciplinary'. Multi-disciplinary teams have separate roles and work within their own specific disciplines. Interdisciplinary teams work more closely together to deliver shared goals and some roles may overlap.

Where human resource permits, hip fracture care should be provided through a shared care model between orthopaedic and orthogeriatric services.

Orthopaedic, geriatric and anaesthetic specialist trainees should receive formal inter-disciplinary training to ensure mutual understanding of each other's roles, e.g. geriatric trainees should attend a trauma meeting and a hip fracture theatre session, whilst orthopaedic trainees should join an orthogeriatric ward round and geriatrician lead white board rounds. Similarly, an orthogeriatric trainee should spend time with an orthopaedic physiotherapist whilst the anaesthetic trainee should join the orthogeriatric ward round. The ultimate goal of interdisciplinary training is to ensure mutual understanding and professional support between roles.

All trainees should attend clinical governance meetings to understand governance structured necessary to support a complex pathway of patient care.

Examples of inter-disciplinary upskilling include:

- Nurses should be upskilled by physiotherapists to confidently mobilise patients post-operatively
- Emergency department doctors should be upskilled by anaesthetists to provide emergency nerve blocks
- Physiotherapists should be upskilled by occupational therapists to assist discharge planning
- Orthopaedic trainees should be upskilled by orthogeriatricians to assess cognitive capacity to consent

Agree cross-cover arrangements within clinical competencies, e.g. physiotherapy and occupational therapy co-working, medical and surgical junior doctors cross-covering, nursing staff empowered to mobilise patients.

Action 4: Establish collaborative MDT team leadership

Orthogeriatric (1), orthopaedic (2), anaesthetic (3) consultant hip fracture leads should have their role defined within their job plan. The orthopaedic and/or orthogeriatric consultant should identify as clinical (co-)lead for hip fracture services.

Strong clinical leadership is essential to co-ordinate effective MDT hip fracture care delivery. Formal leadership training is strongly encouraged.

A physiotherapy (4), nursing (5), occupational therapy (6) and pharmacy (7) discipline lead

for hip fracture care should be appointed.

All seven of these professionals have responsibility to attend clinical governance meetings and act as conduit for feedback from and to their individual specialist teams.

All disciplinary leads should consider succession planning and discuss this with the leadership team to manage leadership transitions smoothly and efficiently (no discipline should be without hip fracture leadership). Aim for early identification of successors with structured mentorship with staged handover.

Identify a hospital board executive responsible for bidirectional communication with the hip fracture clinical lead, who will champion hip fracture care at board level. All seven discipline-specific hip fracture leads should have met with the hospital board executive. The primary point of contact for the hospital board executive should be the hip fracture service clinical lead.

Action 5: Jointly agree the goals of the team and strategies to achieve these goals

Define MDT goals by jointly reviewing NHFD performance, bench-marked KPIs, and how these compare against national averages, as well as REDUCE toolkit targets and any specialty-specific internal audit/ QI data. It’s really important to get all members of the team in the room and to hear the voices and perspectives of each MDT discipline with no one specialty dominating the conversation. The clinical lead for hip fracture services holds the responsibility for coordinating this process.

Strategies to improve services should be structured into short, medium and long-term approaches and achievements routinely reviewed at clinical governance and morbidity and mortality (M&M) meetings with plans and progress clearly communicated to the hip fracture service hospital board executive. The REDUCE toolkit is designed to facilitate each hospital to develop their own individualised strategies towards service improvement.
