# Supplementary material for: Model of multidisciplinary teamwork in hip fracture care: a qualitative interview study
Source: BMJ Open. 2024 Feb 27;14(2):e070050. doi: 10.1136/bmjopen-2022-070050 (PMC10900388; doi:10.1136/bmjopen-2022-070050)
Supplement: Author's manuscript [file bmjopen-2022-070050.draft_revisions.pdf]

BMJ Open is committed to open peer review. As part of this commitment we make the peer review history of every article we publish publicly available.

When an article is published we post the peer reviewers' comments and the authors' responses online. We also post the versions of the paper that were used during peer review. These are the versions that the peer review comments apply to.

The versions of the paper that follow are the versions that were submitted during the peer review process. They are not the versions of record or the final published versions. They should not be cited or distributed as the published version of this manuscript.

BMJ Open is an open access journal and the full, final, typeset and author-corrected version of record of the manuscript is available on our site with no access controls, subscription charges or pay-per-view fees (http://bmjopen.bmj.com).

If you have any questions on BMJ Open's open peer review process please email
info.bmjopen@bmj.com

A model of multidisciplinary teamwork in hip fracture care: a qualitative interview study

Journal:	*BMJ Open*
Manuscript ID	bmjopen-2022-070050
Article Type:	Original research
Date Submitted by the Author:	17-Nov-2022
Complete List of Authors:	Drew, Sarah; University of Bristol, Translational Health Sciences<br>Fox, Fiona; University of Bristol, Bristol Medical School (PHS)<br>Gregson, Celia; University of Bristol, Musculoskeletal Research Unit; Royal United Hospital Bath NHS Trust, Older Person's Unit<br>Gooberman-Hill, Rachael; University of Bristol, Bristol Medical School
Keywords:	Organisation of health services < HEALTH SERVICES ADMINISTRATION & MANAGEMENT, Hip < ORTHOPAEDIC & TRAUMA SURGERY, QUALITATIVE RESEARCH

SCHOLARONE™
Manuscripts

I, the Submitting Author has the right to grant and does grant on behalf of all authors of the Work (as defined in the below author licence), an exclusive licence and/or a non-exclusive licence for contributions from authors who are: i) UK Crown employees; ii) where BMJ has agreed a CC-BY licence shall apply, and/or iii) in accordance with the terms applicable for US Federal Government officers or employees acting as part of their official duties; on a worldwide, perpetual, irrevocable, royalty-free basis to BMJ Publishing Group Ltd ("BMJ") its licensees and where the relevant Journal is co-owned by BMJ to the co-owners of the Journal, to publish the Work in this journal and any other BMJ products and to exploit all rights, as set out in our licence.

The Submitting Author accepts and understands that any supply made under these terms is made by BMJ to the Submitting Author unless you are acting as an employee on behalf of your employer or a postgraduate student of an affiliated institution which is paying any applicable article publishing charge ("APC") for Open Access articles. Where the Submitting Author wishes to make the Work available on an Open Access basis (and intends to pay the relevant APC), the terms of reuse of such Open Access shall be governed by a Creative Commons licence – details of these licences and which Creative Commons licence will apply to this Work are set out in our licence referred to above.

Other than as permitted in any relevant BMJ Author's Self Archiving Policies, I confirm this Work has not been accepted for publication elsewhere, is not being considered for publication elsewhere and does not duplicate material already published. I confirm all authors consent to publication of this Work and authorise the granting of this licence.

For peer review only

Multidisciplinary teamwork in hip fracture care

Title: A model of multidisciplinary teamwork in hip fracture care: a qualitative interview study

Corresponding author

Dr Sarah Drew

Research Fellow

Musculoskeletal Research Unit

Bristol Medical School, University of Bristol

Learning and Research Building, Level 1

Southmead Hospital

BS10 5NB

Mobile: + 44 (0)7891149828

Email: sarah.drew@bristol.ac.uk

Sarah Drew[1], MA, DPhil

Fiona Fox[1], BSc, PhD

Celia L Gregson[1], B.Med.Sci., M.B.,B.S., MSc, FRCP, PhD

Rachael Gooberman-Hill[1,2], MA, PhD

Author affiliations

1. Musculoskeletal Research Unit, Translational Health Sciences, Bristol Medical School, University of Bristol, Bristol, UK.

For peer review only - http://bmjopen.bmj.com/site/about/guidelines.xhtml

Multidisciplinary teamwork in hip fracture care

2. UK National Institute for Health Research Bristol Biomedical Research Centre, University Hospitals Bristol and Weston NHS Foundation Trust and the University of Bristol, Bristol, UK.

Word count 3995 (max 4000)

Keywords

Hip fractures, multidisciplinary teamwork, service delivery, qualitative

For peer review only

For peer review only - http://bmjopen.bmj.com/site/about/guidelines.xhtml

Multidisciplinary teamwork in hip fracture care

Abstract

Objectives

Hip fractures are common injuries older age with high mortality requiring multidisciplinary clinical care. Despite guidance, there is considerable variation in hip fracture services and patient outcomes; furthermore, little is known about how effective multidisciplinary working can be enabled. This study aimed to identity the core components of effective multidisciplinary teamwork in hip fracture care.

Design

The study comprised qualitative interviews with healthcare professionals delivering hip fracture care. Interviews were audio-recorded, transcribed and analysed abductively: material was coded inductively and grouped into higher level concepts informed by theories and teamwork related frameworks.

Setting

Four hospitals in England.

Participants

Participants were 40 healthcare professionals including orthopaedic surgeons, orthogeriatricians, physiotherapists and service managers.

Results

Results identified five components needed for effective multidisciplinary teamwork: (i) defined roles and responsibilities, (ii) information transfer processes, (iii) shared goals, (iv) shared responsibility, and (v) collaborative leadership. Defined roles and responsibilities were promoted through formal care pathways, reinforced through induction and training

For peer review only - http://bmjopen.bmj.com/site/about/guidelines.xhtml

Multidisciplinary teamwork in hip fracture care

with clear job plans outlining tasks. Information transfer processes facilitated timely information exchange to appropriate individuals. Well-defined, common purpose was hindered by complex inter-disciplinary professional relationships, particularly between orthogeriatric and orthopaedic staff, and encouraged through multidisciplinary team meetings and training. Clinical service leads were integral to bridging interdisciplinary boundaries. Shared responsibility for patients was encouraged by joint orthopaedic and orthogeriatric care models. Staff shared responsibility by assisting colleagues when needed. Mutual trust and respect were based on recognition of the value of different professional groups. Teamwork depended upon formal clinical leads with facilitative and motivational roles, and on centralised hospital leadership that created an environment supporting collaboration.

Conclusions

Common goals and a culture of shared responsibility for care delivery are fundamental to multidisciplinary care provision for hip fracture. Findings will inform development of clinical practice recommendations and training to build teamworking competencies.

Article summary

Strengths and limitations of this study

- This is the first study to understand and characterise the core components needed to deliver multidisciplinary teamwork for effective and efficient hip fracture care delivery for hip fracture.
- Qualitative study design and methods provided an in-depth understanding of care processes.

For peer review only - http://bmjopen.bmj.com/site/about/guidelines.xhtml

Multidisciplinary teamwork in hip fracture care

- The sample, comprising 40 participants drawn from four UK hospitals, provided sufficient data to address the research question and included a wide range of professionals with relevant experience in hip fracture care, providing information power.
- Data collection was conducted in hospitals that admitted moderate to large numbers of hip fracture patients. This may limit transferability of findings to smaller units.
- Using an abductive analysis approach, rather than transposing data onto an existing theory or framework, ensured the resulting model was data-driven and that data were not 'forced' into pre-defined constructs.

Funding statement

This work was supported by Versus Arthritis (reference 22086).

Competing interests

All authors declare that they have no competing interests.

For peer review only

Multidisciplinary teamwork in hip fracture care

Introduction

Globally, osteoporotic fractures pose a major challenge to patients and healthcare systems, with 4.5 million hip fractures expected to occur annually by 2050[1]. Hip fractures are most commonly sustained by older individuals and can be devastating with considerable impact on quality of life and a high risk of subsequent death[2, 3]. Provision of hip fracture care is costly to healthcare providers, incurring $5.96 billion in direct medical costs each year in the United States and £1.2 billion in the United Kingdom (UK)[4]. Comprehensive multidisciplinary hip fracture care pathways reduce postoperative complications and mortality[5]. To make recommendations about how to further enhance the quality care of care for people who have had hip fracture, there is a need to understand how healthcare professionals implement care pathways. Previous studies have explored how patients and carers engage with hip fracture care, and qualitative findings highlight the emotional and practical challenges associated with hip fracture[6-8]. However, research to date has not explored care delivery from the viewpoint of professionals.

In the UK, thorough guidance has been published on the management of hip fracture[9-11]. Despite this, considerable variation exists in the organisation of care pathways[10, 12, 13] and in patient outcomes[13]. Effective hip fracture care requires communication and coordination across a range of specialties and professional roles including emergency medicine, surgery, anaesthetics, geriatric medicine and rehabilitation[9, 10]. This multidisciplinary care reflects patients' complex needs that may include multiple pre-existing conditions[14]. Guidelines recommend prompt care, with rapid transfers of care between professionals[9, 14]. High quality multidisciplinary care is crucial to delivery of safe and effective services. Despite the commonality of multidisciplinary working, little is known

For peer review only - http://bmjopen.bmj.com/site/about/guidelines.xhtml

Multidisciplinary teamwork in hip fracture care

about the key ingredients needed to optimise collaborative teamwork and thereby enable efficient care pathways that achieve positive patient outcomes.

Effective multidisciplinary teamwork is known to be necessary in the delivery of clinical care for a number of health conditions and specialties, such as cancer[15], dementia[16] and in older people's services[17]. 'Teamwork' can be defined as 'a set of inter-related thoughts, actions, and feelings of each team member that are needed to function as a team and that combine to facilitate coordinated, adaptive performance and task objectives resulting in value-added outcomes'[18]. Collaborative teamwork exists on a continuum of professional autonomy and integrative working. Three broad models describe collaborative teamwork: 'multidisciplinary', whereby multiple specialties with separate roles work within their own disciplines; 'interdisciplinary' that involves working together to deliver shared goals with some blurring of disciplinary boundaries; and 'transdisciplinary' where professional roles are shared[19]. Research has highlighted how different tasks across clinical disciplines shape how teams are organised and work together[20]. Hence, there is need to understand and characterise effective teamwork within the hip fracture care pathway.

For peer review only

Theories and frameworks guide professionals in the organisation and evaluation of the processes used in multidisciplinary team communications, training and leadership. Some frameworks are generalisable across several areas[18, 20-22], others have been developed for specific clinical disciplines[23]. Frameworks have identified diverse factors that promote clinical teamwork, including a shared understanding of team goals[18, 19, 21], effective information exchange[18], effective leadership[17, 18, 21, 23] and adaptability of services to meet changing demands[18, 24, 25]. Ellis and Sevdalis build on existing frameworks to

For peer review only - http://bmjopen.bmj.com/site/about/guidelines.xhtml

Multidisciplinary teamwork in hip fracture care

identify core technical and non-technical skills to facilitate effective multidisciplinary teamwork in geriatric medicine[17]. Non-technical skills include social, cognitive and personal resource skills. Strategic domains to enhance multidisciplinary working are skills (*e.g.*, good leadership), processes (*e.g.*, good documentation governance) and values (*e.g.*, prioritising patient needs and respect for colleagues). Evidence from behavioural research into multidisciplinary teamwork suggests that if any of these three components is missing, teamwork will be unsuccessful[17].

Given the importance of multidisciplinary teams in hip fracture care and previous learning from other fields, we aimed to develop a model of the core components needed to deliver effective multidisciplinary teamwork in hip fracture care in England. Our model provides a basis for future training, skills development and assessment of multidisciplinary teamwork for hip fracture care inside and outside of the UK. Furthermore, hip fracture care is an exemplar condition and findings may be used to develop complex care pathways in other disciplines.

Methods

We used qualitative interviews to collect and interpret insights from health professionals engaged in delivery of hip fracture care in hospitals in England.

Sample

Hospital sites

For peer review only - http://bmjopen.bmj.com/site/about/guidelines.xhtml

Multidisciplinary teamwork in hip fracture care

Healthcare professionals involved in the organisation and delivery of hip fracture were sampled from four National Health Service (NHS) hospitals across England. The NHS provides almost all hip fracture care in England, free to patients at the point of receipt. Purposive sampling was used to identify study sites[26], with variation in geography, number of hip fracture presentations per year, service configuration and audited hip fracture outcomes[27]. Three hospitals were urban and one rural. One hospital admitted a large number (>75th percentile), and three moderate (between 25th-75th percentile) numbers of hip fracture patients annually. This enabled us to capture the experiences of those delivering different models of care. Hospitals are identified by pseudonyms to retain anonymity ('Springhill', 'Radford', 'Maplegrove' and 'Newbridge').

Participants

Forty interviews were conducted with healthcare professionals across the four hospitals. Staff were purposively sampled using a criterion approach, targeting healthcare professionals from across the care pathway, including orthopaedic surgeons, anaesthetists, orthogeriatricians, physiotherapists and occupational therapists, nurses, trauma coordinators and service managers[28].

Potential participants were identified by a designated contact working within the service at each hospital site. Staff were invited to participate in the study by email, followed-up with reminder emails a minimum of two weeks later. Snowball sampling was also used[29], with participants recommending other professionals involved in service delivery. In total, 75

For peer review only - http://bmjopen.bmj.com/site/about/guidelines.xhtml

healthcare professionals were approached to take part in the study, of whom, 40 (53%) agreed to take participate. The remainder either declined or were unavailable. The final sample size was adequate to address the study aim and involved appropriate participants across the care pathway. Information power was achieved, whereby no new themes or subthemes were identified in the data[30].

Ethical approval

Ethical approval was provided by the Faculty of Health Sciences University of Bristol Research Ethics Committee (Ref: 108284) and by the NHS Health Research Authority (20/HRA/71). Written informed consent was provided by all participants, confirming understanding of voluntary participation and potential publication of anonymised interview quotations. NHS settings (Trusts) for the four study sites each provided research governance approval.

Data collection

Two qualitative researchers (FF and SD) conducted interviews by telephone or teleconferencing technology (*e.g.,* Microsoft teams), necessitated by Covid-19 pandemic restrictions. Interviews, lasting 45 to 90 minutes, were guided by a topic guide based on domains agreed collaboratively with healthcare professionals involved in delivery and organisation of hip fracture services. Part 1 focused on barriers and facilitators to the implementation of care. Part 2 covered multidisciplinary communication and cooperation including questions about access to resources, monitoring and evaluation (Supplementary

For peer review only - http://bmjopen.bmj.com/site/about/guidelines.xhtml

Multidisciplinary teamwork in hip fracture care

material). Interviews were flexible enabling exploration of emerging ideas[31]. Due to its relevance across domains, questions relating to teamwork were covered in all domains.

Data analysis

Interviews were audio-recorded, transcribed and anonymised to remove any identifying information and participants were assigned pseudonyms. Data were analysed using an abductive approach conducted in several stages[32]. Abduction is an approach to theorising that involves using research findings to develop new theories that build upon existing theories and frameworks. Transcripts were imported into NVivo qualitative data management software[34] and coded using an inductive thematic approach[35]. Analysis focused on aspects of the data relating to teamwork, which featured across all interviews. Codes were grouped into higher level concepts, informed by existing theories and frameworks around teamwork[17, 18, 21]. A model was then constructed to illustrate how core concepts contributed to multidisciplinary teamwork for hip fracture care (See Figure 1). To ensure rigor, 40% transcripts were independently analysed in duplicate (by SD and FF) and themes reviewed and refined to reach an agreed code list[35].

Patient and public involvement

To refine the study design and data collection materials, we held three focused group meetings with members of ‘The Patient Experience Partnership in Research’ (PEP-R) group. PEP-R is the dedicated musculoskeletal patient involvement group based in the Musculoskeletal Research Unit, University of Bristol[36]. This included a final meeting to advise on interpretation and relevance of findings.

For peer review only - http://bmjopen.bmj.com/site/about/guidelines.xhtml

Multidisciplinary teamwork in hip fracture care

Supplementary material: Topic guide

Results

The 40 interviewees comprised 6 orthopaedic surgeons; 6 anaesthetists; 7 orthogeriatricians; 3 physiotherapists; 4 occupational therapists; 1 emergency medicine consultant and 1 emergency department nurse; 3 advanced nurse practitioners (in orthogeriatrics, emergency medicine and orthopaedics and trauma); 1 ward manager, 1 matron, 2 service managers; 1 senior operating department practitioner (ODP); 1 discharge coordinator; 1 trauma coordinator; 1 orthopaedic registrar and 1 junior doctor. Between 5 and 15 participants took part from each hospital ('Springhill'=9, 'Radford'=11, 'Maplegrove'=15 and 'Newbridge'=5). Participants' characteristics are summarized by hospital to maintain anonymity (Table 1).

Table 1: Characteristics of healthcare professionals interviewed

**Professional Role**	**Number of professionals interviewed across each hospital**				**Time spent working in role**
	**01 Springhill**	**02 Radford**	**03 Maplegrove**	**04 Newbridge**	
Consultant geriatrician / Orthogeriatrician	4	2	1	1	3–18 years
ED Consultant	1				5 years
Band 8 ANP Emergency Department		1			6 years
Anaesthetist	1	3	1	1	7–25 years
Orthopaedic surgeon		2	4		5–11 years
Orthogeriatric Advanced Nurse Specialist	1				14 years
Physiotherapist			1	1	3–5 years

For peer review only - http://bmjopen.bmj.com/site/about/guidelines.xhtml

Multidisciplinary teamwork in hip fracture care

Occupational Therapist	1	1	1	1	2–8 years
MSK Matron	1				7 years
Service Manager		1	1		5 years
Ward Manager		1			5 years
Senior Theatre Practitioner			1		8 years
ANP			2		13–17 years
Orthopaedic Registrar			1		5 years
FY2 Doctor			1		3.5 months
Discharge coordinator			1		19 years
Trauma coordinator				1	5 years
**Total**	**9**	**11**	**15**	**5**	

Abbreviations: ANP; Advanced Nurse Practitioner, ED; Emergency Department, FY2; Foundation Year 2, MSK; Musculoskeletal.

We identified five core components of effective multidisciplinary teamwork for hip fracture care: (i) defined roles and responsibilities, (ii) information transfer processes, (iii) shared goals, (iv) shared responsibilities, and (v) collaborative leadership (Figure 1). We approach the results through a series of propositions.

Figure 1: Core components of effective multidisciplinary hip fracture care

i. Defined roles and responsibilities

Proposition 1: Successful multidisciplinary teamwork depends on clarity in roles and responsibilities within the team.

Clear roles and responsibilities were achieved by using hip fracture protocols and formalised care pathways that structured parts of the care process. These were undermined when key components of the pathway were missing that included management of analgesia, patient

For peer review only - http://bmjopen.bmj.com/site/about/guidelines.xhtml

For peer review only

optimisation for surgery and falls assessment. The extent to which protocols and pathway documents were used depended upon ‘buy in’, which was variable, and partially related to staff seniority, with greater resistance from staff towards the end of their careers. Reasons for variation included disagreement around patient management and desire for greater professional autonomy. Participants provided examples where confusion and disagreements over responsibilities created tensions within the team. Comprehensive induction and training on protocols and pathways, particularly for new staff and rotating junior doctors, provided clear information on “what to expect or adhere to”. Clarity was also achieved by clear job planning that outlined the parameters of professional roles and clarified expectations and responsibilities.

ii. Information transfer processes

Proposition 2: Successful multidisciplinary teamwork depends on processes to facilitate the effective transfer and exchange of information to the ‘right’ individuals at the ‘right’ time.

One of the primary objectives of care coordination was the effective transfer of information to inform patient management and decision-making. This was achieved via formal mechanisms including multidisciplinary team meetings, ward rounds and documentation, shared information systems, and informal and spontaneous communication.

Multidisciplinary team meetings in hospital sites took a range of forms including trauma meetings, focused ‘board rounds’, ‘huddles’, and discharge planning meetings. Participants identified characteristics of effective multidisciplinary team meetings including establishing

For peer review only - http://bmjopen.bmj.com/site/about/guidelines.xhtml

Multidisciplinary teamwork in hip fracture care

patient goals, regular attendance by a range of specialties and effective management such as time management and documentation of outputs. However, these were threatened by lack of attendance from key professional groups. This was due to competing commitments and a perception that attendance was "a waste of time" since time was better spent with patients, or because meetings often repeated the same issues without resolution. Multidisciplinary team documentation was vital to transfer patient information. However, patient documentation was not always accessible to relevant teams at key points in the care pathway, nor completed consistently or appropriately designed to capture all relevant information. Information transfer was facilitated using shared information systems such as patient tracking systems, 'ward boards' with key patient information, and electronic patient records. Communication processes were undermined where IT systems did not cross organisational boundaries either within secondary care, or with external organisations such as primary care and community services, including lack of access to primary care records. Multidisciplinary team ward rounds and 'huddles' provided opportunities to create shared knowledge and problem-solve. Participants advocated varied modes of communication to bring together professional groups at different stages of the care pathway.

Shared workspaces promoted frequent and spontaneous verbal communication, including nearby office space. Placing patients on a specific hip fracture ward facilitated this exchange as healthcare professionals were concentrated together. Spontaneous communication was also achieved using technologies such as WhatsApp, mobile 'phone and email. However, effectiveness depended on the interpersonal skills of staff. Verbal communication was also hindered by hierarchical relationships within the team. While some senior members of staff emphasised lack of hierarchy on wards, one Advanced Nurse Practitioner felt that ward staff

For peer review only - http://bmjopen.bmj.com/site/about/guidelines.xhtml

were "wary" of discussing patient management with consultants. 'Coordinators' functioned as key points of contact, providing continuity and communication between professional groups at different stages of the care pathway on a daily basis. Formal, named coordinator roles included trauma and discharge coordinators and hip fracture nurse specialists. Informal coordinating roles provided teams with an additional point of contact. In one site this included use of a named member of staff with responsibility for hip fracture patients. However, over reliance on individuals meant that when they were away, communication and coordination was compromised.

Box 1: Defined roles and responsibilities and information transfer processes illustrative quotations

Defined roles and responsibilities

"We have a good communication right from the start [of the pathway] … it's very kind of clear cut who's responsibility is what." Sue, Band 8 advanced majors' practitioner, emergency department, Radford

"Not many people know what OT [occupational therapy] is and you've really got to fight sometimes about your professional role in things… It's that you're just having to keep fighting, 'No, that's not for us. That's for somebody else'." Lois, trauma and orthopaedic ward occupational therapist, Newbridge

"There was a complete lack of understanding from the trauma and orthopaedic consultants as to an expectation from them that orthogeriatrics was my biggest priority. And I was

For peer review only - http://bmjopen.bmj.com/site/about/guidelines.xhtml

Multidisciplinary teamwork in hip fracture care

saying, well it's not in my job plan to do it and there was a complete lack of understanding from them about that." Elaine, consultant geriatrician, Newbridge

"Well I think the protocols and pathways work really well if everybody adheres to it, but with having a large department with a variety of clinicians involved it's difficult to get a buy-in from everybody." Peter, consultant orthopaedic surgeon , Radford

"Trying to get everybody to sign up to say something will always happen, seems to be quite difficult at the moment.… It does just take time and the people who have recently come through training tend to be far more cohesive and wanting to have demonstrable pathways that can be audited and guide people who are more junior." Tania, consultant anaesthetist, Newbridge

Information transfer processes

"I'm involved in the daily trauma meetings every morning, so that is all the consultants, that's the service manager, that's the junior doctors and that's us as an ACP [Advanced Clinical Practice] team. And you talk about the patients…. Sometimes the consultants can talk over you and forget that you're actually telling them what they need to know." Jacqui, advanced nurse practitioner, Maplegrove

"I've got a bleep, they've got my mobile, so I'm contactable throughout the day. So people from wards will be ringing me to ask questions." Joanne, trauma coordinator, Newbridge

For peer review only - http://bmjopen.bmj.com/site/about/guidelines.xhtml

Multidisciplinary teamwork in hip fracture care

"I think sometimes that probably when some of the consultants or the anaesthetists come on the ward that sometimes [ward staff are] a bit wary of approaching them." Barbara, advanced nurse practitioner, Maplegrove

[The trauma coordinator] role has sort of, it's found itself floundering a bit so we don't intend to remove it, we just intend to enhance it and make it more clear what the role [is]. I Darren, service manager, Radford

iii. Shared goals

Proposition 3: Successful multidisciplinary teamwork depends on the existence of a shared and well-defined purpose within the team

Shared goals were undermined by complex dynamics between different professional groups, particularly geriatric and orthopaedic staff who participants felt had different patient priorities. Several orthogeriatricians described orthopaedics as a speciality that focused on "fixing" the fracture, compared with their holistic medical approach. The structural divisions in NHS Trusts that placed staff within different 'directorates' were seen as reinforcing professional differences and impeding communication. Engagement with shared goals was also challenging due to staff transience, particularly of junior doctors who worked on regular rotations.

Shared goals were encouraged through multiple processes. Daily multidisciplinary team meetings enabled staff to build shared goals and collaborative treatment plans for individual

For peer review only - http://bmjopen.bmj.com/site/about/guidelines.xhtml

Multidisciplinary teamwork in hip fracture care

patients and to hear "different points of view". Multidisciplinary team training provided opportunities for staff to share knowledge and understanding. This was particularly evident in the training, mentoring and support for junior staff and helped their engagement in patient care. Clinical leads were integral to bridging these professional boundaries and creating a shared professional identity across individual specialties. They did this by establishing shared priorities and goals in multidisciplinary team planning meetings, sharing monitoring and auditing results with colleagues across the care pathway and using findings to collaboratively drive service improvement. Communal efforts in monitoring and appraisal provided further opportunities to reinforce shared priorities and goals, such as reviewing National Hip Fracture Database data, a national audit program to benchmark services, mortality events, internal audits and having casual and informal discussions to discuss service improvement.

iv. Shared responsibility

Proposition 4: Successful multidisciplinary teamwork depends on a culture of shared responsibility for care delivery

A culture of shared responsibility for hip fracture care ensured all members of the multidisciplinary team were invested in care delivery. This was enabled through care models where patients were cared for jointly by orthopaedic surgeons and orthogeriatricians. Staff valued the assistance of colleagues in performing tasks when needed, particularly when they were overloaded. Shared responsibility was also evidenced by colleagues providing feedback to improve performance and taking on supervisory roles. Orthogeriatricians were

For peer review only - http://bmjopen.bmj.com/site/about/guidelines.xhtml

Multidisciplinary teamwork in hip fracture care

seen as having a key role in supervising and supporting advanced nurse practitioners, therapists and junior doctors (medical and surgical). There was evidence of blurring of traditional role boundaries, including in the upskilling of nurses in hip fracture care. This included their involvement in the mobilisation of patients which was crucial for progressing patient recovery during periods where therapists were not available, such as at weekends and of emergency department doctors by anaesthetists to provide nerve blocks.

Mutual trust and respect for one another's skills created an environment that encouraged staff to participate actively in the multidisciplinary team such as in meetings, via consultation with colleagues and constructive management of disagreements. Trust and respect were based on a recognition of the value of different professional groups. Familiarity with colleagues, both professionally and personally, helped to build this trust. Informal relationships were facilitated through shared workspaces. Mutual trust and respect were often cultivated over time.

Box 2: Shared goals and shared responsibility illustrative quotations

Shared goals

"Essentially, I think we all are bound by sort of evidenced based [care], I think that is a common sort of thing and we all want the best for our patients. And I think those are two things which bind us that we all want to do the right thing for the patients." Dheeraj, consultant anaesthetist, Radford

For peer review only

Multidisciplinary teamwork in hip fracture care

"It's one of our major battles [as orthogeriatricians], the fact that we struggle to engage [with orthopaedic surgeons]. We struggle with them to engage with us, because you know basically they fix them and then walk away but occasionally we need them back and it's an ongoing battle, always has been." David, orthogeriatric advanced nurse specialist, Springhill

"I'm not a fan of the divisional structure of hospitals. I think it's quite artificial and hospitals are trying too hard to align themselves like a corporation would… I think it can [impede communication]." Andy, consultant orthopaedic surgeon, Maplegrove

"Our top consultant that works on [the hip fracture] ward, she's very driven by being the best, and having the best outcome for our patients. So I think everyone has a bit of a collective drive, but I think the pathway, being on that ward and on the pathway is a mega drive. Because it's constantly evaluated." Jane, occupational therapist, Springhill

"Updating your team is massively important. If you explain to your team why you're doing something, what you'd like done and why you'd like it done, that really helps. It's just that you've got that shared aim at the end of it. If you're working towards the shared aim, I think things work much better." Lucy, emergency medicine consultant, Springhill

Shared responsibility

"When we're all talking together [as a team] sometimes we can go, 'Can you do this and we'll do that' and I suppose it's just supporting each other…. So again, sometimes it's just

For peer review only - http://bmjopen.bmj.com/site/about/guidelines.xhtml

Multidisciplinary teamwork in hip fracture care

like, 'If you do this then that gives me time to do that' and we work that way." Jessica, occupational therapist, Radford

"You've got to listen to everybody and without certain people in that team it won't work, so I form a good rapport with the social workers, placement of care hub, so that you know they can trust me and I can trust them." Alice, foundation year 2 doctor, Maplegrove

I think you need to appreciate each other in terms of what people bring. You need to recognise other people's strengths and whether they're best placed to talk about something or you are yourself. Andy, consultant orthopaedic surgeon, Maplegrove

"I think there has to be an element of respect [for colleagues from different teams] as well, that in the good old days, a consultant was king and the staff were just, you know the minions, working minions, working around them but nowadays there's more of an equality in the relationship. So, I would probably quite happily give an opinion of the Nurse on the ward, if she has a query about a patient, cause she sees the patient every day, so there's quite an open communication both ways." Lance, consultant orthopaedic surgeon, Radford

"At one point I did start going to the trauma meeting for a bit. And actually that was really interesting for me because I did realise, hearing them talk about how specialised their knowledge was actually about operations and the operative management of problems. And I found that really interesting because I thought gosh, you know, these people do know a lot about what they did." Elaine, consultant geriatrician, Newbridge

For peer review only - http://bmjopen.bmj.com/site/about/guidelines.xhtml

Multidisciplinary teamwork in hip fracture care

v. Collaborative leadership

Proposition 5: Successful multidisciplinary teamwork depends on effective, collaborative leadership across several specialties and levels

Successful multidisciplinary teamwork was dependent upon having nominated departmental leads for hip fracture care. These formal clinical leads had a facilitative and motivational role and centralised hospital leadership created an environment to support collaboration. Participants advocated a formal model of leadership that was concentrated in small, mixed speciality teams across orthopaedic, orthogeriatric, aesthetic and nursing divisions that contributed a range of skills and perspectives. Participants identified specialties missing at this level, including anaesthetists. Therapy staff felt excluded at this level and that their representation could promote more effective collaboration. Senior level leadership was seen as promoting commitment amongst colleagues at other levels.

Clinical service leads had a facilitative and motivational role in engaging healthcare professionals from a range of specialties and assembled teams to develop shared priorities and goals, joint protocols, audit priorities and mutually-owned quality improvement plans. Multidisciplinary team planning meetings were a key part of this process. Participants identified several attributes that enabled 'heroic' individuals to fulfil these roles, such as "passion" and "drive". The visibility of clinical service leads within the service and respect for their clinical judgement legitimised their position and influenced their success in achieving these aims. Hospitals with one strong clinical service lead emphasised the importance of

For peer review only - http://bmjopen.bmj.com/site/about/guidelines.xhtml

Multidisciplinary teamwork in hip fracture care

leadership development and succession planning to maintain stability and high performance within the team.

Effective leadership on the wards was dependent upon centralised leadership that created an environment to support collaboration. Support included providing sufficient capacity and resources for hip fracture care including capacity for staff to attend MDT meetings, supporting local leads to engage in service planning and training by building this into job plans and supporting service improvement initiatives and innovation to help provide a sense of shared purpose. Some participants expressed frustration that reimbursement from best practice tariffs for hip fracture care was not being reinvested into services to support development. Participants emphasised the need for designated hospital board executives who engaged in bidirectional communication with clinical service leads and championed hip fracture care to facilitate this. Several participants highlighted a lack of centralised leadership due to the transience of higher management roles and competing priorities within the Trust.

Box 3: Collaborative leadership illustrative quotations

"I mean I think there is good engagement in the service at a senior level, and for that reason I think that focus, that sort of filters down across other members of the team." Kate, consultant geriatrician, Springhill

For peer review only - http://bmjopen.bmj.com/site/about/guidelines.xhtml

Multidisciplinary teamwork in hip fracture care

"I think if you get a core of people who are interested and enthusiastic, and I think that's across all disciplines, then there's no doubt that commitment can then drive improvements in other areas." Jon, consultant anaesthetist, Radford

"You've got to find key people [from a range of specialties] that are passionate about [hip fracture care] and get it. And even understand that, you know, the frailest of the frail, and you've got to do it properly and that takes lots of different services and lots of different professionals to pull that together … I guess I've been lucky here because people have put their head above the parapet rather than me pulling them in sort of kicking and screaming." Amanda, consultant orthogeriatrician, Springhill

"So, I think what worked well initially was a lot of buy-in and support from management. So three, four years ago one of the senior business unit managers, called in a couple of consultants that said, 'I'll support whatever you want to help this pathway better, what can we do about it?'… I think [it's helpful because] it brings the whole team together." Peter, consultant orthopaedic surgeon, Radford

Discussion

This study has characterised how hospitals deliver effective multidisciplinary work for hip fracture care and used findings to develop a model with core components needed to deliver safe and effective multidisciplinary team care. Findings identified five components to promote effective multidisciplinary teamwork including: (i) defined roles and

For peer review only - http://bmjopen.bmj.com/site/about/guidelines.xhtml

Multidisciplinary teamwork in hip fracture care

responsibilities, (ii) information transfer processes, (iii) shared goals, (iv) shared responsibility, and (v) collaborative leadership.

Relationship to current literature

This study complements existing guidelines on optimal care models for hip fracture care, by exploring how multidisciplinary teamwork can be organised and delivered in practice. Guidelines and existing research have highlighted the importance of implementing formal processes to coordinate care, including use of well-defined care pathways and protocols[5, 37] and good communication to facilitate information transfer[14]. The importance of shared responsibility through care models between orthogeriatricians and orthopaedic surgeons[38, 39] and formal coordinating roles such as trauma and discharge coordinators, have been widely acknowledged[40, 41]. NICE implementation advice for hip fracture management and British Orthopaedic Association (BOA) guidelines have both highlighted the importance of identifying an overarching clinical lead for hip fracture care, supported by senior leads from a range of specialties[14, 42]. The current study, which highlighted the need for this approach, identified the importance of informal, spontaneous communication strategies in facilitating information exchange. This includes use of technologies such as email, WhatsApp and mobile 'phones (but not commonly used pagers). Crucially, the study has further identified the importance of shared goals and a culture of shared responsibility for care delivery. It highlights how care processes such as multidisciplinary team meetings, monitoring and auditing processes and multidisciplinary team training can serve dual purposes by creating and sustaining these values.

For peer review only - http://bmjopen.bmj.com/site/about/guidelines.xhtml

Multidisciplinary teamwork in hip fracture care

Findings reflect wider systemic issues that have been identified in the context of other multidisciplinary teams within the NHS. These include the existence of structural arrangements within and between secondary care and external organisations that hinder communication, such as incompatible computing systems[43], staff transience[17] and steep hierarchies that can stymie communication within teams [44]. The socialisation of staff into different professional groups with distinct cultures, identities and priorities has further been identified as a hindrance to collaborative work[45]. The model of clinical service leadership described here reflects a broader trend in healthcare away from a 'command and control' approach to a motivational and facilitative style that involves building relationships with staff from a range of specialties[46]. It also reflects an increasing recognition of the value of collaborative leadership in which leadership roles are shared between several team members[47]. Our findings also highlight the importance of developing collaborative leadership across several different teams.

Models of care for hip fracture identified in this study are multidisciplinary whereby multiple specialties have separate roles, with some evidence of interdisciplinary working through blurring of traditional role boundaries[19]. Core components of teamwork in this context encompass a range of skills (*e.g.,* leadership), processes (*e.g.*, hip fracture protocols and pathways, formal mechanisms for information transfer) and values (*e.g.*, shared goals)[17] and reflect those in existing theories and frameworks. Several strategies to support teamwork are reflected in previous work, including the use of interdisciplinary training[48], shared monitoring and evaluation processes[17] and familiarisation with colleagues to build trust[21]. Our work contributes to existing literature, particularly by developing a new

For peer review only - http://bmjopen.bmj.com/site/about/guidelines.xhtml

Multidisciplinary teamwork in hip fracture care

concept of *shared responsibility* in facilitating collaborative practice. This finding may be relevant to other multidisciplinary teams delivering similar interventions.

This work complements ongoing quantitative research to understand the relative importance of systems and individual factors on patient care and outcomes. This multi-methods programme will develop a toolkit to enable service providers and commissioners to improve the organisation and delivery of hip fracture services[27], results due in 2023. Based on findings, we have developed our model into practical recommendations to improve teamworking competencies in hip fracture care, such as interdisciplinary 'exchange' training built into both orthopaedic and orthogeriatrics speciality training curricula (Supplementary material). An approach successfully achieved for other conditions that require multidisciplinary teamwork including cancer care[22, 49].

Supplementary material: Multidisciplinary toolkit hip fracture care

Strengths and limitations

The sample, comprising 40 participants drawn from four UK hospitals, provided sufficient data to address the research question and included professionals with relevant experience in hip fracture care, providing information power[30]. Between 5–15 interviews were conducted at each study site. We invited all healthcare professionals who were identified as working within the service. It is possible that those who declined were less engaged in service delivery. However, we are confident that we collected a range of views and experiences. Data collection was conducted in hospitals that admitted moderate to large

For peer review only - http://bmjopen.bmj.com/site/about/guidelines.xhtml

numbers of hip fracture patients. This may limit transferability of findings to smaller units[50].

Existing theories and frameworks around effective teamwork informed key aspects of teamworking for hip fracture care, providing confidence that the resulting model was theoretically informed. Using an abductive analysis approach, rather than transposing data onto an existing theory or framework, ensured the model was data-driven and that data were not 'forced' into pre-defined constructs[34]. A challenge was that data could be coded into more than one concept, for instance multidisciplinary team meetings and multidisciplinary team training were seen as facilitating several aspects of teamwork. Where relevant, data were therefore coded into more than one concept.

Further research

Further work is needed to understand leadership configurations in hip fracture care, particularly the role of 'emergent' leaders who do not have formal roles but exert considerable influence within teams[51]. Future research is planned to evaluate the impact of our toolkit on the organisation and delivery of hip fracture services.

Conclusions

This study used qualitative methods to develop a model of core components needed to deliver multidisciplinary teamwork for hip fracture care. It highlights the importance of creating and maintaining common goals and a culture that includes shared responsibility for

For peer review only - http://bmjopen.bmj.com/site/about/guidelines.xhtml

Multidisciplinary teamwork in hip fracture care

care delivery. These findings may be relevant to the delivery of hip fracture care outside of the UK and for patients with other conditions that require multidisciplinary care.

Acknowledgements

We would like to thank all the healthcare professionals who participated in this study and the REDUCE study group who provided support and guidance for this work. The REDUCE study group consists of Dr Rita Patel, Professor Andrew Judge, Professor Antony Johansen, Dr Elsa M R Marques, Dr Petra Baji, Ms Jill Griffin, Ms Marianne Bradshaw, Dr Katie Whale, Mr Tim Chesser, Professor Xavier L Griffin, Dr Muhammad K Javaid and Professor Yoav Ben-Shlomo.

Author contributions

All authors contributed to the design of this research and contributed to the acquisition, analysis and/or interpretation of data. All authors contributed to drafting this work and revising it for important intellectual content, and all gave final approval for the version to be submitted.

Competing interests

Authors have no competing interests to declare.

For peer review only

For peer review only - http://bmjopen.bmj.com/site/about/guidelines.xhtml

Multidisciplinary teamwork in hip fracture care

Funding

This work was supported by Versus Arthritis (reference 22086).

Data sharing statement

Anonymised interview data will be accessed via the University of Bristol Research Data Repository. Access to the data will be made available to researchers for ethically approved research projects, from a six-year embargo period, which ends on 1st April 2029. Data can be accessed on the understanding that confidentiality will be maintained and after a Data Access Agreement has been signed by an institutional signatory. No authentic request for access will be refused. Data are available at the University of Bristol data repository, data.bris, at https://doi.org/10.5523/bris.3f9j9z626s7f32a3r5j1wd7zt7.

Ethics approval

Ethical approval was provided by the Faculty of Health Sciences University of Bristol Research Ethics Committee (Ref: 108284) and by the NHS Health Research Authority (20/HRA/71). NHS settings (Trusts) for the four study sites each provided research governance approval.

References

1. Gullberg, B., O. Johnell, and J.A. Kanis, *World-wide projections for hip fracture.* Osteoporos Int, 1997. **7**(5): p. 407-13.

For peer review only - http://bmjopen.bmj.com/site/about/guidelines.xhtml

Multidisciplinary teamwork in hip fracture care

2. Neuburger, J., et al., *The Impact of a National Clinician-led Audit Initiative on Care and Mortality after Hip Fracture in England: An External Evaluation using Time Trends in Non-audit Data.* Medical Care, 2015. **53**(8): p. 686-691.

3. Griffin, X.L., et al., *Recovery of health-related quality of life in a United Kingdom hip fracture population. The Warwick Hip Trauma Evaluation--a prospective cohort study.* Bone Joint J,, 2015. **97-b**(3): p. 372-82.

4. Judge A, et al., *Models of care for the delivery of secondary fracture prevention after hip fracture: a health service cost, clinical outcomes and cost-effectiveness study within the South Central Region. National Institute for Health Research (NIHR), Health Services and Delivery Research (HS&DR)*. 2016.

5. Kates, S.L., *Hip fracture programs: are they effective?* Injury, 2016. **47 Suppl 1**: p. S25-7.

6. Zidén, L., M.H. Scherman, and C.G. Wenestam, *The break remains – elderly people's experiences of a hip fracture 1 year after discharge.* Disabil Rehabil, 2010. **32**(2): p. 103-113.

7. Bruun-Olsen, V., A. Bergland, and K.E. Heiberg, *"I struggle to count my blessings": recovery after hip fracture from the patients' perspective.* BMC Geriatrics, 2018. **18**(1): p. 18.

8. McMillan, L., et al., *A grounded theory of taking control after fall-induced hip fracture.* Disabil Rehabil, 2012. **34**(26): p. 2234-41.

9. National Institute for Health and Care Excellence (NICE), *Hip fracture in adults: Quality standard [QS16]*. 2017, Manchester: NICE.

10. National Institute for Health and Care Excellence (NICE), *Hip fracture: management: Clinical guideline [CG124]*. 2017: Manchester, NICE.

11. National Osteoporosis Society, *Effective secondary prevention of fragility fractures: clinical standards for fracture liaison services*. 2015: Bath: NOS.

12. Association, B.O., *BOAST 1 version 2-patients sustaining a fragility hip fracture.* London: British Orthopaedic Association, 2012.

For peer review only

For peer review only - http://bmjopen.bmj.com/site/about/guidelines.xhtml

Multidisciplinary teamwork in hip fracture care

13. Falls and Fragility Fracture Audit Programme (FFFAP), *National Hip Fracture Database (NHFD) annual report*. 2021: Royal College of Physcican's, London.

14. *The care of patients with fragility fracture*. 2007: British Orthopaedic Association.

15. Willcocks, S.G., *Exploring team working and shared leadership in multi-disciplinary cancer care.* Leadersh Health Serv (Bradf Engl), 2018. **31**(1): p. 98-109.

16. Grand, J.H., S. Caspar, and S.W. Macdonald, *Clinical features and multidisciplinary approaches to dementia care.* Journal of multidisciplinary healthcare, 2011. **4**: p. 125-147.

17. Ellis, G. and N. Sevdalis, *Understanding and improving multidisciplinary team working in geriatric medicine.* Age and Ageing, 2019. **48**(4): p. 498-505.

18. Salas, E., D.E. Sims, and C.S. Burke, *Is there a "Big Five" in Teamwork?* Small Group Research, 2005. **36**(5): p. 555-599.

19. D'Amour, D., et al., *The conceptual basis for interprofessional collaboration: core concepts and theoretical frameworks.* J Interprof Care, 2005. **19 Suppl 1**: p. 116-31.

20. Lemieux-Charles, L. and W.L. McGuire, *What Do We Know about Health Care Team Effectiveness? A Review of the Literature.* Medical Care Research and Review, 2006. **63**(3): p. 263-300.

21. D'Amour, D., et al., *A model and typology of collaboration between professionals in healthcare organizations.* BMC Health Serv Res, 2008. **8**: p. 188.

22. Rosen, M.A., et al., *Teamwork in healthcare: Key discoveries enabling safer, high-quality care.* The American psychologist, 2018. **73**(4): p. 433-450.

23. Soukup, T., et al., *Successful strategies in implementing a multidisciplinary team working in the care of patients with cancer: an overview and synthesis of the available literature.* J Multidiscip Healthc, 2018. **11**: p. 49-61.

24. Salas, E., D.L. Reyes, and S.H. McDaniel, *The science of teamwork: Progress, reflections, and the road ahead.* Am Psychol, 2018. **73**(4): p. 593-600.

For peer review only - http://bmjopen.bmj.com/site/about/guidelines.xhtml

Multidisciplinary teamwork in hip fracture care

25. Bélanger, E. and C. Rodríguez, *More than the sum of its parts? A qualitative research synthesis on multi-disciplinary primary care teams.* J Interprof Care, 2008. **22**(6): p. 587-97.

26. Coyne, I.T., *Sampling in qualitative research. Purposeful and theoretical sampling; merging or clear boundaries?* J Adv Nurs, 1997. **26**(3): p. 623-30.

27. Patel, R., et al., *REducing unwarranted variation in the Delivery of high qUality hip fraCture services in England and Wales (REDUCE): protocol for a mixed-methods study.* BMJ Open, 2021. **11**(5): p. e049763.

28. Baker, S.E. and R. Edwards, *How many qualitative interviews is enough.* 2012.

29. *Snowball sampling*, in *The SAGE encyclopedia of social science research methods*, M.S. Lewis-Beck, Bryman, A., & Futing Liao, T. , Editor. 2004, Sage Publications, Inc.: Thousand Oaks, CA.

30. Malterud, K., V.D. Siersma, and A.D. Guassora, *Sample Size in Qualitative Interview Studies: Guided by Information Power.* Qual Health Res, 2016. **26**(13): p. 1753-1760.

31. *Qualitative Researching with Text, Image and Sound*. 2000: London.

32. Tavory, I. and S. Timmermans, *Abductive analysis: Theorizing qualitative research*. 2014: University of Chicago Press.

33. Nilsen, P., *Making sense of implementation theories, models and frameworks.* Implementation Science, 2015. **10**(1): p. 53.

34. Edwards-Jones, A., *Qualitative data analysis with NVIVO*. 2014, Taylor & Francis.

35. Pope, C., S. Ziebland, and N. Mays, *Qualitative research in health care. Analysing qualitative data.* Bmj, 2000. **320**(7227): p. 114-6.

36. Gooberman-Hill, R., et al., *Involving patients in research: considering good practice.* Musculoskeletal Care, 2013. **11**(4): p. 187-90.

37. Meinberg, E., et al., *Hospital-based Hip fracture programs: Clinical need and effectiveness.* Injury, 2020. **51**: p. S2-S4.

38. Fernandez, M.A., X.L. Griffin, and M.L. Costa, *Management of hip fracture.* British Medical Bulletin, 2015. **115**(1): p. 165-172.

For peer review only - http://bmjopen.bmj.com/site/about/guidelines.xhtml

Multidisciplinary teamwork in hip fracture care

39. Chudyk, A.M., et al., *Systematic Review of Hip Fracture Rehabilitation Practices in the Elderly.* Archives of Physical Medicine and Rehabilitation, 2009. **90**(2): p. 246-262.

40. Riemen, A.H.K. and J.D. Hutchison, *The multidisciplinary management of hip fractures in older patients.* Orthopaedics and trauma, 2016. **30**(2): p. 117-122.

41. (UK), N.I.f.H.a.C.E., *Major Trauma: Service Delivery NICE Guideline NG40*. 2016.

42. (NICE), N.I.f.H.a.C.E., *Implementation advice; Implementing the Hip Fracture Programme*. 2012, NICE: Manchester.

43. Zhang, J., et al., *Interoperability in NHS hospitals must be improved: the Care Quality Commission should be a key actor in this process.* Journal of the Royal Society of Medicine, 2020. **113**(3): p. 101-104.

44. Green, B., et al., *Challenging hierarchy in healthcare teams – ways to flatten gradients to improve teamwork and patient care.* British Journal of Oral and Maxillofacial Surgery, 2017. **55**(5): p. 449-453.

45. Powell, A.E. and H.T.O. Davies, *The struggle to improve patient care in the face of professional boundaries.* Social Science & Medicine, 2012. **75**(5): p. 807-814.

46. Storey, J. and R. Holti, *Towards a New Model of Leadership for the NHS.* 2013.

47. Günzel-Jensen, F., A.K. Jain, and A.M. Kjeldsen, *Distributed leadership in health care: The role of formal leadership styles and organizational efficacy.* Leadership, 2016. **14**(1): p. 110-133.

48. Reeves, S., et al., *Interprofessional education: effects on professional practice and healthcare outcomes.* Cochrane Database of Systematic Reviews, 2013(3).

49. *The Characteristics of an Effective Multidisciplinary Team (MDT)*. 2010; Available from: file:///C:/Users/sd17432/Downloads/NCATMDTCharacteristics_FINAL.pdf.

50. Forero, R., et al., *Application of four-dimension criteria to assess rigour of qualitative research in emergency medicine.* BMC Health Services Research, 2018. **18**(1): p. 120.

51. Hanna, A.A., et al., *The Emergence of Emergent Leadership: A Comprehensive Framework and Directions for Future Research.* Journal of Management, 2020. **47**(1): p. 76-104.

For peer review only - http://bmjopen.bmj.com/site/about/guidelines.xhtml

Multidisciplinary teamwork in hip fracture care

For peer review only - http://bmjopen.bmj.com/site/about/guidelines.xhtml

Figure 1: Core components of effective multidisciplinary teamwork for hip fracture care

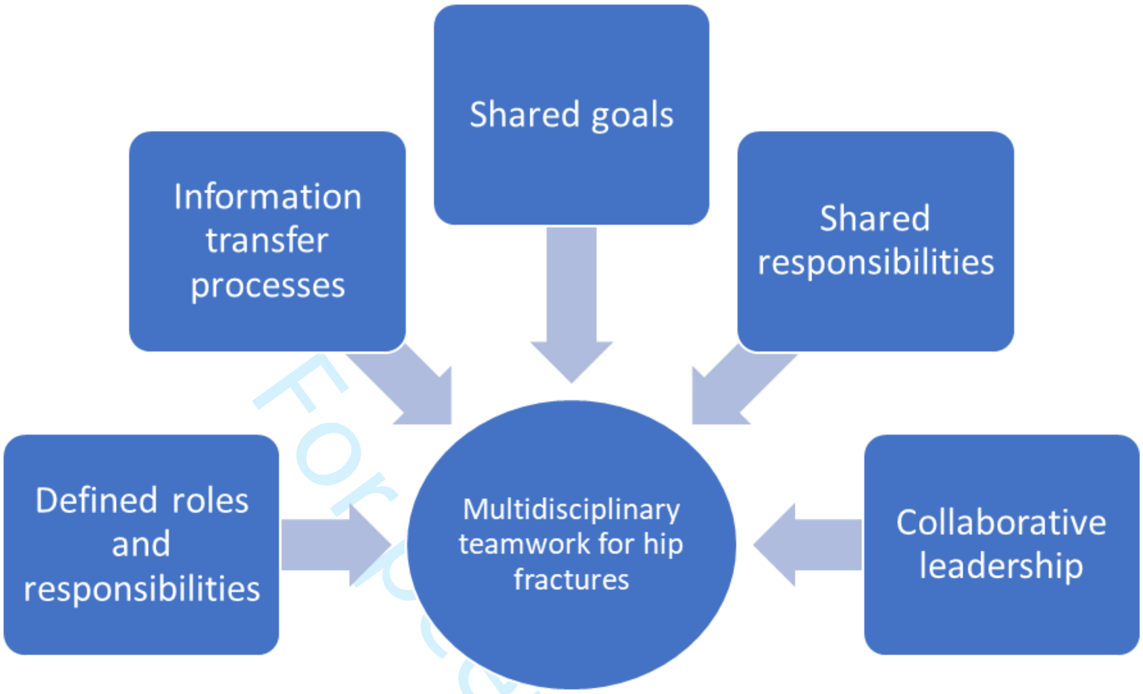

Figure 1: Topic guide

REDUCE: A study of service delivery for hip fracture care

Aims and objectives

- To investigate how hip fracture services are provided in different hospital settings and factors that help/ hinder ability to deliver care
- This study will provide us with information that can be used to improve the delivery of hip fracture services and reduce variation in care

Topics covered will be:

- Find out a bit about you
- Organisation of the care pathway
- Barriers and facilitators to service delivery
- Close

Part 1: About you

- Job title
- Part time/ full time/ %FTE
- Professional background (years since qualifying and progression)
- Years since qualifying for this role
- Years spent working in this service
- Age
- Date of birth
- Gender identify with
- Describe own role in delivering hip fracture care (before Covid)
  - Face to face duties
  - Administrative / organisational duties
  - Management duties

review only

Part 2: Care pathway before Covid-19

- Things that worked well/ not so well/ improvements.

  Examples of components of care

  - Ambulance hand over
  - Emergency department factors [NB: Fast-tracking to ward]
  - Imaging services
  - Orthogeriatric services
  - Pre-operative care
  - Theatre and anaesthetic services
  - Orthopaedic & Trauma Services
  - Inpatient physiotherapy services and rehabilitation
  - Training/ confidence in the manual handling of patients
  - Links to dementia care, mental health services, diabetes care, dietetics, palliative care
  - Discharge systems and links to intermediate care
  - Temporary rehabilitation / care home provision
  - Post-discharge queries from patients
  - Re-admission processes for potential complications
  - Fracture Liaison Services/ secondary fracture prevention pathways
  - Falls services
  - Patient information and education
  - Community physiotherapy services and rehabilitation
  - Relationships with care homes – infection control protocols for new placements and return of patients to their original care homes

For peer review only

Part 3: Barriers and facilitators to service delivery before COVID-19

Things that worked well/ not so well/ improvements:

Communication

- How well did everyone communicate within service?
    - Within team
    - Other colleagues in MDT
    - Links with other services, e.g. dementia care, mental health services, diabetes care, palliative care
    - Community services and rehabilitation

  [Probes: MDT/ morbidity and mortality meetings, trauma rounds, multi-disciplinary paperwork, shared protocols, shared governance meetings, central coordinator]

  - What worked well/ not so well/ improvements
- Department management/ senior management
  - What worked well/ not so well/ improvements
- External organisations
    - Primary care
    - Community services and rehabilitation
    - Social care services

Relationships

- What does a 'good' working relationship mean to you?
- How well did everyone work together
    - Within team
    - Other colleagues in MDT

For peer review only

- Department management/ senior management

- What sorts of things contributed to good working relationship with colleagues/ made it more difficult?

- Improvements

Commitment to delivering care

- To what extent did your colleagues do what was expected of them?

  [Probes: Extent to which the 36 hour target for hip fracture operation was pushed, extent individuals adhered to protocols]

  - Why do you think this was?

- How engaged/ enthusiastic do you think your colleagues were?

  - What helped/ made it more difficult?

Access to resources

- To what extent did you have access to all the resources needed to carry out work?

  [Probes: staff vacancies, theatre capacity, surgeon capacity, workload and material resources, access to medical and social care data],

  If not, why not?

  What did you do to address this challenge?

- To what extent did you/ team have access to the training and information needed to carry out work?

  [Probes: training, evidence base, understanding what is required of them]

- Training/ mentorship programmes in place

  - What worked well/ not so well/ improvements

- How much of a priority do you feel that hip fracture care was for the?

  - Hospital Trust

  - Department

  - Boss

For peer review only

- Why/ why not priority?
- Strategies to engage senior management

Service delivery over time

- Example quality improvement project
  - How did change occur?
- Example when you tried to initiate change but couldn't
  - Why?
- What monitoring and evaluation processes are in place?

  [Probes: national initiatives, internal audit and governance processes, review of cases in morbidity and mortality meetings]
- What is the impact of these monitoring and evaluation processes on service delivery?

  [Probes: Are these fed back to staff, do they effect change]
- Example of local clinical audit – why initiated?
- What clinical governance activities are in place for hip fracture care?

  [Probes: risk management, transparency, quality improvement, mortality reviews, strategies to review clinical incidents/ complaints]

  How useful are they?

Part 4: Close

1. If you could change one thing about your (pre-Covid) service, what would it be?
2. Are there any other things or insights that you would like to tell me about today?
3. Do you have any questions for me about the project?
4. Thank them
5. Reaffirm confidentiality

For peer review only

A 'how to' guide for effective multi-disciplinary team working in hip fracture care

Delivering hip fracture care requires effective multi-disciplinary teamwork, necessitating effective communication and coordination across a range of specialties and professional roles including emergency medicine, orthopaedic surgery, anaesthetics, geriatric medicine, rehabilitation and nurses. The range of the multidisciplinary team involved reflects the complex needs of patients with hip fracture. Clinical guidelines recommend timely hip fracture care, with when needed, rapid transfers of care between professionals. Streamlining multi-disciplinary care is crucial to the delivery of safe and effective patient care.

There are five components needed for effective, coordinated multi-disciplinary team (MDT) working. The responsibility for delivering these components lies with *all members* of the MDT.

Action 1: Clearly define roles and responsibilities of each member of the MDT

Roles and responsibilities are more than what is simply listed in a job description. Each member of the MDT needs to understand the other MDT member's roles and responsibilities. Hence, roles and responsibilities need to be appreciated and explained during induction processes.

Roles and responsibilities need to be co-agreed between an individuals' line manager, within the MDT and outlined in personalized job plans. These job plans should be reviewed at annual appraisals with modifications made according to MDT developments/ progress.

The clinical lead needs to have oversight of all roles and responsibilities in order to identify gaps in the pathway of hip fracture care, so as to manage the skillset within the MDT to ensure those gaps are filled.

Action 2: Set up systems to ensure clear, timely MDT communication

Hip fracture patients have complex medical and surgical needs and are often acutely unstable. Therefore, MDT communication must be dynamic and efficient with the capacity for immediate response from senior team members when necessary. Systems are enhanced by the presence of trauma coordinators and discharge coordinators with defined roles and responsibilities.

Face-to-face communication events (verbal)
Systems need to be established for effective communication events with those attending pre-agreed. Communication events should begin promptly, using the same standardised format each time. Job plans need to permit attendance. Examples of communication events include daily trauma meetings, early morning board rounds (aka 'huddles') and pre-arranged discharge planning meetings. Short meetings are more efficiently conducted whilst standing, where ability permits.

Six principles of effective MDT communication events include:

1. Ensure representation from the key disciplines
2. Establish patient centred goals
3. Bring opinions together openly
4. Agree an overall aim, next steps with time frames
5. Allocate responsibilities

For peer review only - http://bmjopen.bmj.com/site/about/guidelines.xhtml

6. Communicate/ document outputs

Written MDT communication
Written MDT documentation should be appropriately co-designed to capture all relevant information. All patient admissions should be clerked using a standardised proforma co-agreed and designed by the MDT with sections addressing trauma at other skeletal sites (besides the hip), nerve block use, osteoporosis management and orthogeriatric and orthopaedic senior reviews. Documents need to be available to the wider MDT.
Examples of documents/ proformas that can assist the delivery of care include:

- Structured admission clerking proforma
- Structured post-operative junior doctor review (for patients returning to the ward from theatre)
- Structured falls and bone health assessments

Examples of documents are available on the National Hip Fracture Database (NHFD): https://www.rcplondon.ac.uk/projects/outputs/national-hip-fracture-database-nhfd-improvement-repository

Electronic MDT communication
Smart Phones have made radio pagers redundant. All staff should have access to a Smart Phone. Information can be managed securely through e.g. Siilo for patient specific information. WhatsApp can be used for team communication, but not patient specific information. Mobile phone numbers should be routinely shared within the MDT. Appropriate groups should be set-up.

Hospitals need to invest in electronic communication systems e.g. (i) to enable trauma wards to have live monitoring of theatre activity, to plan prompt transfers to and from theatre, and (ii) routine access to primary care records. Templated discharge summaries, e.g. with use of standardised phrases, can improve efficiency and increase communication at discharge.

Hip fracture patients are best managed on a hip fracture ward where all members of the MDT can be co-located and thus easily found for spontaneous communication, with nearby office space. Timetabling of consultant ward rounds and daily ward activities should be shared amongst the whole team and adhered to.

Action 3: Establish inter-disciplinary principles of working

Healthcare teams can be 'multidisciplinary ' or 'interdisciplinary'. Multi-disciplinary teams have separate roles and work within their own specific disciplines. Interdisciplinary teams work more closely together to deliver shared goals and some roles may overlap.

Where human resource permits, hip fracture care should be provided through a shared care model between orthopaedic and orthogeriatric services.

Orthopaedic, geriatric and anaesthetic specialist trainees should receive formal *inter-disciplinary* training to ensure mutual understanding of each other's roles, e.g. geriatric trainees should attend a trauma meeting and a hip fracture theatre session, whilst orthopaedic trainees should join an orthogeriatric ward round and geriatrician lead white board rounds. Similarly, an orthogeriatric trainee should spend time with an orthopaedic physiotherapist whilst the anaesthetic trainee should

For peer review only - http://bmjopen.bmj.com/site/about/guidelines.xhtml

For peer review only

join the orthogeriatric ward round. The ultimate goal of interdisciplinary training is to ensure mutual understanding and professional support between roles.

All trainees should attend clinical governance meetings to understand governance structured necessary to support a complex pathway of patient care.

Examples of *inter-disciplinary* upskilling include:

- Nurses should be upskilled by physiotherapists to confidently mobilise patients post-operatively
- Emergency department doctors should be upskilled by anaesthetists to provide emergency nerve blocks
- Physiotherapists should be upskilled by occupational therapists to assist discharge planning
- Orthopaedic trainees should be upskilled by orthogeriatricians to assess cognitive capacity to consent

Agree cross-cover arrangements within clinical competencies, e.g. physiotherapy and occupational therapy co-working, medical and surgical junior doctors cross-covering, nursing staff empowered to mobilise patients.

Action 4: Establish collaborative MDT team leadership

Orthogeriatric (1), orthopaedic (2), anaesthetic (3) consultant hip fracture leads should have their role defined within their job plan. The orthopaedic and/or orthogeriatric consultant should identify as clinical (co-)lead for hip fracture services.

Strong clinical leadership is essential to co-ordinate effective MDT hip fracture care delivery. Formal leadership training is strongly encouraged.

A physiotherapy (4), nursing (5), occupational therapy (6) and pharmacy (7) discipline lead for hip fracture care should be appointed.

All seven of these professionals have responsibility to attend clinical governance meetings and act as conduit for feedback from and to their individual specialist teams.

All disciplinary leads should consider succession planning and discuss this with the leadership team to manage leadership transitions smoothly and efficiently (no discipline should be without hip fracture leadership). Aim for early identification of successors with structured mentorship with staged handover.

Identify a hospital board executive responsible for bidirectional communication with the hip fracture clinical lead, who will champion hip fracture care at board level. All seven discipline-specific hip fracture leads should have met with the hospital board executive. The primary point of contact for the hospital board executive should be the hip fracture service clinical lead.

For peer review only

For peer review only - http://bmjopen.bmj.com/site/about/guidelines.xhtml

Action 5: Jointly agree the goals of the team and strategies to achieve these goals

Define MDT goals by jointly reviewing NHFD performance, bench-marked KPIs, and how these compare against national averages, as well as REDUCE toolkit targets and any specialty-specific internal audit/ QI data. It's really important to get all members of the team in the room and to hear the voices and perspectives of each MDT discipline with no one specialty dominating the conversation. The clinical lead for hip fracture services holds the responsibility for coordinating this process.

Strategies to improve services should be structured into short, medium and long-term approaches and achievements routinely reviewed at clinical governance and morbidity and mortality (M&M) meetings with plans and progress clearly communicated to the hip fracture service hospital board executive. The REDUCE toolkit is designed to facilitate each hospital to develop their own individualized strategies towards service improvement.

For peer review only - http://bmjopen.bmj.com/site/about/guidelines.xhtml

For peer review only

Consolidated criteria for reporting qualitative studies (COREQ): 32-item checklist

Please indicate in which section each item has been reported in your manuscript. If you do not feel an item applies to your manuscript, please enter N/A.

For further information about the COREQ guidelines, please see Tong *et al.*, 2017: https://doi.org/10.1093/intqhc/mzm042

**No.**	**Item**	**Description**	**Section #**
**Domain 1: Research team and reflexivity**			
Personal characteristics			
1.	Interviewer/facilitator	Which author/s conducted the interview or focus group?	
2.	Credentials	What were the researcher's credentials? *E.g. PhD, MD*	
3.	Occupation	What was their occupation at the time of the study?	
4.	Gender	Was the researcher male or female?	
5.	Experience and training	What experience or training did the researcher have?	
Relationship with participants			
6.	Relationship established	Was a relationship established prior to study commencement?	
7.	Participant knowledge of the interviewer	What did the participants know about the researcher? *E.g. Personal goals, reasons for doing the research*	
8.	Interviewer characteristics	What characteristics were reported about the interviewer/facilitator? *E.g. Bias, assumptions, reasons and interests in the research topic*	
**Domain 2: Study design**			
Theoretical framework			
9.	Methodological orientation and theory	What methodological orientation was stated to underpin the study? *E.g. grounded theory, discourse analysis, ethnography, phenomenology, content analysis*	
Participant selection			
10.	Sampling	How were participants selected? *E.g. purposive, convenience, consecutive, snowball*	
11.	Method of approach	How were participants approached? *E.g. face-to-face, telephone, mail, email*	
12.	Sample size	How many participants were in the study?	
13.	Non-participation	How many people refused to participate or dropped out? What were the reasons for this?	
Setting			
14.	Setting of data collection	Where was the data collected? *E.g. home, clinic, workplace*	
15.	Presence of non-participants	Was anyone else present besides the participants and researchers?	

For peer review only

16.	Description of sample	What are the important characteristics of the sample? *E.g. demographic data, date*	
Data collection			
17.	Interview guide	Were questions, prompts, guides provided by the authors? Was it pilot tested?	
18.	Repeat interviews	Were repeat interviews carried out? If yes, how many?	
19.	Audio/visual recording	Did the research use audio or visual recording to collect the data?	
20.	Field notes	Were field notes made during and/or after the interview or focus group?	
21.	Duration	What was the duration of the interviews or focus group?	
22.	Data saturation	Was data saturation discussed?	
23.	Transcripts returned	Were transcripts returned to participants for comment and/or correction?	
**Domain 3: analysis and findings**			
Data analysis			
24.	Number of data coders	How many data coders coded the data?	
25.	Description of the coding tree	Did authors provide a description of the coding tree?	
26.	Derivation of themes	Were themes identified in advance or derived from the data?	
27.	Software	What software, if applicable, was used to manage the data?	
28.	Participant checking	Did participants provide feedback on the findings?	
Reporting			
29.	Quotations presented	Were participant quotations presented to illustrate the themes / findings? Was each quotation identified? *E.g. Participant number*	
30.	Data and findings consistent	Was there consistency between the data presented and the findings?	
31.	Clarity of major themes	Were major themes clearly presented in the findings?	
32.	Clarity of minor themes	Is there a description of diverse cases or discussion of minor themes?	

When submitting your manuscript via the online submission form, please upload the completed checklist as a Figure/supplementary file.

If you would like this checklist to be included alongside your article, we ask that you upload the completed checklist to an online repository and include the guideline type, name of the repository, DOI and license in the *Data availability* section of your manuscript.

Developed from: Allison Tong, Peter Sainsbury, Jonathan Craig, Consolidated criteria for reporting qualitative research (COREQ): a 32-item checklist for interviews and focus groups, International Journal for Quality in Health Care, Volume 19, Issue 6, December 2007, Pages 349–357, https://doi.org/10.1093/intqhc/mzm042

For peer review only

BMJ Open

A model of multidisciplinary teamwork in hip fracture care: a qualitative interview study

Journal:	*BMJ Open*
Manuscript ID	bmjopen-2022-070050.R1
Article Type:	Original research
Date Submitted by the Author:	31-Jul-2023
Complete List of Authors:	Drew, Sarah; University of Bristol, Translational Health Sciences<br>Fox, Fiona; University of Bristol, Bristol Medical School (PHS)<br>Gregson, Celia; University of Bristol, Musculoskeletal Research Unit; Royal United Hospital Bath NHS Trust, Older Person's Unit<br>Gooberman-Hill, Rachael; University of Bristol, Bristol Medical School
**Primary Subject Heading**:	Health services research
Secondary Subject Heading:	Qualitative research
Keywords:	Organisation of health services < HEALTH SERVICES ADMINISTRATION & MANAGEMENT, Hip < ORTHOPAEDIC & TRAUMA SURGERY, QUALITATIVE RESEARCH

SCHOLARONE™
Manuscripts

I, the Submitting Author has the right to grant and does grant on behalf of all authors of the Work (as defined in the below author licence), an exclusive licence and/or a non-exclusive licence for contributions from authors who are: i) UK Crown employees; ii) where BMJ has agreed a CC-BY licence shall apply, and/or iii) in accordance with the terms applicable for US Federal Government officers or employees acting as part of their official duties; on a worldwide, perpetual, irrevocable, royalty-free basis to BMJ Publishing Group Ltd ("BMJ") its licensees and where the relevant Journal is co-owned by BMJ to the co-owners of the Journal, to publish the Work in this journal and any other BMJ products and to exploit all rights, as set out in our licence.

The Submitting Author accepts and understands that any supply made under these terms is made by BMJ to the Submitting Author unless you are acting as an employee on behalf of your employer or a postgraduate student of an affiliated institution which is paying any applicable article publishing charge ("APC") for Open Access articles. Where the Submitting Author wishes to make the Work available on an Open Access basis (and intends to pay the relevant APC), the terms of reuse of such Open Access shall be governed by a Creative Commons licence – details of these licences and which Creative Commons licence will apply to this Work are set out in our licence referred to above.

Other than as permitted in any relevant BMJ Author's Self Archiving Policies, I confirm this Work has not been accepted for publication elsewhere, is not being considered for publication elsewhere and does not duplicate material already published. I confirm all authors consent to publication of this Work and authorise the granting of this licence.

For peer review only

Title: A model of multidisciplinary teamwork in hip fracture care: a qualitative interview study

Short title: Multidisciplinary teamwork in hip fracture care

Corresponding author

Dr Sarah Drew

Research Fellow

Musculoskeletal Research Unit

Bristol Medical School, University of Bristol

Learning and Research Building, Level 1

Southmead Hospital

BS10 5NB

Mobile: + 44 (0)7891149828

Email: sarah.drew@bristol.ac.uk

Sarah Drew[1], MA, DPhil

Fiona Fox[1], BSc, PhD

Celia L Gregson[1], B.Med.Sci., M.B.,B.S., MSc, FRCP, PhD

Rachael Gooberman-Hill[1,2], MA, PhD

Author affiliations

1. Musculoskeletal Research Unit, Translational Health Sciences, Bristol Medical School, University of Bristol, Bristol, UK.
2. UK National Institute for Health Research Bristol Biomedical Research Centre,

For peer review only

University Hospitals Bristol and Weston NHS Foundation Trust and the University of Bristol, Bristol, UK.

Word count 3998 (max 4000)

Keywords

Hip fractures, multidisciplinary teamwork, service delivery, qualitative

For peer review only

Abstract 300 words

Objectives

Hip fractures are common injuries in older age with high mortality requiring multidisciplinary clinical care. Despite guidance, there is considerable variation in hip fracture services and patient outcomes; furthermore, little is known about how successful multidisciplinary working can be enabled. This study aimed to characterise professionals' views about the core components of multidisciplinary teamwork in hip fracture care.

Design

The study comprised qualitative interviews with healthcare professionals delivering hip fracture care. Interviews were audio-recorded, transcribed and analysed abductively: material was coded inductively and grouped into higher level concepts informed by theories and frameworks relating to teamwork.

Setting

Four hospitals in England.

Participants

Participants were 40 healthcare professionals including orthopaedic surgeons, orthogeriatricians, physiotherapists and service managers.

Results

Results identified four components of successful multidisciplinary teamwork: 1) defined roles and responsibilities, 2) information transfer processes, 3) shared goals, and 4) collaborative leadership. These were underpinned by a single concept: shared responsibility, Defined roles and responsibilities were promoted through formal care pathways, reinforced through induction and training with clear job plans outlining tasks. Information transfer

For peer review only

processes facilitated timely information exchange to appropriate individuals. Well-defined, common purpose was hindered by complex inter-disciplinary professional relationships, particularly between orthogeriatric and orthopaedic staff, and encouraged through multidisciplinary team meetings and training. Clinical service leads were integral to bridging interdisciplinary boundaries. Mutual trust and respect were based on recognition of the value of different professional groups. Teamwork depended upon formal clinical leads with facilitative and motivational roles, and on hospital leadership that created an environment supporting collaboration. Shared responsibility for patients was encouraged by joint orthopaedic and orthogeriatric care models. Staff shared responsibility by assisting colleagues when needed.

Conclusions

Shared responsibility across the multidisciplinary team is fundamental to delivery of hip fracture care. Findings will inform development of clinical practice recommendations and training to build teamworking competencies.

Article summary

Strengths and limitations of this study

- This qualitative study identified an underpinning concept in core components of multidisciplinary teamwork in hip fracture care: shared responsibility.
- Qualitative study design and methods using an abductive approach provided an in-depth understanding of care processes from the viewpoints of involved professionals and enabled the research to be informed by existing frameworks about teamwork.
- The sample, comprising 40 participants drawn from four UK hospitals, and information power through inclusion of a range of professionals with relevant experience and with

For peer review only

sufficient data to address the research aim.

- Despite achieving information power, there are some professionals involved in healthcare and related discharge planning—such as social workers—who were not included in the study. This means that we were unable to include their views. However, the common thread of shared responsibility would be likely to be relevant and meaningful to all professions involved in hip fracture care.
- Data collection was conducted in hospitals that admitted moderate to large numbers of hip fracture patients. This may limit transferability of findings to smaller units.

Funding statement

This work was supported by Versus Arthritis (reference 22086).

Competing interests

All authors declare that they have no competing interests.

For peer review only

Introduction

Globally, hip fractures pose a major challenge to patients and healthcare systems, with 4.5 million such fractures expected to occur annually by 2050[1]. Hip fractures are most commonly sustained by older individuals and can be devastating with considerable impact on quality of life and a high risk of subsequent death[2, 3]. Provision of hip fracture care is costly to healthcare providers, incurring $5.96 billion in direct medical costs each year in the United States and £1.2 billion in the United Kingdom (UK)[4]. Comprehensive multidisciplinary hip fracture care pathways reduce postoperative complications and mortality[5]. To make recommendations about how to further enhance the quality care of care for people who have had hip fracture, there is a need to understand how healthcare professionals implement care pathways.

In the UK, thorough guidance has been published on the management of hip fracture[6-8]. Despite this, considerable variation exists in the organisation of care pathways[7, 9] and in patient outcomes[10]. Hip fracture care requires communication and coordination across a range of specialties and professional roles including emergency medicine, surgery, anaesthetics, geriatric medicine and rehabilitation[6, 7]. This multidisciplinary care reflects patients' complex needs that may include multiple pre-existing conditions[11]. Guidelines recommend prompt care, with rapid transfers of care between professionals[7, 11].

Studies about provision of acute rehabilitation for hip fracture with clinical leaders and physiotherapists have highlighted the importance of communication between professionals and patients in rehabilitation for hip fracture[12, 13]. Similarly, a recent interview-based

For peer review only

study by Guerra and colleagues with healthcare professionals explored acute rehabilitation and implementation[14]. The study found that communication and collaboration are central to provision of effective rehabilitation and that organisational constraints are seen as a barrier to such provision. Other studies have also explored how patients and carers engage with hip fracture care, and qualitative findings highlight the emotional and practical challenges associated with hip fracture[6-8]. Together, these studies highlight the importance of understanding the way in which care for hip fracture is organised and delivered.

High quality multidisciplinary care is crucial to delivery of safe and effective services. Multidisciplinary teamwork is necessary in delivery of clinical care for a number of health conditions and specialties, such as cancer[15], dementia[16] and in older people's services[17]. 'Teamwork' can be defined as 'a set of inter-related thoughts, actions, and feelings of each team member that are needed to function as a team and that combine to facilitate coordinated, adaptive performance and task objectives resulting in value-added outcomes'[18]. Collaborative teamwork exists on a continuum of professional autonomy and integrative working. Three broad models describe collaborative teamwork: 'multidisciplinary', whereby multiple specialties with separate roles work within their own disciplines; 'interdisciplinary' that involves working together to deliver shared goals with some blurring of disciplinary boundaries; and 'transdisciplinary' where professional roles are shared[19]. Research has highlighted how different tasks across clinical disciplines shape how teams are organised and work together[20].

Existing theories and frameworks guide professionals in the organisation and evaluation of

For peer review only

the processes used in multidisciplinary team communications, training and leadership. Some frameworks are generalisable across several areas[18, 20-22], others have been developed for specific clinical disciplines[23]. Frameworks have identified diverse factors that promote clinical teamwork, including a shared understanding of team goals[18, 19, 21], successful information exchange[18], effective leadership[17, 18, 21, 23] and adaptability of services to meet changing demands[18, 24, 25]. Ellis and Sevdalis build on frameworks to identify core technical and non-technical skills to facilitate effective multidisciplinary teamwork in geriatric medicine[17]. Non-technical skills include social, cognitive and personal resource skills. Strategic domains to enhance multidisciplinary working are skills (e.g., good leadership), processes (e.g., good documentation governance) and values (e.g., prioritising patient needs and respect for colleagues). Evidence from behavioural research into multidisciplinary teamwork suggests that if any of these three components is missing, teamwork will be unsuccessful[17].

Given the importance of multidisciplinary teams in hip fracture care and previous learning from other fields, we aimed to develop a model of the core components needed to deliver successful multidisciplinary teamwork in hip fracture care in England. Understanding views about successful teamwork in the hip fracture care pathway will help to provide visibility to core components and inform future work to support and enhance multidisciplinary care for hip fracture.

Methods

We used qualitative interviews to collect and interpret insights from health professionals

For peer review only

engaged in delivery of hip fracture care in hospitals in England.

Sample

Hospital sites

Healthcare professionals involved in the organisation and delivery of hip fracture were sampled from four National Health Service (NHS) hospitals across England. The NHS provides almost all hip fracture care in England, free to patients at the point of receipt.

Purposive sampling was used to identify study sites[26], with variation in geography, number of hip fracture presentations per year, service configuration and audited hip fracture outcomes[27]. Three hospitals were urban and one rural. One hospital admitted a large number (>75th percentile), and three moderate (between 25th-75th percentile) numbers of hip fracture patients annually. Hospitals are identified by pseudonyms to retain anonymity ('Springhill', 'Radford', 'Maplegrove' and 'Newbridge').

Participants

Forty interviews were conducted with healthcare professionals across the four hospitals. Staff were purposively sampled using a criterion approach to include healthcare professionals from across the care pathway, including orthopaedic surgeons, anaesthetists, orthogeriatricians, physiotherapists and occupational therapists, nurses, trauma coordinators and service managers[28].

For peer review only

Potential participants were identified by a designated contact working within the service at each hospital site. Staff were invited to participate in the study by email, followed-up with reminder emails a minimum of two weeks later. Snowball sampling was also used[29], with participants recommending other professionals involved in service delivery. In total, 75 healthcare professionals were approached to take part in the study, of whom, 40 (53%) agreed to take participate. The remainder either declined or were unavailable. The final sample enabled achievement of information power as it was adequate to address the study aim through inclusion of appropriate participants across the care pathway [30].

Ethical approval

Ethical approval was provided by the Faculty of Health Sciences University of Bristol Research Ethics Committee (Ref: 108284) and by the NHS Health Research Authority (20/HRA/71). Written informed consent was provided by all participants, confirming understanding of voluntary participation and potential publication of anonymised interview quotations. NHS settings (Trusts) for the four study sites each provided research governance approval.

Data collection

Two qualitative researchers (FF and SD) conducted interviews by telephone or teleconferencing technology (e.g., Microsoft teams), necessitated by Covid-19 pandemic restrictions. Interviews, lasting 45 to 90 minutes, were guided by a topic guide based on

For peer review only

domains agreed collaboratively with healthcare professionals involved in delivery and organisation of hip fracture services. Part 1 focused on barriers and facilitators to the implementation of care. Part 2 covered multidisciplinary communication and cooperation including questions about access to resources, monitoring and evaluation (Supplementary material). Interviews were flexible to explore emerging ideas[31] and questions relating to teamwork were covered in all domains.

Data analysis

Interviews were audio-recorded, transcribed and anonymised to remove any identifying information. Participants were assigned pseudonyms. Data were analysed using an abductive approach [32]. Abduction is an approach that involves using research findings to develop new theories that build upon existing theories and frameworks[33]. Further detailed explanation of abduction can be found in Tavory and Timmermans[32]. There are several stages to the abductive process. First, transcripts were imported into NVivo qualitative data management software[34] and coded using an inductive thematic approach[35]. In this process, we focused on aspects of the data relating to teamwork, which we found in all interviews. Next, codes and coded material was grouped into higher level concepts. In keeping with an abductive approach, the concepts were informed by existing frameworks about teamwork[17, 18, 21]. A model was then constructed to illustrate how core concepts contributed to multidisciplinary teamwork for hip fracture care (See Figure 1). During this process all transcripts were analysed in the same way with the same staged process applied. To ensure rigor, 40% transcripts were independently analysed in duplicate (by SD and FF) and themes reviewed and refined to reach an agreed code list[35].

For peer review only

Patient and public involvement

To refine the study design and data collection materials, we held three meetings with members of 'The Patient Experience Partnership in Research' (PEP-R) group, a dedicated musculoskeletal patient involvement group[36]. Members' input helped to shape study documentation, and they felt that findings resonated with their own experiences, particularly reflecting their concerns that patient information was sometimes not well communicated within the system.

Supplementary material: Topic guide

Results

The 40 interviewees comprised 6 orthopaedic surgeons; 6 anaesthetists; 7 orthogeriatricians; 3 physiotherapists; 4 occupational therapists; 1 emergency medicine consultant and 1 emergency department nurse; 3 advanced nurse practitioners (in orthogeriatrics, emergency medicine and orthopaedics and trauma); 1 ward manager, 1 matron, 2 service managers; 1 senior operating department practitioner (ODP); 1 discharge coordinator; 1 trauma coordinator; 1 orthopaedic registrar and 1 junior doctor. Between 5 and 15 participants took part from each hospital ('Springhill'=9, 'Radford'=11, 'Maplegrove'=15 and 'Newbridge'=5). Participants' characteristics are summarised by hospital to maintain anonymity (Table 1).

For peer review only

Table 1: Characteristics of healthcare professionals interviewed

**Professional Role**	**Number of professionals interviewed across each hospital**				**Time spent working in role**
	**01 Springhill**	**02 Radford**	**03 Maplegrove**	**04 Newbridge**	
Consultant geriatrician / Orthogeriatrician	3	2	1	1	3–18 years
ED Consultant	1				5 years
Band 8 ANP Emergency Department		1			6 years
Anaesthetist	1	3	1	1	7–25 years
Orthopaedic surgeon		2	4		5–11 years
Orthogeriatric Advanced Nurse Specialist	1				14 years
Physiotherapist	1		1	1	3–5 years
Occupational Therapist	1	1	1	1	2–8 years
MSK Matron	1				7 years
Service Manager		1	1		5 years
Ward Manager		1			5 years
Senior Theatre Practitioner			1		8 years
ANP			2		13–17 years
Orthopaedic Registrar			1		5 years
FY2 Doctor			1		3.5 months
Discharge coordinator			1		19 years
Trauma coordinator				1	5 years
**Total**	**9**	**11**	**15**	**5**	

Abbreviations: ANP: Advanced Nurse Practitioner; ED: Emergency Department; FY2: Foundation Year 2; MSK: Musculoskeletal.

We identified four core components of successful multidisciplinary teamwork for hip fracture care and one central underpinning concept: 1) defined roles and responsibilities, 2) information transfer processes, 3) shared goals, and 4) collaborative leadership. Across these, shared responsibility underpinned all activities (Figure 1). We approach the results through a series of propositions with illustrative quotations shown in Table 2.

For peer review only

Figure 1: Core components of successful multidisciplinary teamwork for hip fracture care

Please see separate file for figure

1. Defined roles and responsibilities

Proposition 1: Successful multidisciplinary teamwork depends on clarity in roles and responsibilities within the team.

Clear roles and responsibilities were achieved by using protocols and formalised care pathways. These were undermined when key components of the pathway were missing from care provision, these included management of analgesia, optimisation for surgery, and falls assessment. The extent to which protocols and pathway documents were used depended upon 'buy in', which was variable, and partially related to staff seniority, with greater reluctance among most senior staff. Reasons for variation included disagreement about patient management and desire for greater professional autonomy. Participants provided examples where uncertainties and disagreement about responsibilities created difficulties. Comprehensive induction and training on protocols and pathways, particularly for new staff and rotating junior doctors, provided clear information on "what to expect or adhere to". Clarity was also achieved through job planning that outlined the parameters of professional roles and defined responsibilities.

2. Information transfer processes

For peer review only

Proposition 2: Successful multidisciplinary teamwork depends on processes to facilitate the effective transfer and exchange of information to the 'right' individuals at the 'right' time. One of the primary objectives of care coordination was the effective transfer of information to inform patient management and decision-making. This was achieved via formal mechanisms including multidisciplinary team meetings, ward rounds and documentation, shared information systems, and informal and spontaneous communication.

Multidisciplinary team meetings in hospitals took a range of forms including trauma meetings of the trauma healthcare team, 'board rounds' (reviews of ward patients' progress in relation to discharge), 'huddles' (short briefings between team members), and discharge planning meetings (to plan for patients leaving hospital). Participants identified characteristics of successful multidisciplinary team meetings including establishing patient goals, regular attendance by a range of specialties, and effective management such as time management and documentation of outputs. However, these were threatened by lack of attendance from key professional groups due to competing commitments and a view that attendance was "a waste of time" as time would be better spent with patients, or because meetings often repeated issues without resolution.

Multidisciplinary team documentation was vital as a means to transfer patient information. However, patient documentation was not always accessible to relevant teams at key points in the care pathway, nor appropriately designed or consistently completed to capture all relevant information. Information transfer was facilitated through patient tracking systems, 'ward boards' displaying key patient information, and electronic patient records. Communication processes were undermined when IT systems did not cross boundaries

For peer review only

either within secondary care, or with external organisations such as primary care and community services, including when primary care records could not be accessed.

Multidisciplinary team ward rounds and 'huddles' provided opportunities to create shared knowledge and problem-solve. Participants advocated varied modes of communication to bring together professional groups at different stages of the care pathway.

Shared workspaces promoted frequent and spontaneous verbal communication. Allocating patients to specific hip fracture wards facilitated this exchange as healthcare professionals were concentrated together. Spontaneous communication was also achieved using technologies such as WhatsApp, mobile 'phone and email. However, success depended on the interpersonal skills of staff. Verbal communication was hindered by hierarchical relationships. While some senior members of staff emphasised lack of hierarchy on wards, one Advanced Nurse Practitioner (ANP) felt that ward staff were "wary" of discussing patient management with consultants. They felt this was due to ward staff lacking confidence, coupled with the belief in persistence of traditional hierarchy. The ANP therefore acted as a go-between while also encouraging ward staff to approach and communicate directly with consultants. 'Coordinators' functioned as key points of contact, providing continuity and daily communication between professional groups at different stages of the care pathway. Formal, named coordinator roles included trauma and discharge coordinators and hip fracture nurse specialists. Informal coordinating roles provided teams with an additional point of contact. In one site this included a named member of staff with responsibility for hip fracture patients. However, over reliance on individuals meant that when they were away, communication and coordination was compromised.

For peer review only

3. Shared goals

Proposition 3: Successful multidisciplinary teamwork depends on the existence of a shared and well-defined purpose within the team.

Shared goals were undermined by complex dynamics between different professional groups, particularly geriatric and orthopaedic staff who participants felt had different priorities. Several orthogeriatricians described orthopaedics as a specialty that focused on "fixing" the fracture, compared with their holistic approach. The structural divisions in NHS Trusts that placed staff within different 'directorates' were seen as reinforcing professional differences and impeding communication. Engagement with shared goals was also challenging due to staff transience, particularly of junior doctors.

Shared goals were encouraged through multiple processes. Daily multidisciplinary team meetings enabled staff to build shared goals and collaborative treatment plans for individual patients and to hear "different points of view". Multidisciplinary team training provided opportunities for staff to share knowledge and understanding. This was particularly evident in the training, mentoring and support for junior staff. Clinical leads were integral to bridging professional boundaries and creating a common professional identity across individual specialties. They did this by establishing shared priorities and goals in multidisciplinary team planning meetings, sharing monitoring and auditing results with colleagues across the care pathway, and using audit findings to drive service improvement. Communal efforts in monitoring and appraisal provided further opportunities to reinforce

For peer review only

shared priorities and goals, such as reviewing National Hip Fracture Database data—a national audit program to benchmark services, mortality events, internal audits and informal discussions about service improvement.

4. Collaborative leadership

Proposition 4: Successful multidisciplinary teamwork depends on collaborative leadership across several specialties and levels.

Successful multidisciplinary teamwork relied on presence of nominated departmental leads for hip fracture care. These formal clinical leads had a facilitative and motivational role and centralised hospital leadership created an environment to support collaboration. Participants advocated a formal model of leadership that was concentrated in small, mixed specialty teams across orthopaedic, orthogeriatric, aesthetic and nursing divisions. Participants identified specialties missing at this level, including anaesthetists. Therapy staff felt excluded at this level and thought that their representation could promote more effective collaboration. Senior level leadership was thought to promote commitment among colleagues at all levels.

Clinical service leads had a facilitative and motivational role in engaging healthcare professionals from a range of specialties and assembled teams to develop shared priorities and goals, joint protocols, audit priorities and mutually-owned quality improvement plans. Multidisciplinary team planning meetings were a key part of this process. Participants identified several attributes that enabled 'heroic' individuals to fulfil these roles, such as

For peer review only

410 "passion" and "drive". The visibility of clinical service leads within the service and respect for their clinical judgement legitimised their position and influenced their success in achieving these aims. Hospitals with one strong clinical service lead emphasised the importance of leadership development and succession planning to maintain team stability and performance.

416 Effective leadership on the wards depended on centralised leadership that created an environment to support collaboration. Support included providing sufficient capacity and resources for hip fracture care including capacity for staff to attend MDT meetings, supporting local leads to engage in service planning and training by building this into job plans and supporting service improvement initiatives and innovation to help provide a sense of shared purpose. Some participants expressed frustration that reimbursement from best practice tariffs for hip fracture care was not sufficiently reinvested into service development. Participants emphasised the need for designated hospital board executives who engaged in bidirectional communication with clinical service leads and championed hip fracture care to facilitate this. Several participants highlighted a lack of centralized leadership due to the transience of higher management roles and competing priorities within the Trust.

Table 2: Core components of successful multidisciplinary working for hip fracture care: illustrative quotations

**Defined roles and responsibilities**<br>*Proposition 1:* Successful multidisciplinary teamwork depends on clarity in roles and responsibilities within the team	
**Themes identified**	**Illustrative quotations (all names are pseudonyms)**
Effective use of hip fracture protocols and	"We have a good communication right from the start [of

For peer review only - http://bmjopen.bmj.com/site/about/guidelines.xhtml

formalised care pathways<br><br>'Buy in' of protocol and pathway documents<br><br>Comprehensive induction on protocols and pathways<br><br>Clear job planning to outline parameters of professional roles	the pathway] … it's very kind of clear cut whose responsibility is what." Sue, Band 8 advanced majors' practitioner, emergency department, Radford<br><br>"Not many people know what OT [occupational therapy] is and you've really got to fight sometimes about your professional role in things.… It's that you're just having to keep fighting, 'No, that's not for us. That's for somebody else'." Lois, trauma and orthopaedic ward occupational therapist, Newbridge<br><br>"There was a complete lack of understanding from the trauma and orthopaedic consultants as to an expectation from them that orthogeriatrics was my biggest priority. And I was saying, well it's not in my job plan to do it and there was a complete lack of understanding from them about that." Elaine, consultant geriatrician, Newbridge<br><br>"Well I think the protocols and pathways work really well if everybody adheres to it, but with having a large department with a variety of clinicians involved it's difficult to get a buy-in from everybody." Peter, consultant orthopaedic surgeon , Radford<br><br>"Trying to get everybody to sign up to say something will always happen, seems to be quite difficult at the moment.… It does just take time and the people who have recently come through training tend to be far more cohesive and wanting to have demonstrable pathways that can be audited and guide people who are more junior." Tania, consultant anaesthetist, Newbridge
**Information transfer processes**<br>*Proposition 2:* Successful multidisciplinary teamwork depends on processes to facilitate the effective transfer and exchange of information to the 'right' individuals at the 'right' time.	
**Themes identified**	**Illustrative quotations (all names are pseudonyms)**
Use of a range of multi-disciplinary team meetings forms including trauma meetings, focused 'board rounds', 'huddles', and discharge planning meetings<br><br>Effective multidisciplinary team meetings including establishing patient goals, regular attendance by a range of specialties and effective management<br><br>Accessible multidisciplinary team documentation that is completed consistently and appropriately designed<br><br>Shared information systems that cross organisational boundaries	"I'm involved in the daily trauma meetings every morning, so that is all the consultants, that's the service manager, that's the junior doctors and that's us as an ACP [Advanced Clinical Practice] team. And you talk about the patients…. Sometimes the consultants can talk over you and forget that you're actually telling them what they need to know." Jacqui, advanced nurse practitioner, Maplegrove<br><br>"I've got a bleep, they've got my mobile, so I'm contactable throughout the day. So people from wards will be ringing me to ask questions." Joanne, trauma coordinator, Newbridge<br><br>"I think sometimes that probably when some of the consultants or the anaesthetists come on the ward that

For peer review only

Multidisciplinary team ward rounds to create shared knowledge and problem-solve<br><br>Shared workspaces to promote verbal communication, including placing patients on a designated hip fracture ward<br><br>Spontaneous communication using technologies such as WhatsApp, mobile 'phone and email<br><br>Use of named coordinators as key points of contact, including trauma and discharge coordinators and hip fracture nurse specialists<br><br>Use of informal coordinators as points of contact	sometimes [ward staff are] a bit wary of approaching them." Barbara, advanced nurse practitioner, Maplegrove<br><br>[The trauma coordinator] role has sort of, it's found itself floundering a bit so we don't intend to remove it, we just intend to enhance it and make it more clear what the role [is]. Darren, service manager, Radford
**Shared goals**<br>*Proposition 3:* Successful multidisciplinary teamwork depends on the existence of a shared and well-defined purpose within the team	
**Themes identified**	**Illustrative quotations (all names are pseudonyms)**
Importance of dynamics between different professional groups<br><br>Impact of structural divisions within NHS Trusts that reinforce professional differences<br><br>Staff transience undermining shared goals<br><br>Use of daily multidisciplinary team meetings to build shared goals<br><br>Multidisciplinary team training to share knowledge and understanding<br><br>Importance of clinical leads in bridging professional boundaries<br><br>Communal efforts in monitoring and appraisal	"Essentially, I think we all are bound by sort of evidenced based [care], I think that is a common sort of thing and we all want the best for our patients. And I think those are two things which bind us that we all want to do the right thing for the patients." Dheeraj, consultant anaesthetist, Radford<br><br>"It's one of our major battles [as orthogeriatricians], the fact that we struggle to engage [with orthopaedic surgeons]. We struggle with them to engage with us, because you know basically they fix them and then walk away but occasionally we need them back and it's an ongoing battle, always has been." David, orthogeriatric advanced nurse specialist, Springhill<br><br>"I'm not a fan of the divisional structure of hospitals. I think it's quite artificial and hospitals are trying too hard to align themselves like a corporation would… I think it can [impede communication]." Andy, consultant orthopaedic surgeon, Maplegrove<br><br>"Our top consultant that works on [the hip fracture] ward, she's very driven by being the best, and having the best outcome for our patients. So I think everyone has a bit of a collective drive, but I think the pathway, being on that ward and on the pathway is a mega drive. Because it's constantly evaluated." Jane, occupational therapist, Springhill<br><br>"Updating your team is massively important. If you explain to your team why you're doing something, what you'd like done and why you'd like it done, that really

	helps. It's just that you've got that shared aim at the end of it. If you're working towards the shared aim, I think things work much better." Lucy, emergency medicine consultant, Springhill
**Collaborative leadership**<br>*Proposition 4:* Successful multidisciplinary teamwork depends on effective, collaborative leadership across several specialties and levels	
**Themes identified**	**Illustrative quotations (all names are pseudonyms)**
Importance of nominated departmental leads for hip fracture care<br><br>Formal model of leadership in small, mixed speciality teams<br><br>Clinical service leads with a facilitative and motivational role<br><br>Use of multidisciplinary team planning meetings<br><br>Importance of leadership development and succession planning to maintain stability<br><br>Centralised leadership providing sufficient capacity and resources for hip fracture care<br><br>Support for service improvement initiatives and innovation<br><br>Designated hospital board executives who engage with clinical service leads and champion hip fracture care	"I mean I think there is good engagement in the service at a senior level, and for that reason I think that focus, that sort of filters down across other members of the team." Kate, consultant geriatrician, Springhill<br><br>"I think if you get a core of people who are interested and enthusiastic, and I think that's across all disciplines, then there's no doubt that commitment can then drive improvements in other areas." Jon, consultant anaesthetist, Radford<br><br>"You've got to find key people [from a range of specialties] that are passionate about [hip fracture care] and get it. And even understand that, you know, the frailest of the frail, and you've got to do it properly and that takes lots of different services and lots of different professionals to pull that together … I guess I've been lucky here because people have put their head above the parapet rather than me pulling them in sort of kicking and screaming." Amanda, consultant orthogeriatrician, Springhill<br><br>"So, I think what worked well initially was a lot of buy-in and support from management. So three, four years ago one of the senior business unit managers, called in a couple of consultants that said, 'I'll support whatever you want to help this pathway better, what can we do about it?'… I think [it's helpful because] it brings the whole team together." Peter, consultant orthopaedic surgeon, Radford

5. Underpinning concept: Shared responsibility (Table 3)

Proposition 5: Roles and responsibilities, information transfer processes, shared goals and collaborative leadership all depend on a culture of shared responsibility for care delivery.

For peer review only

Underpinning and crossing all components, shared responsibility was evident and articulated. A culture of shared responsibility for hip fracture care ensured all members of the multidisciplinary team were invested in care delivery. This was enabled through care models where patients were cared for jointly by orthopaedic surgeons and orthogeriatricians. Staff valued the assistance of colleagues in performing tasks when needed, particularly when they were overloaded. Shared responsibility was also evidenced by colleagues providing feedback to improve performance and taking on supervisory roles. Orthogeriatricians were seen as occupying a key role in supervision of and support for advanced nurse practitioners, therapists and junior doctors (medical and surgical). There was evidence that traditional role boundaries were blurred. This included the involvement of nurses in mobilisation of patients when therapists were not available, and anesthetists' work to upskill emergency department doctors in nerve block provision.

For peer review only

Mutual trust and respect for one another's skills created an environment that encouraged staff to participate actively in the multidisciplinary team including in meetings, discussions with colleagues, and constructive management of disagreements. Trust and respect were based on a recognition of the value of different professional groups. Familiarity with colleagues, both professionally and personally, helped to build this trust. Informal relationships were facilitated through shared workspaces. Mutual trust and respect were often cultivated over time.

Table 3: Underpinning concept of shared responsibility: illustrative quotations

**Underpinning concept: Shared responsibility**

Proposition 5: Successful multidisciplinary teamwork depends on a culture of shared responsibility for care delivery	
**Themes identified**	**Illustrative quotations (all names are pseudonyms)**
Use of joint care models between orthopaedic surgeons and orthogeriatricians<br><br>Assistance from colleagues in performing tasks when needed<br><br>Provision of feedback to improve performance<br><br>Blurring of traditional role boundaries<br><br>Mutual trust and respect for one another's skills<br><br>Recognition of the value of different professional groups<br><br>Familiarity with colleagues to build trust	"When we're all talking together [as a team] sometimes we can go, 'Can you do this and we'll do that' and I suppose it's just supporting each other…. So again, sometimes it's just like, 'If you do this then that gives me time to do that' and we work that way." Jessica, occupational therapist, Radford<br><br>"You've got to listen to everybody and without certain people in that team it won't work, so I form a good rapport with the social workers, placement of care hub, so that you know they can trust me and I can trust them." Alice, foundation year 2 doctor, Maplegrove<br><br>I think you need to appreciate each other in terms of what people bring. You need to recognise other people's strengths and whether they're best placed to talk about something or you are yourself. Andy, consultant orthopaedic surgeon, Maplegrove<br><br>"I think there has to be an element of respect [for colleagues from different teams] as well, that in the good old days, a consultant was king and the staff were just, you know the minions, working minions, working around them but nowadays there's more of an equality in the relationship. So, I would probably quite happily give an opinion of the Nurse on the ward, if she has a query about a patient, cause she sees the patient every day, so there's quite an open communication both ways." Lance, consultant orthopaedic surgeon, Radford<br><br>"At one point I did start going to the trauma meeting for a bit. And actually that was really interesting for me because I did realise, hearing them talk about how specialised their knowledge was actually about operations and the operative management of problems. And I found that really interesting because I thought gosh, you know, these people do know a lot about what they did." Elaine, consultant geriatrician, Newbridge

Discussion

This study has characterised how hospitals deliver multidisciplinary work for hip fracture care and suggests core components needed to deliver this. Findings identified four

For peer review only

components to promote successful multidisciplinary teamwork: 1) defined roles and responsibilities, 2) information transfer processes, 3) shared goals, and 4) collaborative leadership. Shared responsibility underpinned and acted across all four components.

Relationship to current literature

This study complements existing work on optimal care models for hip fracture care by exploring how multidisciplinary teamwork can be organised and delivered in practice. Guidelines and existing research have highlighted the importance of implementing formal processes to coordinate care, including use of well-defined care pathways and protocols[5, 37] and good communication to facilitate information transfer[9]. The alignment of team goals through multi-disciplinary and clinical governance meetings and shared learning has also been identified within the acute rehabilitation of hip fracture patients. National Institute for Health and Care (NICE) implementation advice for hip fracture management and British Orthopaedic Association (BOA) guidelines have highlighted the importance of identifying an overarching clinical lead for hip fracture care, supported by senior leads from a range of specialties[9]. Coordination between orthogeriatricians and orthopaedic surgeons[38, 39] and formal roles such as trauma and discharge coordinators have been highlighted elsewhere as important [40, 41, 42].

Our findings also highlight possible barriers to effective multidisciplinary teamwork. These included some reluctance by more experienced staff to use hip fracture pathway documents or newly developed protocols, as well as barriers to communication presented by belief in hierarchy. Findings reflect wider systemic issues that have been identified in the context of

For peer review only

other multidisciplinary teams in the NHS. These include the existence of structural arrangements within and between secondary care and external organisations that hinder communication, such as incompatible computing systems[43], staff transience[17], and steep hierarchies that stymie communication within teams [44]. The socialisation of staff into different professional groups with distinct cultures, identities and priorities has further been identified as a hindrance to collaborative work[45]. The model of clinical service leadership described here reflects a broader trend in healthcare away from a 'command and control' approach to a motivational and facilitative style that involves building relationships with staff from a range of specialties[46]. It also reflects increasing recognition of the value of collaborative leadership in which leadership roles are shared between team members[47].

Hip fracture care includes separate roles with some evidence of interdisciplinary working through blurring of traditional role boundaries[19]. Core components of teamwork in this context encompass a range of skills (e.g., leadership), processes (e.g., hip fracture protocols and pathways, formal mechanisms for information transfer), and values (e.g., shared goals)[17]. Several strategies to support multidisciplinary teamwork are reflected in previous work, including interdisciplinary training[48], shared monitoring and evaluation processes[17] and familiarisation with colleagues to build trust[21].

Our study has identified the centrality of shared responsibility within multidisciplinary teams delivering care for hip fracture. Care processes such as multidisciplinary team meetings, monitoring and auditing processes and multidisciplinary team training can serve dual purposes by creating and sustaining shared responsibility as a value.

For peer review only

This work is taking place within a broader programme to develop a toolkit to enable service providers and commissioners to improve organisation and delivery of hip fracture services[27], results due in 2023. Based on our findings, we have developed our model into a how to guide of practical recommendations to for hip fracture care, including interdisciplinary 'exchange' training built into both orthopaedic and orthogeriatrics speciality training curricula (Supplementary material). This resonates with work focused on other conditions, including cancer, that require multidisciplinary teamwork[22, 49].

Supplementary material: A how to guide, a multidisciplinary toolkit for hip fracture care

Strengths and limitations

The sample, comprising 40 participants drawn from four UK hospitals, provided sufficient data to address the research area and included professionals with relevant experience, as such the study achieved information power[30]. Using an abductive analysis approach, rather than transposing data onto an existing theory or framework, ensured the results were data-driven such that data were not forced into pre-defined constructs[34]. Accordingly, where relevant, data were coded into more than one concept. We invited all healthcare professionals who working within the service to participate. However, it is possible that those who declined were less engaged in service delivery; we also note that others involved in care or discharge planning—such as social workers—did not take part and that numbers of allied health professionals (7 out of 40) were relatively low compared with their overall representation in trauma care. Despite these limitations, the study included

For peer review only

participants from diverse professional backgrounds, and the candid way that they spoke about challenges provided us with confidence in the veracity of the data. As data collection took place in hospitals that admitted moderate to large numbers of hip fracture patients, the transferability of findings to smaller units may be limited and there may be need for more work to understand fracture care in such settings[50].

Further research

Further work is needed to understand leadership configurations in hip fracture care, particularly the role of ‘emergent’ leaders who do not have formal roles but exert considerable influence within teams[51]. Future research is also needed in smaller contexts and we are planning to evaluate the impact of our toolkit on the organisation and delivery of hip fracture services.

Conclusions

This study used qualitative methods to develop a description of core components needed to deliver multidisciplinary teamwork for hip fracture care. It highlights the importance of creating and maintaining a culture that includes shared responsibility for care delivery. Our findings provide a basis for future training, skills development and assessment of multidisciplinary teamwork for hip fracture care in the UK.

Acknowledgements

For peer review only

We thank all the healthcare professionals who participated in this study and the REDUCE study group who provided support and guidance. The REDUCE study group consists of Dr Rita Patel, Professor Andrew Judge, Professor Antony Johansen, Dr Elsa M R Marques, Dr Petra Baji, Ms Jill Griffin, Ms Marianne Bradshaw, Dr Katie Whale, Mr Tim Chesser, Professor Xavier L Griffin, Dr Muhammad K Javaid and Professor Yoav Ben-Shlomo. We are grateful to members of the Patient Experience in Research Partnership (PEP-R), whose input shaped the research design and how it was carried out.

Author contributions

All authors contributed to the design of this research and contributed to the acquisition, analysis and/or interpretation of data. All authors contributed to drafting this work and revising it for important intellectual content, and all gave final approval for the version to be submitted. The authors comprise two social scientists with several years of experience in health research (SD, FF), a practicing consultant orthogeriatrician with academic expertise in epidemiology and population health research (CG), and a social anthropologist with expertise in qualitative and interdisciplinary research (RGH). We brought our different backgrounds into the research including in its design and conduct, including in interpretation of the data and results.

Competing interests

Authors have no competing interests to declare.

For peer review only

Funding

This work was supported by Versus Arthritis (reference 22086).

Data sharing statement

Anonymised interview data may be accessed via the University of Bristol Research Data Repository. Access to the data will be made available to researchers for ethically approved research projects, from a six-year embargo period, which ends on 1st April 2029. Data can be accessed on the understanding that confidentiality will be maintained and after a Data Access Agreement has been signed by an institutional signatory. No authentic request for access will be refused. Data are available at the University of Bristol data repository, data.bris, at https://doi.org/10.5523/bris.3f9j9z626s7f32a3r5j1wd7zt7.

Ethics approval

Ethical approval was provided by the Faculty of Health Sciences University of Bristol Research Ethics Committee (Ref: 108284) and by the NHS Health Research Authority (20/HRA/71). NHS settings (Trusts) for the four study sites each provided research governance approval.

Figure legend

Figure 1: Core components of successful multidisciplinary teamwork for hip fracture care

For peer review only

References

1. Gullberg, B., O. Johnell, and J.A. Kanis, *World-wide projections for hip fracture.* Osteoporos Int, 1997. **7**(5): p. 407-13.
2. Neuburger, J., et al., *The Impact of a National Clinician-led Audit Initiative on Care and Mortality after Hip Fracture in England: An External Evaluation using Time Trends in Nonaudit Data.* Medical Care, 2015. **53**(8): p. 686-691.
3. Griffin, X.L., et al., *Recovery of health-related quality of life in a United Kingdom hip fracture population. The Warwick Hip Trauma Evaluation--a prospective cohort study.* Bone Joint J,, 2015. **97-b**(3): p. 372-82.
4. Judge A, et al., *Models of care for the delivery of secondary fracture prevention after hip fracture: a health service cost, clinical outcomes and cost-effectiveness study within the South Central Region. National Institute for Health Research (NIHR), Health Services and Delivery Research (HS&DR).* 2016.
5. Kates, S.L., *Hip fracture programs: are they effective?* Injury, 2016. **47 Suppl 1**: p. S25-7.
6. National Institute for Health and Care Excellence (NICE), *Hip fracture in adults: Quality standard [QS16]*. 2017, Manchester: NICE.
7. National Institute for Health and Care Excellence (NICE), *Hip fracture: management: Clinical guideline [CG124]*. 2017: Manchester, NICE.
8. National Osteoporosis Society, *Effective secondary prevention of fragility fractures: clinical standards for fracture liaison services*. 2015: Bath: NOS.
9. Association, B.O., *BOAST 1 version 2-patients sustaining a fragility hip fracture.* London. British Orthopaedic Association, 2012.

For peer review only

10. Falls and Fragility Fracture Audit Programme (FFFAP), *National Hip Fracture Database (NHFD) annual report*. 2021: Royal College of Physicians, London.

11. *The care of patients with fragility fracture*. 2007: British Orthopaedic Association.

12. Christie, J., et al., *Improving the experience of hip fracture care: A multidisciplinary collaborative approach to implementing evidence-based, person-centred practice.*

13. Hordvik, H.B., et al., *Physiotherapists´ experiences with older adults´ rehabilitation trajectory after hip fracture: A qualitative study in Western Norway.* Physiotherapy Theory and Practice, 2023. **39**(2): p. 328-342.

14. Guerra, S., et al., *Multidisciplinary team healthcare professionals' perceptions of current and optimal acute rehabilitation, a hip fracture example A UK qualitative interview study informed by the Theoretical Domains Framework.* PLoS One, 2022. **17**(11): p. e0277986.

15. Willcocks, S.G., *Exploring team working and shared leadership in multi-disciplinary cancer care.* Leadersh Health Serv (Bradf Engl), 2018. **31**(1): p. 98-109.

16. Grand, J.H., S. Caspar, and S.W. Macdonald, *Clinical features and multidisciplinary approaches to dementia care.* Journal of multidisciplinary healthcare, 2011. **4**: p. 125-147.

17. Ellis, G. and N. Sevdalis, *Understanding and improving multidisciplinary team working in geriatric medicine.* Age and Ageing, 2019. **48**(4): p. 498-505.

18. Salas, E., D.E. Sims, and C.S. Burke, *Is there a "Big Five" in Teamwork?* Small Group Research, 2005. **36**(5): p. 555-599.

19. D'Amour, D., et al., *The conceptual basis for interprofessional collaboration: core concepts and theoretical frameworks.* J Interprof Care, 2005. **19 Suppl 1**: p. 116-31.

20. Lemieux-Charles, L. and W.L. McGuire, *What Do We Know about Health Care Team*

For peer review only

Effectiveness? A Review of the Literature. Medical Care Research and Review, 2006. **63**(3): p. 263-300.

21. D'Amour, D., et al., *A model and typology of collaboration between professionals in healthcare organizations.* BMC Health Serv Res, 2008. **8**: p. 188.
22. Rosen, M.A., et al., *Teamwork in healthcare: Key discoveries enabling safer, high-quality care.* The American psychologist, 2018. **73**(4): p. 4 Nilsen -450.
23. Soukup, T., et al., *Successful strategies in implementing a multidisciplinary team working in the care of patients with cancer: an overview and synthesis of the available literature.* J Multidiscip Healthc, 2018. **11**: p. 49-61.
24. Salas, E., D.L. Reyes, and S.H. McDaniel, *The science of teamwork: Progress, reflections, and the road ahead.* Am Psychol, 2018. **73**(4): p. 593-600.
25. Bélanger, E. and C. Rodríguez, *More than the sum of its parts? A qualitative research synthesis on multi-disciplinary primary care teams.* J Interprof Care, 2008. **22**(6): p. 587-97.
26. Coyne, I.T., *Sampling in qualitative research. Purposeful and theoretical sampling; merging or clear boundaries?* J Adv Nurs, 1997. **26**(3): p. 623-30.
27. Patel, R., et al., *REducing unwarranted variation in the Delivery of high qUality hip fracture services in England and Wales (REDUCE): protocol for a mixed-methods study.* BMJ Open, 2021. **11**(5): p. e049763.
28. Baker, S.E. and R. Edwards, *How many qualitative interviews is enough.* 2012.
29. *Snowball sampling*, in *The SAGE encyclopedia of social science research methods*, M.S. Lewis- Beck, Bryman, A., & Futing Liao, T. , Editor. 2004, Sage Publications, Inc.: Thousand Oaks, CA.
30. Malterud, K., V.D. Siersma, and A.D. Guassora, *Sample Size in Qualitative Interview*

Studies: Guided by Information Power. Qual Health Res, 2016. **26**(13): p. 1753-1760.
31. *Qualitative Researching with Text, Image and Sound*. 2000: London.
32. Tavory, I. and S. Timmermans, *Abductive analysis: Theorizing qualitative research*. 2014: University of Chicago Press.
33. Nilsen, P., *Making sense of implementation theories, models and frameworks.* Implementation Science, 2015. **10**(1): p. 53.
34. Edwards-Jones, A., *Qualitative data analysis with NVIVO*. 2014, Taylor & Francis.
35. Pope, C., S. Ziebland, and N. Mays, *Qualitative research in health care. Analysing qualitative data.* Bmj, 2000. **320**(7227): p. 114-6.
36. Gooberman-Hill, R., et al., *Involving patients in research: considering good practice.* Musculoskeletal Care, 2013. **11**(4): p. 187-90.
37. Meinberg, E., et al., *Hospital-based Hip fracture programs: Clinical need and effectiveness.* Injury, 2020. **51**: p. S2-S4.
38. Fernandez, M.A., X.L. Griffin, and M.L. Costa, *Management of hip fracture.* British Medical Bulletin, 2015. **115**(1): p. 165-172.
39. Chudyk, A.M., et al., *Systematic Review of Hip Fracture Rehabilitation Practices in the Elderly.* Archives of Physical Medicine and Rehabilitation, 2009. **90**(2): p. 246-262.
40. Riemen, A.H.K. and J.D. Hutchison, *The multidisciplinary management of hip fractures in older patients.* Orthopaedics and trauma, 2016. **30**(2): p. 117-122.
41. (UK), N.I.f.H.a.C.E., *Major Trauma: Service Delivery NICE Guideline NG40*. 2016.
42. (NICE), N.I.f.H.a.C.E., *Implementation advice; Implementing the Hip Fracture Programme*. 2012, NICE: Manchester.
43. Zhang, J., et al., *Interoperability in NHS hospitals must be improved: the Care Quality Commission should be a key actor in this process.* Journal of the Royal Society of

For peer review only

Medicine, 2020. **113**(3): p. 101-104.

44. Green, B., et al., *Challenging hierarchy in healthcare teams – ways to flatten gradients to improve teamwork and patient care.* British Journal of Oral and Maxillofacial Surgery, 2017. **55**(5): p. 449-453.

45. Powell, A.E. and H.T.O. Davies, *The struggle to improve patient care in the face of professional boundaries.* Social Science & Medicine, 2012. **75**(5): p. 807-814.

46. Storey, J. and R. Holti, *Towards a New Model of Leadership for the NHS.* 2013.

47. Günzel-Jensen, F., A.K. Jain, and A.M. Kjeldsen, *Distributed leadership in health care: The role of formal leadership styles and organizational efficacy.* Leadership, 2016. **14**(1): p. 110-133.

48. Reeves, S., et al., *Interprofessional education: effects on professional practice and healthcare outcomes.* Cochrane Database of Systematic Reviews, 2013(3).

49. *The Characteristics of an Effective Multidisciplinary Team (MDT)*. 2010; Available from: file:///C:/Users/sd17432/Downloads/NCATMDTCharacteristics_FINAL.pdf.

50. Forero, R., et al., *Application of four-dimension criteria to assess rigour of qualitative research in emergency medicine.* BMC Health Services Research, 2018. **18**(1): p. 120.

51. Hanna, A.A., et al., *The Emergence of Emergent Leadership: A Comprehensive Framework and Directions for Future Research.* Journal of Management, 2020. **47**(1): p. 76-104.

For peer review only

Figure 1: Core components of successful multidisciplinary teamwork for hip fracture care

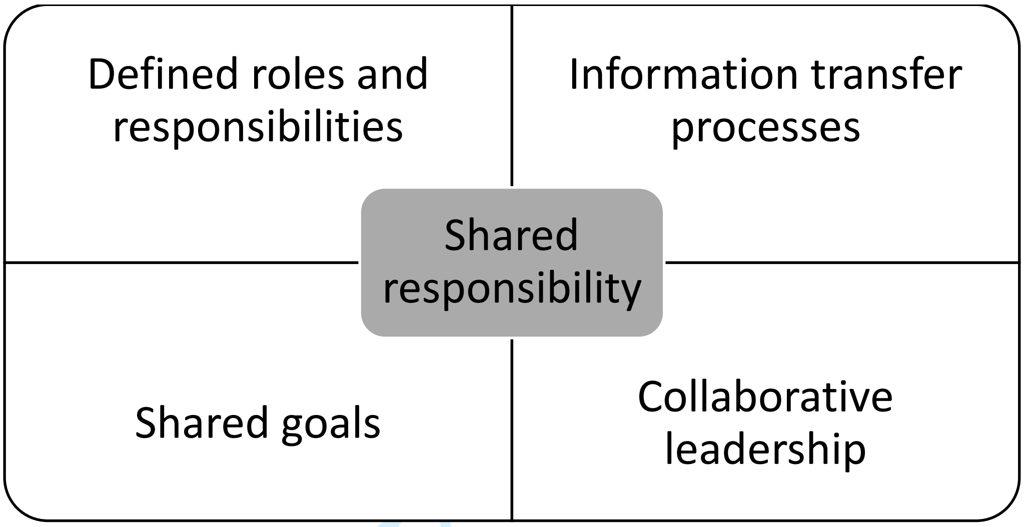

For peer review only

Supplementary material: Interview topic guide

Aims and objectives

- To investigate how hip fracture services are provided in different hospital settings and factors that help/ hinder ability to deliver care
- This study will provide us with information that can be used to improve the delivery of hip fracture services and reduce variation in care

Topics covered will be:

- Find out a bit about you
- Organisation of the care pathway
- Barriers and facilitators to service delivery
- Close

For peer review only

Part 1: About you

- Job title
- Part time/ full time/ %FTE
- Professional background (years since qualifying and progression)
- Years since qualifying for this role
- Years spent working in this service
- Age
- Date of birth
- Gender identify with
- Describe own role in delivering hip fracture care (before Covid)
    - Face to face duties
    - Administrative / organisational duties
    - Management duties

Part 2: Care pathway before Covid-19

- Things that worked well/ not so well/ improvements.

  Examples of components of care

  - Ambulance hand over
  - Emergency department factors [NB: Fast-tracking to ward]
  - Imaging services
  - Orthogeriatric services
  - Pre-operative care
  - Theatre and anaesthetic services
  - Orthopaedic & Trauma Services
  - Inpatient physiotherapy services and rehabilitation
  - Training/ confidence in the manual handling of patients
  - Links to dementia care, mental health services, diabetes care, dietetics, palliative care
  - Discharge systems and links to intermediate care
  - Temporary rehabilitation / care home provision
  - Post-discharge queries from patients
  - Re-admission processes for potential complications
  - Fracture Liaison Services/ secondary fracture prevention pathways
  - Falls services
  - Patient information and education
  - Community physiotherapy services and rehabilitation
  - Relationships with care homes – infection control protocols for new placements and return of patients to their original care homes

For peer review only

Part 3: Barriers and facilitators to service delivery before COVID-19

Things that worked well/ not so well/ improvements:

Communication

- How well did everyone communicate within service?
  - Within team
  - Other colleagues in MDT
  - Links with other services, e.g. dementia care, mental health services, diabetes care, palliative care
  - Community services and rehabilitation

  [Probes: MDT/ morbidity and mortality meetings, trauma rounds, multi-disciplinary paperwork, shared protocols, shared governance meetings, central coordinator]

  - What worked well/ not so well/ improvements

- Department management/ senior management

  - What worked well/ not so well/ improvements

- External organisations
  - Primary care
  - Community services and rehabilitation
  - Social care services

Relationships

- What does a 'good' working relationship mean to you?
- How well did everyone work together
  - Within team
  - Other colleagues in MDT
  - Department management/ senior management
- What sorts of things contributed to good working relationship with colleagues/

For peer review only

made it more difficult?

- Improvements

Commitment to delivering care

- To what extent did your colleagues do what was expected of them?

  [Probes: Extent to which the 36 hour target for hip fracture operation was pushed, extent individuals adhered to protocols]

  - Why do you think this was?

- How engaged/ enthusiastic do you think your colleagues were?

  - What helped/ made it more difficult?

Access to resources

- To what extent did you have access to all the resources needed to carry out work?

  [Probes: staff vacancies, theatre capacity, surgeon capacity, workload and material resources, access to medical and social care data],

  - If not, why not?
  - What did you do to address this challenge?

- To what extent did you/ team have access to the training and information needed to carry out work?

  [Probes: training, evidence base, understanding what is required of them]

- Training/ mentorship programmes in place

- What worked well/ not so well/ improvements

- How much of a priority do you feel that hip fracture care was for the?

  - Hospital Trust
  - Department
  - Boss

  - Why/ why not priority?

For peer review only

- Strategies to engage senior management

Service delivery over time

- Example quality improvement project
    - How did change occur?
- Example when you tried to initiate change but couldn't
    - Why?
- What monitoring and evaluation processes are in place?

[Probes: national initiatives, internal audit and governance processes, review of cases in morbidity and mortality meetings]

- What is the impact of these monitoring and evaluation processes on service delivery?

[Probes: Are these fed back to staff, do they effect change]

- Example of local clinical audit – why initiated?
- What clinical governance activities are in place for hip fracture care?

[Probes: risk management, transparency, quality improvement, mortality reviews, strategies to review clinical incidents/ complaints]

How useful are they?

Part 4: Close

1. If you could change one thing about your (pre-Covid) service, what would it be?
2. Are there any other things or insights that you would like to tell me about today?
3. Do you have any questions for me about the project?
4. Thank them
5. Reaffirm confidentiality

For peer review only

For peer review only

For peer review only

Supplementary material: A 'how to' guide for effective multi-disciplinary team working in hip fracture care

Delivering hip fracture care requires multi-disciplinary teamwork, including effective communication and coordination across a range of specialties and professional roles including emergency medicine, orthopaedic surgery, anaesthetics, geriatric medicine, rehabilitation and nurses. The range of the multidisciplinary team involved reflects the complex needs of patients with hip fracture. Clinical guidelines recommend timely hip fracture care, with when needed, rapid transfers of care between professionals. Streamlining multi-disciplinary care is crucial to the delivery of safe and effective patient care.

There are four components needed for coordinated multi-disciplinary team (MDT) working. These are all underpinned by one central concept: shared responsibility. The responsibility for delivering these components lies with *all members* of the MDT.

Action 1: Clearly define roles and responsibilities of each member of the MDT

Roles and responsibilities are more than what is simply listed in a job description. Each member of the MDT needs to understand the other MDT member's roles and responsibilities. Hence, roles and responsibilities need to be appreciated and explained during induction processes.

Roles and responsibilities need to be co-agreed between an individuals' line manager, within the MDT and outlined in personalised job plans. These job plans should be reviewed at annual appraisals with modifications made according to MDT developments/ progress.

The clinical lead needs to have oversight of all roles and responsibilities in order to identify gaps in the pathway of hip fracture care, so as to manage the skillset within the MDT to ensure those gaps are filled.

Action 2: Set up systems to ensure clear, timely MDT communication

Hip fracture patients have complex medical and surgical needs and are often acutely unstable. Therefore, MDT communication must be dynamic and efficient with the capacity for immediate response from senior team members when necessary. Systems are enhanced by the presence of trauma coordinators and discharge coordinators with defined roles and responsibilities.

Face-to-face communication events (verbal)
Systems need to be established for effective communication events with those attending pre-agreed. Communication events should begin promptly, using the same standardised

For peer review only

format each time. Job plans need to permit attendance. Examples of communication events include daily trauma meetings, early morning board rounds ('huddles') and pre-arranged discharge planning meetings. Short meetings are more efficiently conducted whilst standing, where ability permits.

Six principles of effective MDT communication events include:

1. Ensure representation from the key disciplines
2. Establish patient centred goals
3. Bring opinions together openly
4. Agree an overall aim, next steps with time frames
5. Allocate responsibilities
6. Communicate/ document outputs

Written MDT communication
Written MDT documentation should be appropriately co-designed to capture all relevant information. All patient admissions should be clerked using a standardised proforma co-agreed and designed by the MDT with sections addressing trauma at other skeletal sites (besides the hip), nerve block use, osteoporosis management and orthogeriatric and orthopaedic senior reviews. Documents need to be available to the wider MDT. Examples of documents/ proformas that can assist the delivery of care include:

- Structured admission clerking proforma
- Structured post-operative junior doctor review (for patients returning to the ward from theatre)
- Structured falls and bone health assessments

Examples of documents are available on the National Hip Fracture Database (NHFD): https://www.rcplondon.ac.uk/projects/outputs/national-hip-fracture-database-nhfd-improvement-repository

Electronic MDT communication
Smart Phones have made radio pagers redundant. All staff should have access to a Smart Phone. Information can be managed securely through e.g. Siilo for patient specific information. WhatsApp can be used for team communication, but not patient specific information. Mobile phone numbers should be routinely shared within the MDT. Appropriate groups should be set-up.

Hospitals need to invest in electronic communication systems e.g. (i) to enable trauma wards to have live monitoring of theatre activity, to plan prompt transfers to and from theatre, and (ii) routine access to primary care records. Templated discharge summaries, e.g. with use of standardised phrases, can improve efficiency and increase communication at discharge.

Hip fracture patients are best managed on a hip fracture ward where all members of the MDT can be co-located and thus easily found for spontaneous communication, with nearby office space. Timetabling of consultant ward rounds and daily ward activities should be shared amongst the whole team and adhered to.

For peer review only

Action 3: Establish inter-disciplinary principles of working

Healthcare teams can be 'multidisciplinary ' or 'interdisciplinary'. Multi-disciplinary teams have separate roles and work within their own specific disciplines. Interdisciplinary teams work more closely together to deliver shared goals and some roles may overlap.

Where human resource permits, hip fracture care should be provided through a shared care model between orthopaedic and orthogeriatric services.

Orthopaedic, geriatric and anaesthetic specialist trainees should receive formal inter-disciplinary training to ensure mutual understanding of each other's roles, e.g. geriatric trainees should attend a trauma meeting and a hip fracture theatre session, whilst orthopaedic trainees should join an orthogeriatric ward round and geriatrician lead white board rounds. Similarly, an orthogeriatric trainee should spend time with an orthopaedic physiotherapist whilst the anaesthetic trainee should join the orthogeriatric ward round. The ultimate goal of interdisciplinary training is to ensure mutual understanding and professional support between roles.

All trainees should attend clinical governance meetings to understand governance structured necessary to support a complex pathway of patient care.

Examples of inter-disciplinary upskilling include:

- Nurses should be upskilled by physiotherapists to confidently mobilise patients post-operatively
- Emergency department doctors should be upskilled by anaesthetists to provide emergency nerve blocks
- Physiotherapists should be upskilled by occupational therapists to assist discharge planning
- Orthopaedic trainees should be upskilled by orthogeriatricians to assess cognitive capacity to consent

Agree cross-cover arrangements within clinical competencies, e.g. physiotherapy and occupational therapy co-working, medical and surgical junior doctors cross-covering, nursing staff empowered to mobilise patients.

Action 4: Establish collaborative MDT team leadership

Orthogeriatric (1), orthopaedic (2), anaesthetic (3) consultant hip fracture leads should have their role defined within their job plan. The orthopaedic and/or orthogeriatric consultant should identify as clinical (co-)lead for hip fracture services.

Strong clinical leadership is essential to co-ordinate effective MDT hip fracture care delivery. Formal leadership training is strongly encouraged.

A physiotherapy (4), nursing (5), occupational therapy (6) and pharmacy (7) discipline lead

For peer review only

for hip fracture care should be appointed.

All seven of these professionals have responsibility to attend clinical governance meetings and act as conduit for feedback from and to their individual specialist teams.

All disciplinary leads should consider succession planning and discuss this with the leadership team to manage leadership transitions smoothly and efficiently (no discipline should be without hip fracture leadership). Aim for early identification of successors with structured mentorship with staged handover.

Identify a hospital board executive responsible for bidirectional communication with the hip fracture clinical lead, who will champion hip fracture care at board level. All seven discipline-specific hip fracture leads should have met with the hospital board executive. The primary point of contact for the hospital board executive should be the hip fracture service clinical lead.

Action 5: Jointly agree the goals of the team and strategies to achieve these goals

Define MDT goals by jointly reviewing NHFD performance, bench-marked KPIs, and how these compare against national averages, as well as REDUCE toolkit targets and any specialty-specific internal audit/ QI data. It's really important to get all members of the team in the room and to hear the voices and perspectives of each MDT discipline with no one specialty dominating the conversation. The clinical lead for hip fracture services holds the responsibility for coordinating this process.

Strategies to improve services should be structured into short, medium and long-term approaches and achievements routinely reviewed at clinical governance and morbidity and mortality (M&M) meetings with plans and progress clearly communicated to the hip fracture service hospital board executive. The REDUCE toolkit is designed to facilitate each hospital to develop their own individualised strategies towards service improvement.

For peer review only

Supplementary Material: Consolidated criteria for reporting qualitative studies (COREQ): 32-item checklist

Please indicate in which section each item has been reported in your manuscript. If you do not feel an item applies to your manuscript, please enter N/A.

For further information about the COREQ guidelines, please see Tong et al., 2017: https://doi.org/10.1093/intqhc/mzm042

No.	Item	Description	Section #
**Domain 1: Research team and reflexivity**			
Personal characteristics			
1.	Interviewer/facilitator	Which author/s conducted the interview or focus group?	p. 10
2.	Credentials	What were the researcher's credentials? *E.g. PhD, MD*	p. 1
3.	Occupation	What was their occupation at the time of the study?	p.10
4.	Gender	Was the researcher male or female?	N/A
5.	Experience and training	What experience or training did the researcher have?	p. 1 /ORCID
Relationship with participants			
6.	Relationship established	Was a relationship established prior to study commencement?	No
7.	Participant knowledge of the interviewer	What did the participants know about the researcher? *E.g. Personal goals, reasons for doing the research*	N/A
8.	Interviewer characteristics	What characteristics were reported about the interviewer/facilitator? *E.g. Bias, assumptions, reasons and interests in the research topic*	Yes, acknowledgement
**Domain 2: Study design**			
Theoretical framework			
9.	Methodological orientation and theory	What methodological orientation was stated to underpin the study? *E.g. grounded theory, discourse analysis, ethnography, phenomenology, content analysis*	p. 11
Participant selection			
10.	Sampling	How were participants selected? *E.g. purposive, convenience, consecutive, snowball*	p. 9
11.	Method of approach	How were participants approached? *E.g. face-to-face, telephone, mail, email*	p. 9
12.	Sample size	How many participants were in the study?	p. 10
13.	Non-participation	How many people refused to participate or dropped out? What were the reasons for this?	p. 10
Setting			
14.	Setting of data collection	Where was the data collected? *E.g. home, clinic, workplace*	p. 10
15.	Presence of non-participants	Was anyone else present besides the participants and researchers?	No

Formatted: Font: 6 pt

16.	Description of sample	What are the important characteristics of the sample? *E.g. demographic data, date*	p. 9, p. 12
Data collection			
17.	Interview guide	Were questions, prompts, guides provided by the authors? Was it pilot tested?	p. 10 - 11
18.	Repeat interviews	Were repeat interviews carried out? If yes, how many?	N/A
19.	Audio/visual recording	Did the research use audio or visual recording to collect the data?	p. 11
20.	Field notes	Were field notes made during and/or after the interview or focus group?	No
21.	Duration	What was the duration of the interviews or focus group?	p. 10
22.	Data saturation	Was data saturation discussed?	p. 10
23.	Transcripts returned	Were transcripts returned to participants for comment and/or correction?	No
**Domain 3: analysis and findings**			
Data analysis			
24.	Number of data coders	How many data coders coded the data?	p. 11
25.	Description of the coding tree	Did authors provide a description of the coding tree?	p. 11
26.	Derivation of themes	Were themes identified in advance or derived from the data?	p. 11
27.	Software	What software, if applicable, was used to manage the data?	p. 11
28.	Participant checking	Did participants provide feedback on the findings?	No
Reporting			
29.	Quotations presented	Were participant quotations presented to illustrate the themes / findings? Was each quotation identified? *E.g. Participant number*	Boxes 1 - 3
30.	Data and findings consistent	Was there consistency between the data presented and the findings?	Yes
31.	Clarity of major themes	Were major themes clearly presented in the findings?	Yes
32.	Clarity of minor themes	Is there a description of diverse cases or discussion of minor themes?	Yes

Developed from: Allison Tong, Peter Sainsbury, Jonathan Craig, Consolidated criteria for reporting qualitative research (COREQ): a 32-item checklist for interviews and focus groups, International Journal for Quality in Health Care, Volume 19, Issue 6, December 2007, Pages 349–357, https://doi.org/10.1093/intqhc/mzm042

For peer review only

BMJ Open

A model of multidisciplinary teamwork in hip fracture care: a qualitative interview study

Journal:	*BMJ Open*
Manuscript ID	bmjopen-2022-070050.R2
Article Type:	Original research
Date Submitted by the Author:	14-Sep-2023
Complete List of Authors:	Drew, Sarah; University of Bristol, Translational Health Sciences<br>Fox, Fiona; University of Bristol, Bristol Medical School (PHS)<br>Gregson, Celia; University of Bristol, Musculoskeletal Research Unit; Royal United Hospital Bath NHS Trust, Older Person's Unit<br>Gooberman-Hill, Rachael; University of Bristol, Bristol Medical School
**Primary Subject Heading**:	Health services research
Secondary Subject Heading:	Qualitative research
Keywords:	Organisation of health services < HEALTH SERVICES ADMINISTRATION & MANAGEMENT, Hip < ORTHOPAEDIC & TRAUMA SURGERY, QUALITATIVE RESEARCH

SCHOLARONE™
Manuscripts

I, the Submitting Author has the right to grant and does grant on behalf of all authors of the Work (as defined in the below author licence), an exclusive licence and/or a non-exclusive licence for contributions from authors who are: i) UK Crown employees; ii) where BMJ has agreed a CC-BY licence shall apply, and/or iii) in accordance with the terms applicable for US Federal Government officers or employees acting as part of their official duties; on a worldwide, perpetual, irrevocable, royalty-free basis to BMJ Publishing Group Ltd ("BMJ") its licensees and where the relevant Journal is co-owned by BMJ to the co-owners of the Journal, to publish the Work in this journal and any other BMJ products and to exploit all rights, as set out in our licence.

The Submitting Author accepts and understands that any supply made under these terms is made by BMJ to the Submitting Author unless you are acting as an employee on behalf of your employer or a postgraduate student of an affiliated institution which is paying any applicable article publishing charge ("APC") for Open Access articles. Where the Submitting Author wishes to make the Work available on an Open Access basis (and intends to pay the relevant APC), the terms of reuse of such Open Access shall be governed by a Creative Commons licence – details of these licences and which Creative Commons licence will apply to this Work are set out in our licence referred to above.

Other than as permitted in any relevant BMJ Author's Self Archiving Policies, I confirm this Work has not been accepted for publication elsewhere, is not being considered for publication elsewhere and does not duplicate material already published. I confirm all authors consent to publication of this Work and authorise the granting of this licence.

For peer review only

Title: A model of multidisciplinary teamwork in hip fracture care: a qualitative interview study

Short title: Multidisciplinary teamwork in hip fracture care

Corresponding author

Dr Sarah Drew

Research Fellow

Musculoskeletal Research Unit

Bristol Medical School, University of Bristol

Learning and Research Building, Level 1

Southmead Hospital

BS10 5NB

Mobile: + 44 (0)7891149828

Email: sarah.drew@bristol.ac.uk

Sarah Drew[1], MA, DPhil

Fiona Fox[1], BSc, PhD

Celia L Gregson[1], B.Med.Sci., M.B.,B.S., MSc, FRCP, PhD

Rachael Gooberman-Hill[1,2], MA, PhD

Author affiliations

1. Musculoskeletal Research Unit, Translational Health Sciences, Bristol Medical School, University of Bristol, Bristol, UK.
2. UK National Institute for Health Research Bristol Biomedical Research Centre,

For peer review only

University Hospitals Bristol and Weston NHS Foundation Trust and the University of Bristol, Bristol, UK.

Word count 3998 (max 4000)

Keywords

Hip fractures, multidisciplinary teamwork, service delivery, qualitative

For peer review only

Abstract 300 words

Objectives

Hip fractures are common injuries in older age with high mortality requiring multidisciplinary clinical care. Despite guidance, there is considerable variation in hip fracture services and patient outcomes; furthermore, little is known about how successful multidisciplinary working can be enabled. This study aimed to characterise professionals' views about the core components of multidisciplinary teamwork in hip fracture care.

Design

The study comprised qualitative interviews with healthcare professionals delivering hip fracture care. Interviews were audio-recorded, transcribed and analysed abductively: material was coded inductively and grouped into higher level concepts informed by theories and frameworks relating to teamwork.

Setting

Four hospitals in England.

Participants

Participants were 40 healthcare professionals including orthopaedic surgeons, orthogeriatricians, physiotherapists and service managers.

Results

Results identified four components of successful multidisciplinary teamwork: 1) defined roles and responsibilities, 2) information transfer processes, 3) shared goals, and 4) collaborative leadership. These were underpinned by a single concept: shared responsibility, Defined roles and responsibilities were promoted through formal care pathways, reinforced through induction and training with clear job plans outlining tasks. Information transfer

For peer review only

processes facilitated timely information exchange to appropriate individuals. Well-defined, common purpose was hindered by complex inter-disciplinary professional relationships, particularly between orthogeriatric and orthopaedic staff, and encouraged through multidisciplinary team meetings and training. Clinical service leads were integral to bridging interdisciplinary boundaries. Mutual trust and respect were based on recognition of the value of different professional groups. Teamwork depended upon formal clinical leads with facilitative and motivational roles, and on hospital leadership that created an environment supporting collaboration. Shared responsibility for patients was encouraged by joint orthopaedic and orthogeriatric care models. Staff shared responsibility by assisting colleagues when needed.

Conclusions

Shared responsibility across the multidisciplinary team is fundamental to delivery of hip fracture care. Findings will inform development of clinical practice recommendations and training to build teamworking competencies.

Article summary

Strengths and limitations of this study

- Qualitative study design and methods using an abductive approach provided an in-depth understanding of care processes from the viewpoints of involved professionals and enabled the research to be informed by existing frameworks about teamwork.
- The sample, comprising 40 participants drawn from four UK hospitals, achieved information power through inclusion of a range of professionals with relevant experience and with sufficient data to address the research aim.
- Despite achieving information power, there are some professionals involved in

For peer review only

healthcare and related discharge planning—such as social workers—who were not included in the study, meaning that we were unable to include their views.

- Data collection was conducted in hospitals that admitted moderate to large numbers of hip fracture patients and this may limit transferability of findings to smaller units.

Funding statement

This work was supported by Versus Arthritis (reference 22086).

Competing interests

All authors declare that they have no competing interests.

Introduction

Globally, hip fractures pose a major challenge to patients and healthcare systems, with 4.5 million such fractures expected to occur annually by 2050[1]. Hip fractures are most

For peer review only

commonly sustained by older individuals and can be devastating with considerable impact on quality of life and a high risk of subsequent death[2, 3]. Provision of hip fracture care is costly to healthcare providers, incurring $5.96 billion in direct medical costs each year in the United States and £1.2 billion in the United Kingdom (UK)[4]. Comprehensive multidisciplinary hip fracture care pathways reduce postoperative complications and mortality[5]. To make recommendations about how to further enhance the quality care of care for people who have had hip fracture, there is a need to understand how healthcare professionals implement care pathways.

In the UK, thorough guidance has been published on the management of hip fracture[6-8]. Despite this, considerable variation exists in the organisation of care pathways[7, 9] and in patient outcomes[10]. Hip fracture care requires communication and coordination across a range of specialties and professional roles including emergency medicine, surgery, anaesthetics, geriatric medicine and rehabilitation[6, 7]. This multidisciplinary care reflects patients' complex needs that may include multiple pre-existing conditions[11]. Guidelines recommend prompt care, with rapid transfers of care between professionals[7, 11].

Studies about provision of acute rehabilitation for hip fracture with clinical leaders and physiotherapists have highlighted the importance of communication between professionals and patients in rehabilitation for hip fracture[12, 13]. Similarly, a recent interview-based study by Guerra and colleagues with healthcare professionals explored acute rehabilitation and implementation[14]. The study found that communication and collaboration are central to provision of effective rehabilitation and that organisational constraints are seen as a barrier to such provision. Other studies have also explored how patients and carers engage

For peer review only

with hip fracture care, and qualitative findings highlight the emotional and practical challenges associated with hip fracture[6-8]. Together, these studies highlight the importance of understanding the way in which care for hip fracture is organised and delivered.

High quality multidisciplinary care is crucial to delivery of safe and effective services. Multidisciplinary teamwork is necessary in delivery of clinical care for a number of health conditions and specialties, such as cancer[15], dementia[16] and in older people's services[17]. 'Teamwork' can be defined as 'a set of inter-related thoughts, actions, and feelings of each team member that are needed to function as a team and that combine to facilitate coordinated, adaptive performance and task objectives resulting in value-added outcomes'[18]. Collaborative teamwork exists on a continuum of professional autonomy and integrative working. Three broad models describe collaborative teamwork: 'multidisciplinary', whereby multiple specialties with separate roles work within their own disciplines; 'interdisciplinary' that involves working together to deliver shared goals with some blurring of disciplinary boundaries; and 'transdisciplinary' where professional roles are shared[19]. Research has highlighted how different tasks across clinical disciplines shape how teams are organised and work together[20].

Existing theories and frameworks guide professionals in the organisation and evaluation of the processes used in multidisciplinary team communications, training and leadership. Some frameworks are generalisable across several areas[18, 20-22], others have been developed for specific clinical disciplines[23]. Frameworks have identified diverse factors that promote clinical teamwork, including a shared understanding of team goals[18, 19, 21], successful

For peer review only

information exchange[18], effective leadership[17, 18, 21, 23] and adaptability of services to meet changing demands[18, 24, 25]. Ellis and Sevdalis build on frameworks to identify core technical and non-technical skills to facilitate effective multidisciplinary teamwork in geriatric medicine[17]. Non-technical skills include social, cognitive and personal resource skills. Strategic domains to enhance multidisciplinary working are skills (e.g., good leadership), processes (e.g., good documentation governance) and values (e.g., prioritising patient needs and respect for colleagues). Evidence from behavioural research into multidisciplinary teamwork suggests that if any of these three components is missing, teamwork will be unsuccessful[17].

Given the importance of multidisciplinary teams in hip fracture care and previous learning from other fields, we aimed to develop a model of the core components needed to deliver successful multidisciplinary teamwork in hip fracture care in England. Understanding views about successful teamwork in the hip fracture care pathway will help to provide visibility to core components and inform future work to support and enhance multidisciplinary care for hip fracture.

Methods

We used qualitative interviews to collect and interpret insights from health professionals engaged in delivery of hip fracture care in hospitals in England.

Sample

For peer review only

Hospital sites

Healthcare professionals involved in the organisation and delivery of hip fracture were sampled from four National Health Service (NHS) hospitals across England. The NHS provides almost all hip fracture care in England, free to patients at the point of receipt.

Purposive sampling was used to identify study sites[26], with variation in geography, number of hip fracture presentations per year, service configuration and audited hip fracture outcomes[27]. Three hospitals were urban and one rural. One hospital admitted a large number (>75th percentile), and three moderate (between 25th-75th percentile) numbers of hip fracture patients annually. Hospitals are identified by pseudonyms to retain anonymity ('Springhill', 'Radford', 'Maplegrove' and 'Newbridge').

Participants

Forty interviews were conducted with healthcare professionals across the four hospitals. Staff were purposively sampled using a criterion approach to include healthcare professionals from across the care pathway, including orthopaedic surgeons, anaesthetists, orthogeriatricians, physiotherapists and occupational therapists, nurses, trauma coordinators and service managers[28].

Potential participants were identified by a designated contact working within the service at each hospital site. Staff were invited to participate in the study by email, followed-up with reminder emails a minimum of two weeks later. Snowball sampling was also used[29], with

For peer review only

participants recommending other professionals involved in service delivery. In total, 75 healthcare professionals were approached to take part in the study, of whom, 40 (53%) agreed to take participate. The remainder either declined or were unavailable. The final sample enabled achievement of information power as it was adequate to address the study aim through inclusion of appropriate participants across the care pathway [30].

Ethical approval

Ethical approval was provided by the Faculty of Health Sciences University of Bristol Research Ethics Committee (Ref: 108284) and by the NHS Health Research Authority (20/HRA/71). Written informed consent was provided by all participants, confirming understanding of voluntary participation and potential publication of anonymised interview quotations. NHS settings (Trusts) for the four study sites each provided research governance approval.

Data collection

Two qualitative researchers (FF and SD) conducted interviews by telephone or teleconferencing technology (e.g., Microsoft teams), necessitated by Covid-19 pandemic restrictions. Interviews, lasting 45 to 90 minutes, were guided by a topic guide based on domains agreed collaboratively with healthcare professionals involved in delivery and organisation of hip fracture services. Part 1 focused on barriers and facilitators to the implementation of care. Part 2 covered multidisciplinary communication and cooperation including questions about access to resources, monitoring and evaluation (Supplementary

For peer review only

material). Interviews were flexible to explore emerging ideas[31] and questions relating to teamwork were covered in all domains.

Data analysis

Interviews were audio-recorded, transcribed and anonymised to remove any identifying information. Participants were assigned pseudonyms. Data were analysed using an abductive approach [32]. Abduction is an approach that involves using research findings to develop new theories that build upon existing theories and frameworks[33]. Further detailed explanation of abduction can be found in Tavory and Timmermans[32]. There are several stages to the abductive process. First, transcripts were imported into NVivo qualitative data management software[34] and coded using an inductive thematic approach[35]. In this process, we focused on aspects of the data relating to teamwork, which we found in all interviews. Next, codes and coded material was grouped into higher level concepts. In keeping with an abductive approach, the concepts were informed by existing frameworks about teamwork[17, 18, 21]. A model was then constructed to illustrate how core concepts contributed to multidisciplinary teamwork for hip fracture care (See Figure 1). During this process all transcripts were analysed in the same way with the same staged process applied. To ensure rigor, 40% transcripts were independently analysed in duplicate (by SD and FF) and themes reviewed and refined to reach an agreed code list[35].

Patient and public involvement

To refine the study design and data collection materials, we held three meetings with

For peer review only

members of 'The Patient Experience Partnership in Research' (PEP-R) group, a dedicated musculoskeletal patient involvement group[36]. Members' input helped to shape study documentation, and they felt that findings resonated with their own experiences, particularly reflecting their concerns that patient information was sometimes not well communicated within the system.

Supplementary material: Topic guide

Results

The 40 interviewees comprised 6 orthopaedic surgeons; 6 anaesthetists; 7 orthogeriatricians; 3 physiotherapists; 4 occupational therapists; 1 emergency medicine consultant and 1 emergency department nurse; 3 advanced nurse practitioners (in orthogeriatrics, emergency medicine and orthopaedics and trauma); 1 ward manager, 1 matron, 2 service managers; 1 senior operating department practitioner (ODP); 1 discharge coordinator; 1 trauma coordinator; 1 orthopaedic registrar and 1 junior doctor. Between 5 and 15 participants took part from each hospital ('Springhill'=9, 'Radford'=11, 'Maplegrove'=15 and 'Newbridge'=5). Participants' characteristics are summarised by hospital to maintain anonymity (Table 1).

Table 1: Characteristics of healthcare professionals interviewed

Professional Role	Number of professionals interviewed across each hospital	Time spent working in role

For peer review only

	01 Springhill	02 Radford	03 Maplegrove	04 Newbridge	
Consultant geriatrician / Orthogeriatrician	3	2	1	1	3–18 years
ED Consultant	1				5 years
Band 8 ANP Emergency Department		1			6 years
Anaesthetist	1	3	1	1	7–25 years
Orthopaedic surgeon		2	4		5–11 years
Orthogeriatric Advanced Nurse Specialist	1				14 years
Physiotherapist	1		1	1	3–5 years
Occupational Therapist	1	1	1	1	2–8 years
MSK Matron	1				7 years
Service Manager		1	1		5 years
Ward Manager		1			5 years
Senior Theatre Practitioner			1		8 years
ANP			2		13–17 years
Orthopaedic Registrar			1		5 years
FY2 Doctor			1		3.5 months
Discharge coordinator			1		19 years
Trauma coordinator				1	5 years
**Total**	**9**	**11**	**15**	**5**	

Abbreviations: ANP: Advanced Nurse Practitioner; ED: Emergency Department; FY2: Foundation Year 2; MSK: Musculoskeletal.

We identified four core components of successful multidisciplinary teamwork for hip fracture care and one central underpinning concept: 1) defined roles and responsibilities, 2) information transfer processes, 3) shared goals, and 4) collaborative leadership. Across these, shared responsibility underpinned all activities (Figure 1). We approach the results through a series of propositions with illustrative quotations shown in Tables 2 and 3.

Figure 1: Core components of successful multidisciplinary teamwork for hip fracture care

Please see separate file for figure

For peer review only

1. Defined roles and responsibilities

Proposition 1: Successful multidisciplinary teamwork depends on clarity in roles and responsibilities within the team.

Clear roles and responsibilities were achieved by using protocols and formalised care pathways. These were undermined when key components of the pathway were missing from care provision, these included management of analgesia, optimisation for surgery, and falls assessment. The extent to which protocols and pathway documents were used depended upon 'buy in', which was variable, and partially related to staff seniority, with greater reluctance among most senior staff. Reasons for variation included disagreement about patient management and desire for greater professional autonomy. Participants provided examples where uncertainties and disagreement about responsibilities created difficulties. Comprehensive induction and training on protocols and pathways, particularly for new staff and rotating junior doctors, provided clear information on "what to expect or adhere to". Clarity was also achieved through job planning that outlined the parameters of professional roles and defined responsibilities.

2. Information transfer processes

Proposition 2: Successful multidisciplinary teamwork depends on processes to facilitate the effective transfer and exchange of information to the 'right' individuals at the 'right' time. One of the primary objectives of care coordination was the effective transfer of information to inform patient management and decision-making. This was achieved via formal

For peer review only

mechanisms including multidisciplinary team meetings, ward rounds and documentation, shared information systems, and informal and spontaneous communication.

Multidisciplinary team meetings in hospitals took a range of forms including trauma meetings of the trauma healthcare team, 'board rounds' (reviews of ward patients' progress in relation to discharge), 'huddles' (short briefings between team members), and discharge planning meetings (to plan for patients leaving hospital). Participants identified characteristics of successful multidisciplinary team meetings including establishing patient goals, regular attendance by a range of specialties, and effective management such as time management and documentation of outputs. However, these were threatened by lack of attendance from key professional groups due to competing commitments and a view that attendance was "a waste of time" as time would be better spent with patients, or because meetings often repeated issues without resolution.

Multidisciplinary team documentation was vital as a means to transfer patient information. However, patient documentation was not always accessible to relevant teams at key points in the care pathway, nor appropriately designed or consistently completed to capture all relevant information. Information transfer was facilitated through patient tracking systems, 'ward boards' displaying key patient information, and electronic patient records. Communication processes were undermined when IT systems did not cross boundaries either within secondary care, or with external organisations such as primary care and community services, including when primary care records could not be accessed.

Multidisciplinary team ward rounds and 'huddles' provided opportunities to create shared

For peer review only

knowledge and problem-solve. Participants advocated varied modes of communication to bring together professional groups at different stages of the care pathway.

Shared workspaces promoted frequent and spontaneous verbal communication. Allocating patients to specific hip fracture wards facilitated this exchange as healthcare professionals were concentrated together. Spontaneous communication was also achieved using technologies such as WhatsApp, mobile 'phone and email. However, success depended on the interpersonal skills of staff. Verbal communication was hindered by hierarchical relationships. While some senior members of staff emphasised lack of hierarchy on wards, one Advanced Nurse Practitioner (ANP) felt that ward staff were "wary" of discussing patient management with consultants. They felt this was due to ward staff lacking confidence, coupled with the belief in persistence of traditional hierarchy. The ANP therefore acted as a go-between while also encouraging ward staff to approach and communicate directly with consultants. 'Coordinators' functioned as key points of contact, providing continuity and daily communication between professional groups at different stages of the care pathway. Formal, named coordinator roles included trauma and discharge coordinators and hip fracture nurse specialists. Informal coordinating roles provided teams with an additional point of contact. In one site this included a named member of staff with responsibility for hip fracture patients. However, over reliance on individuals meant that when they were away, communication and coordination was compromised.

3. Shared goals

Proposition 3: Successful multidisciplinary teamwork depends on the existence of a shared

For peer review only

and well-defined purpose within the team.

Shared goals were undermined by complex dynamics between different professional groups, particularly geriatric and orthopaedic staff who participants felt had different priorities. Several orthogeriatricians described orthopaedics as a specialty that focused on “fixing” the fracture, compared with their holistic approach. The structural divisions in NHS Trusts that placed staff within different ‘directorates’ were seen as reinforcing professional differences and impeding communication. Engagement with shared goals was also challenging due to staff transience, particularly of junior doctors.

Shared goals were encouraged through multiple processes. Daily multidisciplinary team meetings enabled staff to build shared goals and collaborative treatment plans for individual patients and to hear “different points of view”. Multidisciplinary team training provided opportunities for staff to share knowledge and understanding. This was particularly evident in the training, mentoring and support for junior staff. Clinical leads were integral to bridging professional boundaries and creating a common professional identity across individual specialties. They did this by establishing shared priorities and goals in multidisciplinary team planning meetings, sharing monitoring and auditing results with colleagues across the care pathway, and using audit findings to drive service improvement. Communal efforts in monitoring and appraisal provided further opportunities to reinforce shared priorities and goals, such as reviewing National Hip Fracture Database data—a national audit program to benchmark services, mortality events, internal audits and informal discussions about service improvement.

For peer review only

4. Collaborative leadership

Proposition 4: Successful multidisciplinary teamwork depends on collaborative leadership across several specialties and levels.

Successful multidisciplinary teamwork relied on presence of nominated departmental leads for hip fracture care. These formal clinical leads had a facilitative and motivational role and centralised hospital leadership created an environment to support collaboration. Participants advocated a formal model of leadership that was concentrated in small, mixed specialty teams across orthopaedic, orthogeriatric, aesthetic and nursing divisions. Participants identified specialties missing at this level, including anaesthetists. Therapy staff felt excluded at this level and thought that their representation could promote more effective collaboration. Senior level leadership was thought to promote commitment among colleagues at all levels.

Clinical service leads had a facilitative and motivational role in engaging healthcare professionals from a range of specialties and assembled teams to develop shared priorities and goals, joint protocols, audit priorities and mutually-owned quality improvement plans. Multidisciplinary team planning meetings were a key part of this process. Participants identified several attributes that enabled 'heroic' individuals to fulfil these roles, such as "passion" and "drive". The visibility of clinical service leads within the service and respect for their clinical judgement legitimised their position and influenced their success in achieving these aims. Hospitals with one strong clinical service lead emphasised the importance of leadership development and succession planning to maintain team stability and

For peer review only

performance.

Effective leadership on the wards depended on centralised leadership that created an environment to support collaboration. Support included providing sufficient capacity and resources for hip fracture care including capacity for staff to attend MDT meetings, supporting local leads to engage in service planning and training by building this into job plans and supporting service improvement initiatives and innovation to help provide a sense of shared purpose. Some participants expressed frustration that reimbursement from best practice tariffs for hip fracture care was not sufficiently reinvested into service development. Participants emphasised the need for designated hospital board executives who engaged in bidirectional communication with clinical service leads and championed hip fracture care to facilitate this. Several participants highlighted a lack of centralized leadership due to the transience of higher management roles and competing priorities within the Trust.

For peer review only

Table 2: Core components of successful multidisciplinary working for hip fracture care: illustrative quotations

**Defined roles and responsibilities**<br>*Proposition 1:* Successful multidisciplinary teamwork depends on clarity in roles and responsibilities within the team	
**Themes identified**	**Illustrative quotations (all names are pseudonyms)**
Effective use of hip fracture protocols and formalised care pathways<br><br>‘Buy in’ of protocol and pathway documents<br><br>Comprehensive induction on protocols and pathways<br><br>Clear job planning to outline parameters of	“We have a good communication right from the start [of the pathway] … it’s very kind of clear cut whose responsibility is what.” Sue, Band 8 advanced majors’ practitioner, emergency department, Radford<br><br>“Not many people know what OT [occupational therapy] is and you’ve really got to fight sometimes about your professional role in things…. It’s that you’re just having to keep fighting, ‘No, that’s not for us. That’s for somebody

professional roles	else'." Lois, trauma and orthopaedic ward occupational therapist, Newbridge<br><br>"There was a complete lack of understanding from the trauma and orthopaedic consultants as to an expectation from them that orthogeriatrics was my biggest priority. And I was saying, well it's not in my job plan to do it and there was a complete lack of understanding from them about that." Elaine, consultant geriatrician, Newbridge<br><br>"Well I think the protocols and pathways work really well if everybody adheres to it, but with having a large department with a variety of clinicians involved it's difficult to get a buy-in from everybody." Peter, consultant orthopaedic surgeon , Radford<br><br>"Trying to get everybody to sign up to say something will always happen, seems to be quite difficult at the moment.... It does just take time and the people who have recently come through training tend to be far more cohesive and wanting to have demonstrable pathways that can be audited and guide people who are more junior." Tania, consultant anaesthetist, Newbridge
**Information transfer processes**<br>*Proposition 2:* Successful multidisciplinary teamwork depends on processes to facilitate the effective transfer and exchange of information to the 'right' individuals at the 'right' time.	
**Themes identified**	**Illustrative quotations (all names are pseudonyms)**
Use of a range of multi-disciplinary team meetings forms including trauma meetings, focused 'board rounds', 'huddles', and discharge planning meetings<br><br>Effective multidisciplinary team meetings including establishing patient goals, regular attendance by a range of specialties and effective management<br><br>Accessible multidisciplinary team documentation that is completed consistently and appropriately designed<br><br>Shared information systems that cross organisational boundaries<br><br>Multidisciplinary team ward rounds to create shared knowledge and problem-solve<br><br>Shared workspaces to promote verbal communication, including placing patients on a designated hip fracture ward<br><br>Spontaneous communication using technologies	"I'm involved in the daily trauma meetings every morning, so that is all the consultants, that's the service manager, that's the junior doctors and that's us as an ACP [Advanced Clinical Practice] team. And you talk about the patients.... Sometimes the consultants can talk over you and forget that you're actually telling them what they need to know." Jacqui, advanced nurse practitioner, Maplegrove<br><br>"I've got a bleep, they've got my mobile, so I'm contactable throughout the day. So people from wards will be ringing me to ask questions." Joanne, trauma coordinator, Newbridge<br><br>"I think sometimes that probably when some of the consultants or the anaesthetists come on the ward that sometimes [ward staff are] a bit wary of approaching them." Barbara, advanced nurse practitioner, Maplegrove<br><br>[The trauma coordinator] role has sort of, it's found itself floundering a bit so we don't intend to remove it, we just intend to enhance it and make it more clear what the role [is]. Darren, service manager, Radford

For peer review only

such as WhatsApp, mobile ‘phone and email<br><br>Use of named coordinators as key points of contact, including trauma and discharge coordinators and hip fracture nurse specialists<br><br>Use of informal coordinators as points of contact	
**Shared goals**<br>*Proposition 3:* Successful multidisciplinary teamwork depends on the existence of a shared and well-defined purpose within the team	
**Themes identified**	**Illustrative quotations (all names are pseudonyms)**
Importance of dynamics between different professional groups<br><br>Impact of structural divisions within NHS Trusts that reinforce professional differences<br><br>Staff transience undermining shared goals<br><br>Use of daily multidisciplinary team meetings to build shared goals<br><br>Multidisciplinary team training to share knowledge and understanding<br><br>Importance of clinical leads in bridging professional boundaries<br><br>Communal efforts in monitoring and appraisal	“Essentially, I think we all are bound by sort of evidenced based [care], I think that is a common sort of thing and we all want the best for our patients. And I think those are two things which bind us that we all want to do the right thing for the patients.” Dheeraj, consultant anaesthetist, Radford<br><br>“It’s one of our major battles [as orthogeriatricians], the fact that we struggle to engage [with orthopaedic surgeons]. We struggle with them to engage with us, because you know basically they fix them and then walk away but occasionally we need them back and it’s an ongoing battle, always has been.” David, orthogeriatric advanced nurse specialist, Springhill<br><br>“I’m not a fan of the divisional structure of hospitals. I think it’s quite artificial and hospitals are trying too hard to align themselves like a corporation would… I think it can [impede communication].” Andy, consultant orthopaedic surgeon, Maplegrove<br><br>“Our top consultant that works on [the hip fracture] ward, she’s very driven by being the best, and having the best outcome for our patients. So I think everyone has a bit of a collective drive, but I think the pathway, being on that ward and on the pathway is a mega drive. Because it’s constantly evaluated.” Jane, occupational therapist, Springhill<br><br>“Updating your team is massively important. If you explain to your team why you're doing something, what you'd like done and why you'd like it done, that really helps. It's just that you've got that shared aim at the end of it. If you're working towards the shared aim, I think things work much better.” Lucy, emergency medicine consultant, Springhill
**Collaborative leadership**<br>*Proposition 4:* Successful multidisciplinary teamwork depends on effective, collaborative leadership across several specialties and levels	

For peer review only

Themes identified	Illustrative quotations (all names are pseudonyms)
Importance of nominated departmental leads for hip fracture care<br><br>Formal model of leadership in small, mixed speciality teams<br><br>Clinical service leads with a facilitative and motivational role<br><br>Use of multidisciplinary team planning meetings<br><br>Importance of leadership development and succession planning to maintain stability<br><br>Centralised leadership providing sufficient capacity and resources for hip fracture care<br><br>Support for service improvement initiatives and innovation<br><br>Designated hospital board executives who engage with clinical service leads and champion hip fracture care	"I mean I think there is good engagement in the service at a senior level, and for that reason I think that focus, that sort of filters down across other members of the team." Kate, consultant geriatrician, Springhill<br><br>"I think if you get a core of people who are interested and enthusiastic, and I think that's across all disciplines, then there's no doubt that commitment can then drive improvements in other areas." Jon, consultant anaesthetist, Radford<br><br>"You've got to find key people [from a range of specialties] that are passionate about [hip fracture care] and get it. And even understand that, you know, the frailest of the frail, and you've got to do it properly and that takes lots of different services and lots of different professionals to pull that together … I guess I've been lucky here because people have put their head above the parapet rather than me pulling them in sort of kicking and screaming." Amanda, consultant orthogeriatrician, Springhill<br><br>"So, I think what worked well initially was a lot of buy-in and support from management. So three, four years ago one of the senior business unit managers, called in a couple of consultants that said, 'I'll support whatever you want to help this pathway better, what can we do about it?'… I think [it's helpful because] it brings the whole team together." Peter, consultant orthopaedic surgeon, Radford

5. Underpinning concept: Shared responsibility (Table 3)

Proposition 5: Roles and responsibilities, information transfer processes, shared goals and collaborative leadership all depend on a culture of shared responsibility for care delivery.

Underpinning and crossing all components, shared responsibility was evident and articulated. A culture of shared responsibility for hip fracture care ensured all members of the multidisciplinary team were invested in care delivery. This was enabled through care models where patients were cared for jointly by orthopaedic surgeons and

For peer review only

orthogeriatricians. Staff valued the assistance of colleagues in performing tasks when needed, particularly when they were overloaded. Shared responsibility was also evidenced by colleagues providing feedback to improve performance and taking on supervisory roles. Orthogeriatricians were seen as occupying a key role in supervision of and support for advanced nurse practitioners, therapists and junior doctors (medical and surgical). There was evidence that traditional role boundaries were blurred. This included the involvement of nurses in mobilisation of patients when therapists were not available, and anesthetists' work to upskill emergency department doctors in nerve block provision.

Mutual trust and respect for one another's skills created an environment that encouraged staff to participate actively in the multidisciplinary team including in meetings, discussions with colleagues, and constructive management of disagreements. Trust and respect were based on a recognition of the value of different professional groups. Familiarity with colleagues, both professionally and personally, helped to build this trust. Informal relationships were facilitated through shared workspaces. Mutual trust and respect were often cultivated over time.

Table 3: Underpinning concept of shared responsibility: illustrative quotations

**Underpinning concept: Shared responsibility**<br>*Proposition 5:* Successful multidisciplinary teamwork depends on a culture of shared responsibility for care delivery	
**Themes identified**	**Illustrative quotations (all names are pseudonyms)**
Use of joint care models between orthopaedic surgeons and orthogeriatricians<br><br>Assistance from colleagues in performing tasks when needed	"When we're all talking together [as a team] sometimes we can go, 'Can you do this and we'll do that' and I suppose it's just supporting each other…. So again, sometimes it's just like, 'If you do this then that gives me time to do that' and we work that way." Jessica, occupational therapist, Radford

For peer review only

Provision of feedback to improve performance<br><br>Blurring of traditional role boundaries<br><br>Mutual trust and respect for one another’s skills<br><br>Recognition of the value of different professional groups<br><br>Familiarity with colleagues to build trust	“You’ve got to listen to everybody and without certain people in that team it won’t work, so I form a good rapport with the social workers, placement of care hub, so that you know they can trust me and I can trust them.” Alice, foundation year 2 doctor, Maplegrove<br><br>I think you need to appreciate each other in terms of what people bring. You need to recognise other people’s strengths and whether they’re best placed to talk about something or you are yourself. Andy, consultant orthopaedic surgeon, Maplegrove<br><br>“I think there has to be an element of respect [for colleagues from different teams] as well, that in the good old days, a consultant was king and the staff were just, you know the minions, working minions, working around them but nowadays there's more of an equality in the relationship. So, I would probably quite happily give an opinion [to] the Nurse on the ward, if she has a query about a patient, cause she sees the patient every day, so there's quite an open communication both ways.” Lance, consultant orthopaedic surgeon, Radford<br><br>“At one point I did start going to the trauma meeting for a bit. And actually that was really interesting for me because I did realise, hearing them talk about how specialised their knowledge was actually about operations and the operative management of problems. And I found that really interesting because I thought gosh, you know, these people do know a lot about what they did.” Elaine, consultant geriatrician, Newbridge

Discussion

This study has characterised how hospitals deliver multidisciplinary work for hip fracture care and suggests core components needed to deliver this. Findings identified four components to promote successful multidisciplinary teamwork: 1) defined roles and responsibilities, 2) information transfer processes, 3) shared goals, and 4) collaborative leadership. Shared responsibility underpinned and acted across all four components.

For peer review only

Relationship to current literature

This study complements existing work on optimal care models for hip fracture care by exploring how multidisciplinary teamwork can be organised and delivered in practice. Guidelines and existing research have highlighted the importance of implementing formal processes to coordinate care, including use of well-defined care pathways and protocols[5, 37] and good communication to facilitate information transfer[9]. The alignment of team goals through multi-disciplinary and clinical governance meetings and shared learning has also been identified within the acute rehabilitation of hip fracture patients. National Institute for Health and Care (NICE) implementation advice for hip fracture management and British Orthopaedic Association (BOA) guidelines have highlighted the importance of identifying an overarching clinical lead for hip fracture care, supported by senior leads from a range of specialties[9]. Coordination between orthogeriatricians and orthopaedic surgeons[38, 39] and formal roles such as trauma and discharge coordinators have been highlighted elsewhere as important [40, 41, 42].

Our findings also highlight possible barriers to effective multidisciplinary teamwork. These included some reluctance by more experienced staff to use hip fracture pathway documents or newly developed protocols, as well as barriers to communication presented by belief in hierarchy. Findings reflect wider systemic issues that have been identified in the context of other multidisciplinary teams in the NHS. These include the existence of structural arrangements within and between secondary care and external organisations that hinder communication, such as incompatible computing systems[43], staff transience[17], and steep hierarchies that stymie communication within teams [44]. The socialisation of staff

For peer review only

into different professional groups with distinct cultures, identities and priorities has further been identified as a hindrance to collaborative work[45]. The model of clinical service leadership described here reflects a broader trend in healthcare away from a 'command and control' approach to a motivational and facilitative style that involves building relationships with staff from a range of specialties[46]. It also reflects increasing recognition of the value of collaborative leadership in which leadership roles are shared between team members[47].

Hip fracture care includes separate roles with some evidence of interdisciplinary working through blurring of traditional role boundaries[19]. Core components of teamwork in this context encompass a range of skills (e.g., leadership), processes (e.g., hip fracture protocols and pathways, formal mechanisms for information transfer), and values (e.g., shared goals)[17]. Several strategies to support multidisciplinary teamwork are reflected in previous work, including interdisciplinary training[48], shared monitoring and evaluation processes[17] and familiarisation with colleagues to build trust[21].

Our study has identified the centrality of shared responsibility within multidisciplinary teams delivering care for hip fracture. Care processes such as multidisciplinary team meetings, monitoring and auditing processes and multidisciplinary team training can serve dual purposes by creating and sustaining shared responsibility as a value.

This work is taking place within a broader programme to develop a toolkit to enable service providers and commissioners to improve organisation and delivery of hip fracture services[27], results due in 2023. Based on our findings, we have developed our model into

For peer review only

a how to guide of practical recommendations to for hip fracture care, including interdisciplinary 'exchange' training built into both orthopaedic and orthogeriatrics speciality training curricula (Supplementary material). This resonates with work focused on other conditions, including cancer, that require multidisciplinary teamwork[22, 49].

Supplementary material: A how to guide, a multidisciplinary toolkit for hip fracture care

Strengths and limitations

The sample, comprising 40 participants drawn from four UK hospitals, provided sufficient data to address the research area and included professionals with relevant experience, as such the study achieved information power[30]. Using an abductive analysis approach, rather than transposing data onto an existing theory or framework, ensured the results were data-driven such that data were not forced into pre-defined constructs[34]. Accordingly, where relevant, data were coded into more than one concept. We invited all healthcare professionals who working within the service to participate. However, it is possible that those who declined were less engaged in service delivery; we also note that others involved in care or discharge planning—such as social workers—did not take part and that numbers of allied health professionals (7 out of 40) were relatively low compared with their overall representation in trauma care. Despite these limitations, the study included participants from diverse professional backgrounds, and the candid way that they spoke about challenges provided us with confidence in the veracity of the data. As data collection took place in hospitals that admitted moderate to large numbers of hip fracture patients, the transferability of findings to smaller units may be limited and there may be need for

For peer review only

more work to understand fracture care in such settings[50].

Further research

Further work is needed to understand leadership configurations in hip fracture care, particularly the role of ‘emergent’ leaders who do not have formal roles but exert considerable influence within teams[51]. Future research is also needed in smaller contexts and we are planning to evaluate the impact of our toolkit on the organisation and delivery of hip fracture services.

Conclusions

This study used qualitative methods to develop a description of core components needed to deliver multidisciplinary teamwork for hip fracture care. It highlights the importance of creating and maintaining a culture that includes shared responsibility for care delivery. Our findings provide a basis for future training, skills development and assessment of multidisciplinary teamwork for hip fracture care in the UK.

Acknowledgements

We thank all the healthcare professionals who participated in this study and the REDUCE study group who provided support and guidance. The REDUCE study group consists of Dr Rita Patel, Professor Andrew Judge, Professor Antony Johansen, Dr Elsa M R Marques, Dr Petra Baji, Ms Jill Griffin, Ms Marianne Bradshaw, Dr Katie Whale, Mr Tim Chesser, Professor

For peer review only

Xavier L Griffin, Dr Muhammad K Javaid and Professor Yoav Ben-Shlomo. We are grateful to members of the Patient Experience in Research Partnership (PEP-R), whose input shaped the research design and how it was carried out.

Author contributions

All authors contributed to the design of this research and contributed to the acquisition, analysis and/or interpretation of data. All authors contributed to drafting this work and revising it for important intellectual content, and all gave final approval for the version to be submitted. The authors comprise two social scientists with several years of experience in health research (SD, FF), a practicing consultant orthogeriatrician with academic expertise in epidemiology and population health research (CG), and a social anthropologist with expertise in qualitative and interdisciplinary research (RGH). We brought our different backgrounds into the research including in its design and conduct, including in interpretation of the data and results.

Competing interests

Authors have no competing interests to declare.

Funding

This work was supported by Versus Arthritis (reference 22086).

For peer review only

Data sharing statement

Anonymised interview data may be accessed via the University of Bristol Research Data Repository. Access to the data will be made available to researchers for ethically approved research projects, from a six-year embargo period, which ends on 1st April 2029. Data can be accessed on the understanding that confidentiality will be maintained and after a Data Access Agreement has been signed by an institutional signatory. No authentic request for access will be refused. Data are available at the University of Bristol data repository, data.bris, at https://doi.org/10.5523/bris.3f9j9z626s7f32a3r5j1wd7zt7.

Ethics approval

Ethical approval was provided by the Faculty of Health Sciences University of Bristol Research Ethics Committee (Ref: 108284) and by the NHS Health Research Authority (20/HRA/71). NHS settings (Trusts) for the four study sites each provided research governance approval.

Figure legend

Figure 1: Core components of successful multidisciplinary teamwork for hip fracture care

References

1. Gullberg, B., O. Johnell, and J.A. Kanis, *World-wide projections for hip fracture.* Osteoporos Int, 1997. **7**(5): p. 407-13.

For peer review only

2. Neuburger, J., et al., *The Impact of a National Clinician-led Audit Initiative on Care and Mortality after Hip Fracture in England: An External Evaluation using Time Trends in Nonaudit Data.* Medical Care, 2015. **53**(8): p. 686-691.
3. Griffin, X.L., et al., *Recovery of health-related quality of life in a United Kingdom hip fracture population. The Warwick Hip Trauma Evaluation--a prospective cohort study.* Bone Joint J,, 2015. **97-b**(3): p. 372-82.
4. Judge A, et al., *Models of care for the delivery of secondary fracture prevention after hip fracture: a health service cost, clinical outcomes and cost-effectiveness study within the South Central Region. National Institute for Health Research (NIHR), Health Services and Delivery Research (HS&DR)*. 2016.
5. Kates, S.L., *Hip fracture programs: are they effective?* Injury, 2016. **47 Suppl 1**: p. S25-7.
6. National Institute for Health and Care Excellence (NICE), *Hip fracture in adults: Quality standard [QS16]*. 2017, Manchester: NICE.
7. National Institute for Health and Care Excellence (NICE), *Hip fracture: management: Clinical guideline [CG124]*. 2017: Manchester, NICE.
8. National Osteoporosis Society, *Effective secondary prevention of fragility fractures: clinical standards for fracture liaison services*. 2015: Bath: NOS.
9. Association, B.O., *BOAST 1 version 2-patients sustaining a fragility hip fracture.* London. British Orthopaedic Association, 2012.
10. Falls and Fragility Fracture Audit Programme (FFFAP), *National Hip Fracture Database (NHFD) annual report*. 2021: Royal College of Physicians, London.
11. *The care of patients with fragility fracture*. 2007: British Orthopaedic Association.
12. Christie, J., et al., *Improving the experience of hip fracture care: A multidisciplinary*

For peer review only

collaborative approach to implementing evidence-based, person-centred practice.

13. Hordvik, H.B., et al., *Physiotherapists´ experiences with older adults´ rehabilitation trajectory after hip fracture: A qualitative study in Western Norway.* Physiotherapy Theory and Practice, 2023. **39**(2): p. 328-342.
14. Guerra, S., et al., *Multidisciplinary team healthcare professionals' perceptions of current and optimal acute rehabilitation, a hip fracture example A UK qualitative interview study informed by the Theoretical Domains Framework.* PLoS One, 2022. **17**(11): p. e0277986.
15. Willcocks, S.G., *Exploring team working and shared leadership in multi-disciplinary cancer care.* Leadersh Health Serv (Bradf Engl), 2018. **31**(1): p. 98-109.
16. Grand, J.H., S. Caspar, and S.W. Macdonald, *Clinical features and multidisciplinary approaches to dementia care.* Journal of multidisciplinary healthcare, 2011. **4**: p. 125-147.
17. Ellis, G. and N. Sevdalis, *Understanding and improving multidisciplinary team working in geriatric medicine.* Age and Ageing, 2019. **48**(4): p. 498-505.
18. Salas, E., D.E. Sims, and C.S. Burke, *Is there a "Big Five" in Teamwork?* Small Group Research, 2005. **36**(5): p. 555-599.
19. D'Amour, D., et al., *The conceptual basis for interprofessional collaboration: core concepts and theoretical frameworks.* J Interprof Care, 2005. **19 Suppl 1**: p. 116-31.
20. Lemieux-Charles, L. and W.L. McGuire, *What Do We Know about Health Care Team Effectiveness? A Review of the Literature.* Medical Care Research and Review, 2006. **63**(3): p. 263-300.
21. D'Amour, D., et al., *A model and typology of collaboration between professionals in healthcare organizations.* BMC Health Serv Res, 2008. **8**: p. 188.

For peer review only

22. Rosen, M.A., et al., *Teamwork in healthcare: Key discoveries enabling safer, high-quality care.* The American psychologist, 2018. **73**(4): p. 4 Nilsen -450.

23. Soukup, T., et al., *Successful strategies in implementing a multidisciplinary team working in the care of patients with cancer: an overview and synthesis of the available literature.* J Multidiscip Healthc, 2018. **11**: p. 49-61.

24. Salas, E., D.L. Reyes, and S.H. McDaniel, *The science of teamwork: Progress, reflections, and the road ahead.* Am Psychol, 2018. **73**(4): p. 593-600.

25. Bélanger, E. and C. Rodríguez, *More than the sum of its parts? A qualitative research synthesis on multi-disciplinary primary care teams.* J Interprof Care, 2008. **22**(6): p. 587-97.

26. Coyne, I.T., *Sampling in qualitative research. Purposeful and theoretical sampling; merging or clear boundaries?* J Adv Nurs, 1997. **26**(3): p. 623-30.

27. Patel, R., et al., *REducing unwarranted variation in the Delivery of high qUality hip fracture services in England and Wales (REDUCE): protocol for a mixed-methods study.* BMJ Open, 2021. **11**(5): p. e049763.

28. Baker, S.E. and R. Edwards, *How many qualitative interviews is enough.* 2012.

29. *Snowball sampling*, in *The SAGE encyclopedia of social science research methods*, M.S. Lewis- Beck, Bryman, A., & Futing Liao, T. , Editor. 2004, Sage Publications, Inc.: Thousand Oaks, CA.

30. Malterud, K., V.D. Siersma, and A.D. Guassora, *Sample Size in Qualitative Interview Studies: Guided by Information Power.* Qual Health Res, 2016. **26**(13): p. 1753-1760.

31. *Qualitative Researching with Text, Image and Sound*. 2000: London.

32. Tavory, I. and S. Timmermans, *Abductive analysis: Theorizing qualitative research*. 2014: University of Chicago Press.

For peer review only

33. Nilsen, P., *Making sense of implementation theories, models and frameworks.* Implementation Science, 2015. **10**(1): p. 53.

34. Edwards-Jones, A., *Qualitative data analysis with NVIVO*. 2014, Taylor & Francis.

35. Pope, C., S. Ziebland, and N. Mays, *Qualitative research in health care. Analysing qualitative data.* Bmj, 2000. **320**(7227): p. 114-6.

36. Gooberman-Hill, R., et al., *Involving patients in research: considering good practice.* Musculoskeletal Care, 2013. **11**(4): p. 187-90.

37. Meinberg, E., et al., *Hospital-based Hip fracture programs: Clinical need and effectiveness.* Injury, 2020. **51**: p. S2-S4.

38. Fernandez, M.A., X.L. Griffin, and M.L. Costa, *Management of hip fracture.* British Medical Bulletin, 2015. **115**(1): p. 165-172.

39. Chudyk, A.M., et al., *Systematic Review of Hip Fracture Rehabilitation Practices in the Elderly.* Archives of Physical Medicine and Rehabilitation, 2009. **90**(2): p. 246-262.

40. Riemen, A.H.K. and J.D. Hutchison, *The multidisciplinary management of hip fractures in older patients.* Orthopaedics and trauma, 2016. **30**(2): p. 117-122.

41. (UK), N.I.f.H.a.C.E., *Major Trauma: Service Delivery NICE Guideline NG40*. 2016.

42. (NICE), N.I.f.H.a.C.E., *Implementation advice; Implementing the Hip Fracture Programme*. 2012, NICE: Manchester.

43. Zhang, J., et al., *Interoperability in NHS hospitals must be improved: the Care Quality Commission should be a key actor in this process.* Journal of the Royal Society of Medicine, 2020. **113**(3): p. 101-104.

44. Green, B., et al., *Challenging hierarchy in healthcare teams – ways to flatten gradients to improve teamwork and patient care.* British Journal of Oral and Maxillofacial Surgery, 2017. **55**(5): p. 449-453.

For peer review only

45. Powell, A.E. and H.T.O. Davies, *The struggle to improve patient care in the face of professional boundaries.* Social Science & Medicine, 2012. **75**(5): p. 807-814.

46. Storey, J. and R. Holti, *Towards a New Model of Leadership for the NHS.* 2013.

47. Günzel-Jensen, F., A.K. Jain, and A.M. Kjeldsen, *Distributed leadership in health care: The role of formal leadership styles and organizational efficacy.* Leadership, 2016. **14**(1): p. 110-133.

48. Reeves, S., et al., *Interprofessional education: effects on professional practice and healthcare outcomes.* Cochrane Database of Systematic Reviews, 2013(3).

49. *The Characteristics of an Effective Multidisciplinary Team (MDT)*. 2010; Available from: file:///C:/Users/sd17432/Downloads/NCATMDTCharacteristics_FINAL.pdf.

50. Forero, R., et al., *Application of four-dimension criteria to assess rigour of qualitative research in emergency medicine.* BMC Health Services Research, 2018. **18**(1): p. 120.

51. Hanna, A.A., et al., *The Emergence of Emergent Leadership: A Comprehensive Framework and Directions for Future Research.* Journal of Management, 2020. **47**(1): p. 76-104.

For peer review only

Figure 1: Core components of successful multidisciplinary teamwork for hip fracture care

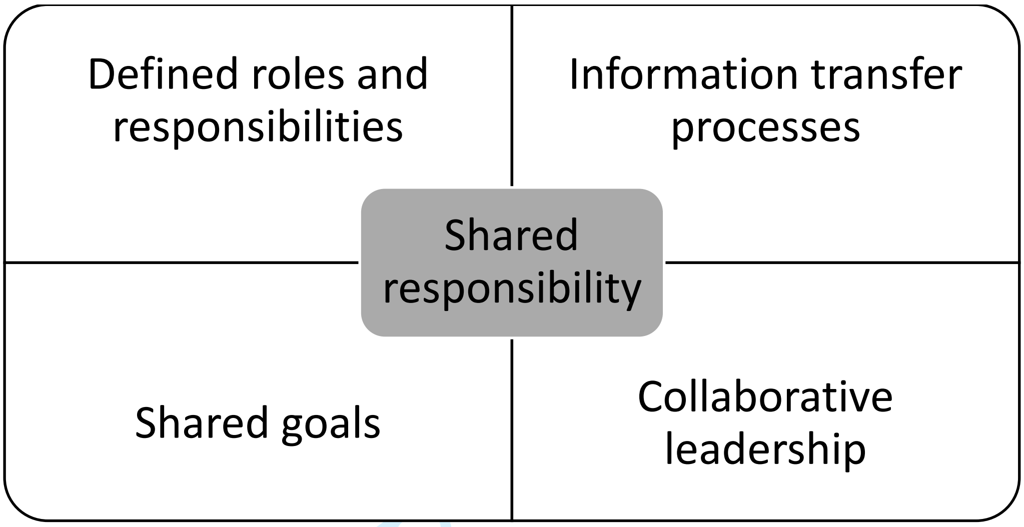

For peer review only

Supplementary material: Interview topic guide

Aims and objectives

- To investigate how hip fracture services are provided in different hospital settings and factors that help/ hinder ability to deliver care
- This study will provide us with information that can be used to improve the delivery of hip fracture services and reduce variation in care

Topics covered will be:

- Find out a bit about you
- Organisation of the care pathway
- Barriers and facilitators to service delivery
- Close

For peer review only

Part 1: About you

- Job title
- Part time/ full time/ %FTE
- Professional background (years since qualifying and progression)
- Years since qualifying for this role
- Years spent working in this service
- Age
- Date of birth
- Gender identify with
- Describe own role in delivering hip fracture care (before Covid)
  - Face to face duties
  - Administrative / organisational duties
  - Management duties

Part 2: Care pathway before Covid-19

- Things that worked well/ not so well/ improvements.

  Examples of components of care

  - Ambulance hand over
  - Emergency department factors [NB: Fast-tracking to ward]
  - Imaging services
  - Orthogeriatric services
  - Pre-operative care
  - Theatre and anaesthetic services
  - Orthopaedic & Trauma Services
  - Inpatient physiotherapy services and rehabilitation
  - Training/ confidence in the manual handling of patients
  - Links to dementia care, mental health services, diabetes care, dietetics, palliative care
  - Discharge systems and links to intermediate care
  - Temporary rehabilitation / care home provision
  - Post-discharge queries from patients
  - Re-admission processes for potential complications
  - Fracture Liaison Services/ secondary fracture prevention pathways
  - Falls services
  - Patient information and education
  - Community physiotherapy services and rehabilitation
  - Relationships with care homes – infection control protocols for new placements and return of patients to their original care homes

For peer review only

Part 3: Barriers and facilitators to service delivery before COVID-19

Things that worked well/ not so well/ improvements:

Communication

- How well did everyone communicate within service?
    - Within team
    - Other colleagues in MDT
    - Links with other services, e.g. dementia care, mental health services, diabetes care, palliative care
    - Community services and rehabilitation

  [Probes: MDT/ morbidity and mortality meetings, trauma rounds, multi-disciplinary paperwork, shared protocols, shared governance meetings, central coordinator]

  - What worked well/ not so well/ improvements

- Department management/ senior management

  - What worked well/ not so well/ improvements

- External organisations
    - Primary care
    - Community services and rehabilitation
    - Social care services

Relationships

- What does a 'good' working relationship mean to you?
- How well did everyone work together
    - Within team
    - Other colleagues in MDT
    - Department management/ senior management
- What sorts of things contributed to good working relationship with colleagues/

made it more difficult?

- Improvements

Commitment to delivering care

- To what extent did your colleagues do what was expected of them?

  [Probes: Extent to which the 36 hour target for hip fracture operation was pushed, extent individuals adhered to protocols]

  - Why do you think this was?

- How engaged/ enthusiastic do you think your colleagues were?

  - What helped/ made it more difficult?

Access to resources

- To what extent did you have access to all the resources needed to carry out work?

  [Probes: staff vacancies, theatre capacity, surgeon capacity, workload and material resources, access to medical and social care data],

  - If not, why not?
  - What did you do to address this challenge?

- To what extent did you/ team have access to the training and information needed to carry out work?

  [Probes: training, evidence base, understanding what is required of them]

- Training/ mentorship programmes in place
- What worked well/ not so well/ improvements
- How much of a priority do you feel that hip fracture care was for the?

  - Hospital Trust
  - Department
  - Boss

  - Why/ why not priority?

For peer review only

- Strategies to engage senior management

Service delivery over time

- Example quality improvement project
    - How did change occur?
- Example when you tried to initiate change but couldn't
    - Why?
- What monitoring and evaluation processes are in place?

[Probes: national initiatives, internal audit and governance processes, review of cases in morbidity and mortality meetings]

- What is the impact of these monitoring and evaluation processes on service delivery?

[Probes: Are these fed back to staff, do they effect change]

- Example of local clinical audit – why initiated?
- What clinical governance activities are in place for hip fracture care?

[Probes: risk management, transparency, quality improvement, mortality reviews, strategies to review clinical incidents/ complaints]

How useful are they?

Part 4: Close

1. If you could change one thing about your (pre-Covid) service, what would it be?
2. Are there any other things or insights that you would like to tell me about today?
3. Do you have any questions for me about the project?
4. Thank them
5. Reaffirm confidentiality

For peer review only

For peer review only

For peer review only

Supplementary material: A 'how to' guide for effective multi-disciplinary team working in hip fracture care

Delivering hip fracture care requires multi-disciplinary teamwork, including effective communication and coordination across a range of specialties and professional roles including emergency medicine, orthopaedic surgery, anaesthetics, geriatric medicine, rehabilitation and nurses. The range of the multidisciplinary team involved reflects the complex needs of patients with hip fracture. Clinical guidelines recommend timely hip fracture care, with when needed, rapid transfers of care between professionals. Streamlining multi-disciplinary care is crucial to the delivery of safe and effective patient care.

There are four components needed for coordinated multi-disciplinary team (MDT) working. These are all underpinned by one central concept: shared responsibility. The responsibility for delivering these components lies with *all members* of the MDT.

Action 1: Clearly define roles and responsibilities of each member of the MDT

Roles and responsibilities are more than what is simply listed in a job description. Each member of the MDT needs to understand the other MDT member's roles and responsibilities. Hence, roles and responsibilities need to be appreciated and explained during induction processes.

Roles and responsibilities need to be co-agreed between an individuals' line manager, within the MDT and outlined in personalised job plans. These job plans should be reviewed at annual appraisals with modifications made according to MDT developments/ progress.

The clinical lead needs to have oversight of all roles and responsibilities in order to identify gaps in the pathway of hip fracture care, so as to manage the skillset within the MDT to ensure those gaps are filled.

Action 2: Set up systems to ensure clear, timely MDT communication

Hip fracture patients have complex medical and surgical needs and are often acutely unstable. Therefore, MDT communication must be dynamic and efficient with the capacity for immediate response from senior team members when necessary. Systems are enhanced by the presence of trauma coordinators and discharge coordinators with defined roles and responsibilities.

Face-to-face communication events (verbal)
Systems need to be established for effective communication events with those attending pre-agreed. Communication events should begin promptly, using the same standardised

For peer review only - http://bmjopen.bmj.com/site/about/guidelines.xhtml

format each time. Job plans need to permit attendance. Examples of communication events include daily trauma meetings, early morning board rounds ('huddles') and pre-arranged discharge planning meetings. Short meetings are more efficiently conducted whilst standing, where ability permits.

Six principles of effective MDT communication events include:

1. Ensure representation from the key disciplines
2. Establish patient centred goals
3. Bring opinions together openly
4. Agree an overall aim, next steps with time frames
5. Allocate responsibilities
6. Communicate/ document outputs

Written MDT communication

Written MDT documentation should be appropriately co-designed to capture all relevant information. All patient admissions should be clerked using a standardised proforma co-agreed and designed by the MDT with sections addressing trauma at other skeletal sites (besides the hip), nerve block use, osteoporosis management and orthogeriatric and orthopaedic senior reviews. Documents need to be available to the wider MDT.
Examples of documents/ proformas that can assist the delivery of care include:

- Structured admission clerking proforma
- Structured post-operative junior doctor review (for patients returning to the ward from theatre)
- Structured falls and bone health assessments

Examples of documents are available on the National Hip Fracture Database (NHFD):
https://www.rcplondon.ac.uk/projects/outputs/national-hip-fracture-database-nhfd-improvement-repository

Electronic MDT communication

Smart Phones have made radio pagers redundant. All staff should have access to a Smart Phone. Information can be managed securely through e.g. Siilo for patient specific information. WhatsApp can be used for team communication, but not patient specific information. Mobile phone numbers should be routinely shared within the MDT. Appropriate groups should be set-up.

Hospitals need to invest in electronic communication systems e.g. (i) to enable trauma wards to have live monitoring of theatre activity, to plan prompt transfers to and from theatre, and (ii) routine access to primary care records. Templated discharge summaries, e.g. with use of standardised phrases, can improve efficiency and increase communication at discharge.

Hip fracture patients are best managed on a hip fracture ward where all members of the MDT can be co-located and thus easily found for spontaneous communication, with nearby office space. Timetabling of consultant ward rounds and daily ward activities should be shared amongst the whole team and adhered to.

For peer review only

Action 3: Establish inter-disciplinary principles of working

Healthcare teams can be 'multidisciplinary ' or 'interdisciplinary'. Multi-disciplinary teams have separate roles and work within their own specific disciplines. Interdisciplinary teams work more closely together to deliver shared goals and some roles may overlap.

Where human resource permits, hip fracture care should be provided through a shared care model between orthopaedic and orthogeriatric services.

Orthopaedic, geriatric and anaesthetic specialist trainees should receive formal inter-disciplinary training to ensure mutual understanding of each other's roles, e.g. geriatric trainees should attend a trauma meeting and a hip fracture theatre session, whilst orthopaedic trainees should join an orthogeriatric ward round and geriatrician lead white board rounds. Similarly, an orthogeriatric trainee should spend time with an orthopaedic physiotherapist whilst the anaesthetic trainee should join the orthogeriatric ward round. The ultimate goal of interdisciplinary training is to ensure mutual understanding and professional support between roles.

All trainees should attend clinical governance meetings to understand governance structured necessary to support a complex pathway of patient care.

Examples of inter-disciplinary upskilling include:

- Nurses should be upskilled by physiotherapists to confidently mobilise patients post-operatively
- Emergency department doctors should be upskilled by anaesthetists to provide emergency nerve blocks
- Physiotherapists should be upskilled by occupational therapists to assist discharge planning
- Orthopaedic trainees should be upskilled by orthogeriatricians to assess cognitive capacity to consent

Agree cross-cover arrangements within clinical competencies, e.g. physiotherapy and occupational therapy co-working, medical and surgical junior doctors cross-covering, nursing staff empowered to mobilise patients.

Action 4: Establish collaborative MDT team leadership

Orthogeriatric (1), orthopaedic (2), anaesthetic (3) consultant hip fracture leads should have their role defined within their job plan. The orthopaedic and/or orthogeriatric consultant should identify as clinical (co-)lead for hip fracture services.

Strong clinical leadership is essential to co-ordinate effective MDT hip fracture care delivery. Formal leadership training is strongly encouraged.

A physiotherapy (4), nursing (5), occupational therapy (6) and pharmacy (7) discipline lead

For peer review only

for hip fracture care should be appointed.

All seven of these professionals have responsibility to attend clinical governance meetings and act as conduit for feedback from and to their individual specialist teams.

All disciplinary leads should consider succession planning and discuss this with the leadership team to manage leadership transitions smoothly and efficiently (no discipline should be without hip fracture leadership). Aim for early identification of successors with structured mentorship with staged handover.

Identify a hospital board executive responsible for bidirectional communication with the hip fracture clinical lead, who will champion hip fracture care at board level. All seven discipline-specific hip fracture leads should have met with the hospital board executive. The primary point of contact for the hospital board executive should be the hip fracture service clinical lead.

Action 5: Jointly agree the goals of the team and strategies to achieve these goals

Define MDT goals by jointly reviewing NHFD performance, bench-marked KPIs, and how these compare against national averages, as well as REDUCE toolkit targets and any specialty-specific internal audit/ QI data. It's really important to get all members of the team in the room and to hear the voices and perspectives of each MDT discipline with no one specialty dominating the conversation. The clinical lead for hip fracture services holds the responsibility for coordinating this process.

Strategies to improve services should be structured into short, medium and long-term approaches and achievements routinely reviewed at clinical governance and morbidity and mortality (M&M) meetings with plans and progress clearly communicated to the hip fracture service hospital board executive. The REDUCE toolkit is designed to facilitate each hospital to develop their own individualised strategies towards service improvement.

For peer review only

Supplementary Material: Consolidated criteria for reporting qualitative studies (COREQ): 32-item checklist

Please indicate in which section each item has been reported in your manuscript. If you do not feel an item applies to your manuscript, please enter N/A.

For further information about the COREQ guidelines, please see Tong et al., 2017: https://doi.org/10.1093/intqhc/mzm042

No.	Item	Description	Section #
**Domain 1: Research team and reflexivity**			
Personal characteristics			
1.	Interviewer/facilitator	Which author/s conducted the interview or focus group?	p. 10
2.	Credentials	What were the researcher's credentials? *E.g. PhD, MD*	p. 1
3.	Occupation	What was their occupation at the time of the study?	p.10
4.	Gender	Was the researcher male or female?	N/A
5.	Experience and training	What experience or training did the researcher have?	p. 1 /ORCID
Relationship with participants			
6.	Relationship established	Was a relationship established prior to study commencement?	No
7.	Participant knowledge of the interviewer	What did the participants know about the researcher? *E.g. Personal goals, reasons for doing the research*	N/A
8.	Interviewer characteristics	What characteristics were reported about the interviewer/facilitator? *E.g. Bias, assumptions, reasons and interests in the research topic*	Yes, acknowledgement
**Domain 2: Study design**			
Theoretical framework			
9.	Methodological orientation and theory	What methodological orientation was stated to underpin the study? *E.g. grounded theory, discourse analysis, ethnography, phenomenology, content analysis*	p. 11
Participant selection			
10.	Sampling	How were participants selected? *E.g. purposive, convenience, consecutive, snowball*	p. 9
11.	Method of approach	How were participants approached? *E.g. face-to-face, telephone, mail, email*	p. 9
12.	Sample size	How many participants were in the study?	p. 10
13.	Non-participation	How many people refused to participate or dropped out? What were the reasons for this?	p. 10
Setting			
14.	Setting of data collection	Where was the data collected? *E.g. home, clinic, workplace*	p. 10
15.	Presence of non-participants	Was anyone else present besides the participants and researchers?	No

Formatted: Font: 6 pt

16.	Description of sample	What are the important characteristics of the sample? *E.g. demographic data, date*	p. 9, p. 12
Data collection			
17.	Interview guide	Were questions, prompts, guides provided by the authors? Was it pilot tested?	p. 10 - 11
18.	Repeat interviews	Were repeat interviews carried out? If yes, how many?	N/A
19.	Audio/visual recording	Did the research use audio or visual recording to collect the data?	p. 11
20.	Field notes	Were field notes made during and/or after the interview or focus group?	No
21.	Duration	What was the duration of the interviews or focus group?	p. 10
22.	Data saturation	Was data saturation discussed?	p. 10
23.	Transcripts returned	Were transcripts returned to participants for comment and/or correction?	No
**Domain 3: analysis and findings**			
Data analysis			
24.	Number of data coders	How many data coders coded the data?	p. 11
25.	Description of the coding tree	Did authors provide a description of the coding tree?	p. 11
26.	Derivation of themes	Were themes identified in advance or derived from the data?	p. 11
27.	Software	What software, if applicable, was used to manage the data?	p. 11
28.	Participant checking	Did participants provide feedback on the findings?	No
Reporting			
29.	Quotations presented	Were participant quotations presented to illustrate the themes / findings? Was each quotation identified? *E.g. Participant number*	Boxes 1 - 3
30.	Data and findings consistent	Was there consistency between the data presented and the findings?	Yes
31.	Clarity of major themes	Were major themes clearly presented in the findings?	Yes
32.	Clarity of minor themes	Is there a description of diverse cases or discussion of minor themes?	Yes

Developed from: Allison Tong, Peter Sainsbury, Jonathan Craig, Consolidated criteria for reporting qualitative research (COREQ): a 32-item checklist for interviews and focus groups, International Journal for Quality in Health Care, Volume 19, Issue 6, December 2007, Pages 349–357, https://doi.org/10.1093/intqhc/mzm042

For peer review only
